# A tale of two drug targets: the evolutionary history of BACE1 and BACE2

**DOI:** 10.3389/fgene.2013.00293

**Published:** 2013-12-17

**Authors:** Christopher Southan, John M. Hancock

**Affiliations:** ^1^IUPHAR Database and Guide to Pharmacology Web Portal Group, University/BHF Centre for Cardiovascular Science, Queen's Medical Research Institute, University of EdinburghEdinburgh, UK; ^2^Department of Physiology, Development and Neuroscience, University of CambridgeCambridge, UK

**Keywords:** BACE1, BACE2, Alzheimer's Disease, type II diabetes, protein family evolution

## Abstract

The beta amyloid (APP) cleaving enzyme (BACE1) has been a drug target for Alzheimer's Disease (AD) since 1999 with lead inhibitors now entering clinical trials. In 2011, the paralog, BACE2, became a new target for type II diabetes (T2DM) having been identified as a TMEM27 secretase regulating pancreatic β cell function. However, the normal roles of both enzymes are unclear. This study outlines their evolutionary history and new opportunities for functional genomics. We identified 30 homologs (UrBACEs) in basal phyla including Placozoans, Cnidarians, Choanoflagellates, Porifera, Echinoderms, Annelids, Mollusks and Ascidians (but not Ecdysozoans). UrBACEs are predominantly single copy, show 35–45% protein sequence identity with mammalian BACE1, are ~100 residues longer than cathepsin paralogs with an aspartyl protease domain flanked by a signal peptide and a C-terminal transmembrane domain. While multiple paralogs in *Trichoplax* and *Monosiga* pre-date the nervous system, duplication of the UrBACE in fish gave rise to BACE1 and BACE2 in the vertebrate lineage. The latter evolved more rapidly as the former maintained the emergent neuronal role. In mammals, Ka/Ks for BACE2 is higher than BACE1 but low ratios for both suggest purifying selection. The 5' exons show higher Ka/Ks than the catalytic section. Model organism genomes show the absence of certain BACE human substrates when the UrBACE is present. Experiments could thus reveal undiscovered substrates and roles. The human protease double-target status means that evolutionary trajectories and functional shifts associated with different substrates will have implications for the development of clinical candidates for both AD and T2DM. A rational basis for inhibition specificity ratios and assessing target-related side effects will be facilitated by a more complete picture of BACE1 and BACE2 functions informed by their evolutionary context.

## Introduction

The amino acid aggregates of Aβ peptides forming the major component of plaques characteristic of Alzheimer's Disease (AD) result from N-terminal cleavage of the amyloid precursor protein (APP) (Goedert and Spillantini, 2006) mediated by an aspartyl protease referred to as Beta-site APP Cleaving Enzyme 1 (BACE1) (Hussain et al., 1999; Sinha et al., 1999; Vassar et al., 1999; Yan et al., 1999). BACE1 cleaves APP between residues 671 and 672 leading to extracellular release of beta-cleaved soluble APP. The cell-associated carboxy-terminal fragment of APP is subsequently released by the gamma-secretase complex of proteins facilitating intra-membrane proteolysis by the presenilin proteins, PSEN1 and PSEN2 (Selkoe and Wolfe, 2007). Because neurotoxic Aβ peptide production needs the combination of BACE1 and gamma secretase, both have been intensively pursued as AD drug targets for well over a decade (Durham and Shepherd, 2006; Olson and Albright, 2008; Karran et al., 2011). For gamma secretase inhibitors, recent clinical trial results have been disappointing, although second-generation, Notch-sparing compounds may still hold promise (Xia et al., 2012).

Pursued by many of the same companies in parallel to gamma secretase, global efforts toward BACE1 inhibition have been intense (Probst and Xu, 2012), culminating in the progression of several optimized leads toward their clinical phases (Stamford et al., 2012; Weiss et al., 2012; Hilpert et al., 2013). The prospects for success compared to gamma secretase seem more hopeful for three reasons. Firstly, the mouse BACE1 knock-out remains the only one of nearly 2000 tested viable gene ablations that robustly alter brain Aβ levels (Toyn et al., 2010). Secondly, LY2811376 (PubChem CID 44251605) has provided a proof-of-concept Aβ reduction in human clinical studies, despite progression being halted because of probable compound-specific (rather than target-specific) retinal toxicology (May et al., 2011). Thirdly, there has been new, indirect genetic target validation in the form of an A673T mutation in APP that was AD-protective due to reduced BACE1 cleavage (Jonsson et al., 2012).

Within a year of the discovery of BACE1 its paralog BACE2 (Q9Y5Z0) was also published by multiple groups (Acquati et al., 2000; Farzan et al., 2000; Hussain et al., 2000). This has 50% identity to BACE1 over 518 residues in humans. The next-highest alignment score against human proteins is cathepsin E (CATE, P14091) with 27% identity over 396 residues. A phylogenetic analysis has established that the BACEs are parologous to pepsins and cathepsins, although they may have shared origins with homologs found in the marine proteobacterian genus Shewanella (Rawlings and Bateman, 2009). The bi-lobed tertiary structure of the A1 proteases suggests this family has arisen from a duplication and fusion event. Even though the two symmetrical lobes of the PDB structures have recognizably similar folds, the residual internal sequence similarity from the ancient duplication is restricted to the short motif around each active site Asp.

Since investigations on the normal roles of BACE1 and BACE2 are too extensive to review here (particularly for BACE1) we present just a selection of citations to give an overview (Table 1).

**Table 1:** **Summaries of findings related to normal functions of BACE1 (Upper section) and BACE2 (lower section) in chronological order (KO = gene knock-out)**.

**Observation**	**References**
**BACE1**	
KO-mice showed subtle neurochemical deficits and behavioral changes	Dominguez et al., 2005
Cleaved APP ectodomain involvement in normal nerve cells and Aβ peptides dampening neuronal hyperactivity	Ma et al., 2007
Sixty-eight epithelial cell line substrates detected, many membrane-anchored and involved in contact-dependent intercellular communication	Hemming et al., 2009
Voltage-gated sodium channel subunits (SCN4B, O60939 and related subunits) substrates for regulation of Nav1 channel metabolism	Kovacs et al., 2010
Neuregulin, NRG1, Q022979, substrate for control of nerve cell myelination	Fleck et al., 2012
Amyloid-like protein 2 (APP2, Q06481) substrate for ectodomain fragments	Hogl et al., 2011
Brain substrates in inhibitor-treated and KO mice involved in neurites and synapses	Kuhn et al., 2012
Thirteen non-amyloidogenic substrates reviewed	Dislich and Lichtenthaler, 2012
Pancreatic ectodomain shedding regulates broad set of β-cell-enriched substrates	Stützer et al., 2013
Zebrafish KO indicates substrates related to neurite outgrowth and axon guidance, including plexin A3, B0S5N4, and glypican-1 (F1QCC6)	Hogl et al., 2013
Zebrafish KO shows peripheral hypomyelination	Van Bebber et al., 2013
**BACE2**	
Processes APP at the beta-secretase site	Hussain et al., 2000
Tissue distribution implies functions distinct from neuronal BACE1	Sun et al., 2005
KO mice normal but neonatal mortality increase in BACE1/2 double-KO	Dominguez et al., 2005
Processes APP but reduces Aβ production	Sun et al., 2006
Secretase of the plasma membrane protein TMM27 (Q9HBJ8) in mice and in human β cells	Esterhazy et al., 2011
Pancreatic ectodomain shedding regulates narrow set of β-cell-enriched substrates, including SEZ6L (Q9BYH1) and SEZ6L2 (Q6UXD5)	Stützer et al., 2013
Role in processing mouse pigment cell-specific Melanocyte Protein, PMEL, Q60696	Rochin et al., 2013
Zebrafish KO melanocyte migration phenotype. Double KO (Bace1^−/−^; Bace2^−/−^) viable and does not enhance the single mutant phenotypes, indicating non-redundant functions in fish	Van Bebber et al., 2013

The current picture of BACE function is increasingly complex but trends can be discerned. The first trend is that the continued confirmation of new *in vivo* substrates points toward pleiotropic roles. The second is that, while the picture of neuronal substrate processing for BACE1 and pancreatic substrates for BACE2 holds true, there is increasing evidence of overlap. In particular, BACE1 functions may extend to non-CNS tissues and cell types in which the same and/or different substrates can be processed. The third trend is the emergence of role differences between humans, mice and fish.

A notable 2011 report unexpectedly promoted BACE2 to an equivalent drug target status to that which BACE1 had immediately acquired in 1999. Since TMM27 (Q9HBJ8) was shown to be a regulator of normal beta cell function the research team went on to show that insulin-resistant mice treated with a BACE2 inhibitor (CID 50938551) displayed both augmented β cell mass and improved control of glucose homeostasis due to increased insulin levels (Esterhazy et al., 2011). These findings therefore constituted an initial drug target validation of BACE2 inhibition for type II diabetes (T2DM). While the molecular mechanisms by which BACE2 deficiency or inhibition affect β cell function and proliferation are unknown, they may involve not only the stabilization of TMEM27 but additional BACE2 substrates (Stützer et al., 2013).

The double drug target status of the BACEs, together with the still-incomplete functional pictures of both enzymes, presents an opportunity for a phylogenomic investigation. This is facilitated by the increasing breadth (i.e., more species) and depth (i.e., more phyla) of completed genomes, draft assemblies and transcript data. For BACE1, the existence of sequence similarity matches in *Ciona intestinalis* and *Strongylocentrotus purpuratus* had already been noted (Stockley and O'Neill, 2008; Venugopal et al., 2008). Here we focus on the discovery of novel homologs from basal phyla. From finding a predominantly single-copy UrBACE in most eumetazoans we identify a major duplication event approximately corresponding to the origin of the jawed vertebrates (Gnathostomata). We also suggest that frequent duplication and loss events may have contributed to the evolution of this gene family.

## Methods

### Reference sequences and terminology

Detailed information on the search sequences can be found in the appropriate UniprotKB/Swiss-Prot records for BACE1_HUMAN (P56817) and BACE2_HUMAN (Q9Y5Z0). Additional information is available in the MEROPS peptidase database via the identifiers A01.004 (http://merops.sanger.ac.uk/cgi-bin/pepsum?mid=a01.004) and A01.041, respectively (Rawlings et al., 2008). Comparative genomic data can be accessed via the Ensembl entry points for ENSG00000186318 (http://www.ensembl.org/Homo_sapiens/Gene/Summary?g=ENSG00000186318;r=11:117156402-117186975 and ENSG00000182240 for BACE1 and BACE2, respectively (http://www.ensembl.org/Homo_sapiens/Gene/Summary?db=core;g=ENSG00000182240;r=21:42539728-42648524. In summary BACE1 is transcribed from 9 exons on human chromosome 11q23.3 and BACE2 from 9 exons on 21q22.3.

To reduce repetition we use the following terminology. While BACE is technically a BACE1 synonym (whose usage preceded the latter) we use the term BACE(s) to refer to the pan-vertebrate parologous pairs of BACE1 and BACE2. The term BACE-like is reserved for high-scoring similarity matches that we detected but that were not unequivocally assignable to either. Where our analysis has clearly resolved these to single (or low multiple) ORFs in basal phyla we use the term UrBACE (see Results).

### Sequence searching and checking

The utility of selected resources for the phylogenetic investigation of proteases and their substrates has been previously noted (Southan, 2007). We made use of Ensembl GeneTree as an automated starting point from which we manually checked selected ORFs and proceeded to search for new homologs in the sequence databases (Ruan et al., 2008). For gene predictions or transcript translations we inspected NCBI BLASTP output for similarity matches, truncations, insertions or deletions, using default search parameters. All BACE-like, genomic pipeline-predicted ORFs were checked by BLASTX searches against transcript collections. In a few cases GENESCAN runs on genomic DNA for *de novo* protein prediction extended the ORFs.

InterProScan was used to detect extended family matches, local domain matches, N-terminal patterns indicative of signal peptides, C-terminal transmembrane domains and to detect breaks in the global alignment profiles. Initially we were confounded by cases where direct THMM transmembrane and SignalP prediction indicated terminal TMs apparently missed by InterProScan. We eventually discovered that a cryptic licensing restriction in the online version of InterProScan meant sequences had to be changed by a single residue from identical UniProt entries before TMs were generated.

By performing TBLASTN searches of ORFs against both Expressed Sequence Tags (dbEST) and the Transcriptome Shotgun Assembly sequence division (TSA) we found many new BACE-like sequences. These included complete and partial cDNAs from basal phyla that do not yet have complete genome coverage. Such searches were also used to extend truncated genomic-predicted ORFs. The expanding coverage of mammals and major vertebrate phyla by Ensembl has produced dense coverage for BACEs via the automated population of GeneTree. We therefore focused our collation efforts on expanding the more sparsely populated deep phylogeny which is currently restricted to just *Ciona* in Ensembl.

We selected a limited number of close, cathepsin-like paralogs to these BACE-like sequences as out-group sequences to resolve the possible evolutionary patterns. These combined approaches allowed us to generate a large set of sequences most of which were novel. We also used representative human substrates of BACE1 and BACE2 to search against sets of predicted proteins from model organism genomes to identify their phylogenetic distributions. We included presenilin 1 (PSEN1), which is also associated with secretase action, via APP being a substrate. Since this is highly conserved with a deep phylogenetic pattern it served as a useful similarity score calibration.

### Alignment and phylogenetic analysis

The initial stages used COBALT for checking as the sequences were being iteratively assembled and cross-checked (Papadopoulos and Agarwala, 2007). The final alignments were made using PRANK (Löytynoja and Goldman, 2010). Following recommendations of a recent evaluation (Gonnet, 2012), phylogenetic analysis was carried out by applying BioNJ (Gascuel, 1997) (http://www.phylogeny.fr/version2_cgi/one_task.cgi?task_type=bionj) to PRANK protein alignments. Tree reconstructions were carried out using default parameters.

### Sequence blocks and gapped alignments

As an adjunct to phylogenetic relationships, multiple alignments can also be used to extract conserved sequence blocks. These were generated with Blockmaker and then analyzed by WebLogo to provide a comparative visual description of residue conservation at each position (Henikoff et al., 1995; Crooks et al., 2004). It should be noted that this approach is selective, compared to tree construction, in that gapped or very divergent sequences are excluded. Block sequence sections and individual residues from the logos were then mapped to a BACE1 2D transformation available in PDBSum (Laskowski, 2009). The important C-terminal domains of BACE-like sequences proved difficult to discern in global multiple alignments or block-type approaches. We addressed this by using the T-Coffee algorithm because this is optimized for gapping (Rausch et al., 2008).

### Ka/Ks analysis

Ka/Ks ratios were estimated on subsets of well-founded cDNAs of BACE1 and BACE2 separately. The cDNA sequences were derived only from mammals to minimise effects of multiple mutations (although these cannot be eliminated completely) and included only coding sequence. Ka/Ks calculations were carried out using SLAC (Kosakovsky Pond and Frost, 2005) for complete coding regions and for sub-regions corresponding to functional domains and to individual exons. Boundaries of functional domains and intron/exon boundaries were taken from the CCDS database (Pruitt et al., 2009)

### Data availability, re-use, updating, and connectivity

We have taken four complementary approaches to data sharing that we hope will ensure persistence, re-use, updating, and facilitate connectivity (Leebens-Mack et al., 2006; Stoltzfus et al., 2012; Drew, 2013). Firstly, we have used the supplementary data option provided by this journal, with the specific data being referred to in results. Secondly, we have made a deposition in TreeBase (Anwar and Hunt, 2009) of a Nexus file representing the alignment used for the phylogeny analysis. The permanent URL for this Nexus file is http://purl.org/phylo/treebase/phylows/study/TB2:S14732. As a third measure we have deposited the supplementary data on figshare as an open archive (Singh, 2011), at http://figshare.com/articles/Supplementary_Data_for_Southan_Hancock_BACE_evolution_paper/855620

It is important to note that the majority of the sequences we have collated are not yet available as stable protein accession numbers for full-length ORFs and their primary nucleotide records are spread across many database divisions. In addition, the rate of generation and revision (e.g., new cDNA and genome assembly updates) is such that our TreeBase deposition will become outdated. We have thus included (both in the supplementary data and the figshare link) a complete FASTA compilation of the sequences. These can either be used as-is for different alignment approaches or updated via new database searches.

Fourthly, we have submitted a representative complete cDNA and protein sequence of the *Ciona intestinalis* UrBACE to the Third Party Annotation (TPA) division of the European Nucleotide Archive (ENA). This was assembled using EST data to consolidate the gene prediction. This will have the accession number HE967761. We will ensure this is updated to link to the eventual PubMed ID, giving researchers the possibility of connecting to our work directly via a sequence search.

## Results

### Classifying BACE-like ORFs

The exercise of finding and checking new BACE-like proteins presented the following technical challenges:

- Discerning what data types for which organism were in which source and/or database division (e.g., cDNA data could be in mRNA(nr), dbEST, TSA, or all three).
- Ascertaining completion status at the genome assembly level and coverage at the transcript level (e.g., we could not easily resolve the *Monosiga ovata* EST-derived ORFs against the JGI *Monosiga brevicolis* genome-derived ORFs).
- Encountering the same or different ORFs from the same organism in multiple pipelines (e.g., JGI, BCM, UCSC, XP, Ensembl, RefSeq, and TrEMBL). It was often unclear which organisms were unique to which portals or which database records were transitively circular (i.e., cross-referenced back to the same primary sequence data) or derived via independent pipeline results.
- For both genomic prediction and cDNA data we commonly encountered the error types of terminal truncations, internal exon losses and chimeras.

Fortunately multiple alignments are tolerant of at least moderate gaps or truncations and can still be informative with respect to tree topology and branch lengths reflecting protein sequence similarity scores. Consequently, some partial sequences were used to populate otherwise sparse sections of the tree.

We classified sequences as BACE-like by two approaches. The first was identifying them as probable orthologs extending across major phyla. The second was discriminating them from cathepsins (i.e., as not cathepsin-like). Our triage utilized the following criteria:

1. Reciprocal BLAST similarity (i.e., using the query best match as a new database query). This always grouped BACEs at the top of the hit list.
2. Matches of ~35% identity or more, extending across the major part of the ORF without over-gapping (e.g., the *Planarium* sequence, with one of the lowest similarity scores in the set, matches human BACE1 with an *E*-value of 3e–58 representing 34% identity over 387 residues with 7% gaps).
3. The similarity scores to cathepsin homologs in any single species were distinctly lower (e.g., dropping to ~25% identity over ~350 residues with ~20% gapping).
4. Sequences were typically ~100 residues longer than cathepsin homologs.
5. A unique pattern of a predicted N-terminal signal peptide and a C-terminal transmembrane (CTM) either side of the protease domain was present. The CTM was absent from all analyzed cathepsin homologs while they typically also had N-terminal signal peptides.
6. Extended global alignment matches, together with individual diagnostic sections including at least one of the profiles for BACE (this was nearly always to BACE1 not BACE2).
7. Cathepsin homologs and vertebrate BACE2 sequences consistently showed two matches to the Prosite PS00141 regular expression diagnostic for the aspartyl active site. In contrast, all BACE1 and BACE-like sequences showed only the single proximal N-terminal match.
8. The gene structure of all BACE-like sequences, judged as complete ORFs, consisted of at least 9 exons. Most cathepsin homologs also showed this but some were single-exon.
9. The construction of provisional phylogenetic gene trees and testing different parameterizations, including cathepsins as out-groups, was used to support grouping into BACE-like or BACE sub-families.

Using the methods described we assembled the sequences specified in Table 2.

**Table 2:** **BACEs, BACE-like sequences and homologs used for phylogenetic analysis**.

**Short sequence name**	**Species name**	**Common name**	**NCBI Tax ID**	**UniProt ID**	**Protein length**
**Ur-BACE**					
Mono_ovat_A	*Monosiga ovata*	Choanoflagellate	81526		459
Mono_ovat_B	*Monosiga ovata*	Choanoflagellate	81526		541
Tric_adhe_A	*Trichoplax adhaerens*	Tricoplax	10228		545
Tric_adhe_B	*Trichoplax adhaerens*	Tricoplax	10228	B3RU95	505
Tric_adhe_C	*Trichoplax adhaerens*	Tricoplax	10228	B3RU94	428
Nema_vect	*Nematostella vectensis*	Sea Anemone	45351		479
Aipt_pall	*Aiptasia pallida*	Sea Anemone	12566		488
Hydr_mag	*Hydra magnipapillata*	Hydra	6085		412
Clyti_hem	*Clytia hemisphaerica*	Sponge	252671		474
Acro_mill	*Acropora millepora*	Stony Coral	45264		365
Clon_sine	*Clonorchis sinensis*	Oriental Liver Fluke	79923		506
Schi_japo	*Schistosoma japonicum*	Fluke	6182		488
Schi_mans	*Schistosoma mansoni*	Fluke	6183	G4VD03	507
Plan_schm	*Schmidtea mediterranea*	Planarium	79327		516
Capi_tela	*Capitella telata*	Polychaete annelid	283909		504
Mere_mere	*Meretrix meretrix*	Asiatic Hard Clam	291251		362
Rudi_phil	*Ruditapes philippinarum*	Manila Clam	129788		186
Vill_lien	*Villosa lienosa*	Freshwater Mussel	326719		326
Cras_giga	*Crassostrea gigas*	Pacific oyster	29159		520
Lott_giga	*Lottia gigantea*	Owl limpet	225164		495
Ilya_obso	*Ilyanassa obsoleta*	Eastern mudsnail	34582		250
Lymn_stagn	*Lymnaea stagnalis*	Pond Snail	6523		544
Eupr_scolo	*Euprymna scolopes*	Squid	6613		215
Stron_purp	*Strongylocentrotus purpuratus*	Sea Urchin	7668		538
Para_livi	*Paracentrotus lividus*	Purple Sea Urchin	7656		297
Sacc_kowa	*Saccoglossus kowalevskii*	Acorn Worm	10224		268
Cion_inte	*Ciona intestinalis*	Sea Squirt	7719		466
Cion_savi	*Ciona savignyi*	Sea Squirt	51511		458
Halo_rore	*Halocynthia roretzi*	Sea Squirt	7729		463
Bran_flor	*Branchiostoma floridae*	Amphioxus	7739	C3ZMY0	493
Petr_mari	*Petromyzon marinus*	Lamprey	7757		406
**BACE1**					
Hum_BACE1	*Homo sapiens*	Human	9606	P56817	501
Mouse_BACE1	*Mus musculus*	Mouse	10090	P56818	501
Rat_BACE1	*Rattus norvegicus*	Rat	10116	P56819	501
Dog_BACE1	*Canis familiaris*	Dog	9615		501
Bov_BACE1	*Bos taurus*	Cow	9913	Q2HJ40	501
Mono_dome_BACE1	*Monodelphis domestica*	Opossum	13616		466
Ailu_melan_BACE1	*Ailuropoda melanoleuca*	Panda	9646		501
Orin_anat_BACE1	*Ornithorhynchus anatinus*	Platypus	9258		475
Gall_Gal_BACE1	*Gallus gallus*	Chicken	9031		426
Taen_gutt_BACE1	*Taeniopygia guttata*	Zebra Finch	59729		522
Xeno_trop_BACE1	*Xenopus tropicalis*	Western clawed frog	8364	Q0P4T5	502
Anol_caro_BACE1	*Anolis carolinensis*	Lizard	28377		318
Pelo_sine_BACE1	*Pelodiscus sinensis*	Chinese Soft- Shelled Turtle	13735		484
Chry_pict_BACE1	*Chrysemys picta bellii*	Western painted Turtle	8478		437
Dani_reri_BACE1	*Danio rerio*	Zebrafish	7955		531
Taki_rubr_BACE1	*Takifugu rubripes*	Fugu	31033		443
Tetr_nigr_BACE1	*Tetraodon nigroviridis*	Pufferfish	99883	Q4RYS5	448
Gast_acul_BACE1	*Gasterosteus aculeatus*	Sticklback	69293		490
Oryz_lati_BACE1	*Oryzias latipes*	Medaka	8090		442
Lat_chal_BACE1	*Latimeria chalumnae*	Coelocanth	7897		488
Gadu_morh_BACE1	*Gadus morhua*	Cod	8049		481
Oreo_nilo_BACE1	*Oreochromis niloticus*	Nile Tilapia	8128		488
Salm_salm_BACE1	*Salmo salar*	Atlantic Salmon	8030		348
Leuc_erin_BACE1	*Leucoraja erinacea*	Little Skate	7782		203
Squa_acan_BACE1	*Squalus acanthias*	Spiney Dogfish	7797		202
**BACE2**					
Hum_BACE2	*Homo sapiens*	Human	9606	Q9Y5Z0	518
Mouse_BACE2	*Mus musculus*	Mouse	10090	Q9JL18	515
Rat_BACE2	*Rattus norvegicus*	Rat	10116	Q6IE75	514
Dog_BACE2	*Canis familiaris*	Dog	9615		422
Cow_BACE2	*Bos taurus*	Cow	9913		473
Mono_dome_BACE2	*Monodelphis domestica*	Opossum	13616		531
Orni_anat_BACE2	*Ornithorhynchus anatinus*	Platypus	9258		423
Ailu_melan_BACE2	*Ailuropoda melanoleuca*	Panda	9646		426
Xeno_laev_BACE2_A	*Xenopus laevis*	African Clawed Frog	8355	Q7T0Y2	500
Xeno_laev_BACE2_B	*Xenopus laevis*	African Clawed Frog	8355	Q6PB20	499
Xeno_trop_BACE2	*Xenopus tropicalis*	Western Clawed Frog	8364	B4F734	499
Anol_carol_BACE2	*Anolis carolinensis*	Lizard	28377		417
Amb_tigr_BACE2	*Ambystoma tigrinum tigrinum*	Eastern tiger salamander	43116		337
Pelo_sine_BACE2	*Pelodiscus sinensis*	Chinese Soft-Shelled Turtle	13735		415
Chry_pict_BACE2	*Chrysemys picta bellii*	Western painted Turtle	8478		438
Gall_gall_BACE2	*Gallus gallus*	Chicken	9031		416
Taen_gutt_BACE2	*Taeniopygia guttata*	Zebra Finch	59729		441
Dani_reri_BACE2	*Brachydanio rerio*	Zebrafish	7955		503
Taki_rubr_BACE2	*Takifugu rubripes*	Fugu	31033		470
Tetr_nigr_BACE2	*Tetraodon nigroviridis*	Spotted Green Pufferfish	99883		472
Oryz_lati_BACE2	*Oryzias latipes*	Medaka	8090		437
Gast_acul_BACE2	*Gasterosteus aculeatus*	Sticklback	69293		508
Gadu_morh_BACE2	*Gadus morhua*	Cod	8049		497
Lati_chum_BACE2	*Latimeria chalumnae*	Coelocanth	7897		382
Oreo_nilo_BACE2	*Oreochromis niloticus*	Nile Tilapia	8128		509
Onco_myki_BACE2	*Oncorhynchus mykiss (+nerka)*	Rainbow Trout	8022		406
Salm_sala_BACE2	*Salmo salar*	Atlantic Salmon	8030		230
**BASAL CATHEPSINS**					
Plan_schm_cath01	*Schmidtea mediterranea*	Planarium	79327		389
Tric_adhe_cath01	*Trichoplax adhaerens*	Tricoplax	10228	B3RK44	383
Bran_flor_cath01	*Branchiostoma floridae*	Amphioxus	7739	C3YBT8	423
Mono_ovat_cath01	*Monosiga ovata*	Choanoflagellate	81526		381
Nema_vect_cath01	*Nematostella vectensis*	Sea Anemone	45351		370
Stro_purp_cat01	*Strongylocentrotus purpuratus*	Sea Urchin	7668		313
Cion_inte_cath01	*Ciona intestinalis*	Sea Squirt	7719		385
Cion_sauv_cath01	*Ciona savignyi*	Sea Squirt	51511	H2ZA35	370

### Negative and borderline results

With the caveat of data incompleteness in organisms represented only by draft genomes, BACE-like proteins were not detected (i.e., the BACE1 search probe matched only cathepsins at ~25% identity) in the folowing: *Saccharomyces cerevisiae*, *Caenorhabditis elegans, Drosophila melanogaster, Daphnia pulex, Arabidopsis thaliana, Amphimedon queenslandica*, *Dictyostelium discoideum*, *Plasmodium falciparum*, and *Strigamia maritima*. In addition, by using the taxonomic filters on the UniProt BLAST options, the entire protein collections from bacteria, plants, fungi, archaea and nematodes were also found to be negative. In the light of these results we dispute the published claim of the discovery of a BACE-like sequence in *Drosophila melanogaster* and the consequent annotation as such in the protein databases (Carmine-Simmen et al., 2009). The sequence in question (Q9VLK3) fails all the criteria we described above and, on the basis of similarity, the most plausible classification is a lysosomal cathepsin. This is regardless of whether the enzyme may exibit the beta-secretase activity of APP-clipping under certain experimental conditions, as has been reported for other cathepsins (Schechter and Ziv, 2011).

The following database matches were recorded as borderline but, because of low identity scores, truncations and absence of corroborative data, they were not included in the multiple alignments. A partial sequence (GAA56694) from the Liver Fluke *Clonorchis sinensis* gives reciprocal BACE1 scores but has no diagnostic matches in InteProScan and the primary data was not retrievable from the genome portal. The marine metagenome partial ORF EBK78785 also has BACE1 as top reciprocal match and a C-terminal TM domain but it has only 27% identity with extensive gapping and neighbor matches indicative of it being an algal protein.

### Identification of sequence features

**Figure 1:**
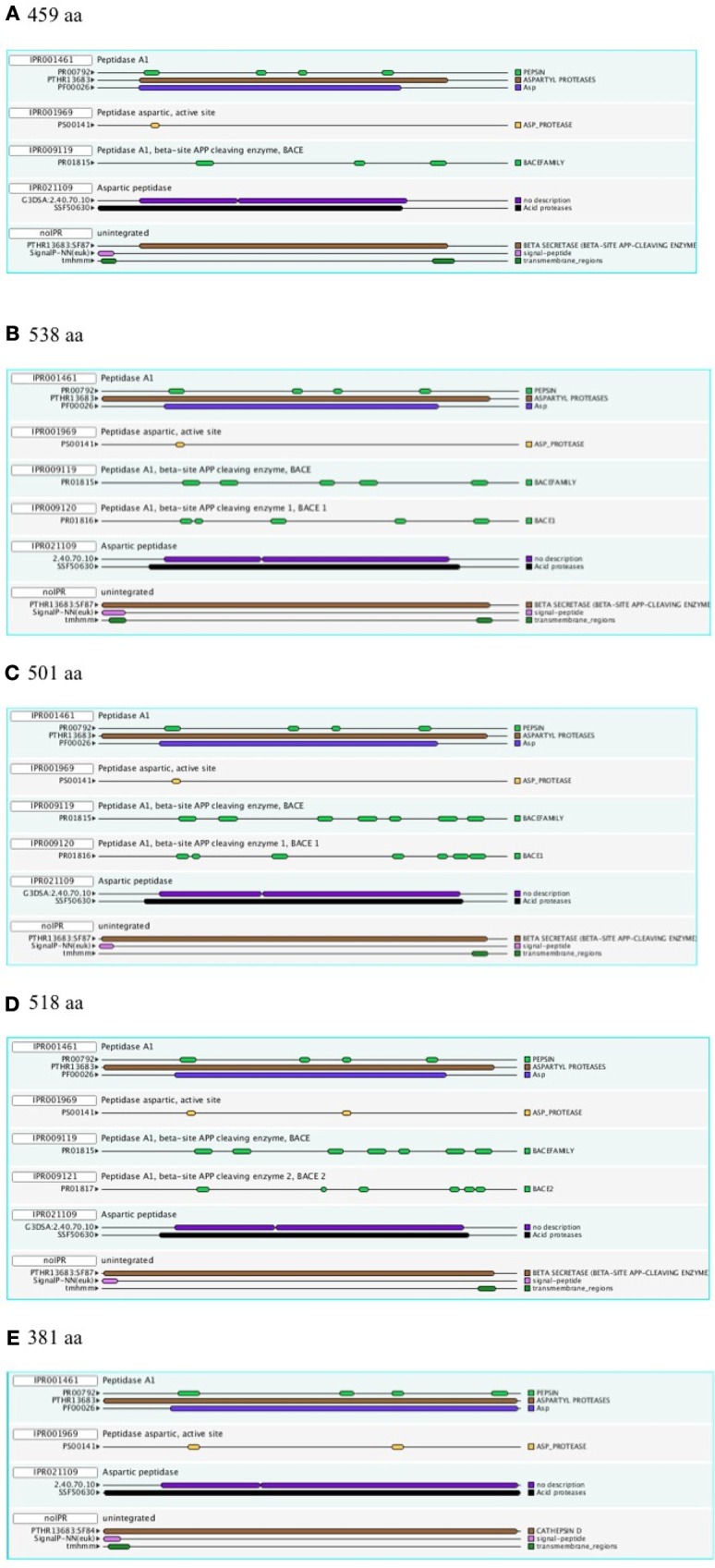
**InterProScan Sequence Features**. Representative examples are shown for two Ur-BACEs **(A)** *Monosiga ovata* A (Mono_ovat_A), **(B)** *Strongylocentrotus purpuratus* (Stron_purp), **(C)** human BACE1 (Hum_BACE1) **(D)** human BACE2 (Hum_BACE2), and **(E)** a cathepsin from *Monosiga ovata* (Mono_ovat_cath01). The protein length is indicated.

Figure 1 shows a representative set of InterProScan outputs for two Ur-BACEs, the human BACEs and a cathepsin.

**Figure 2:**
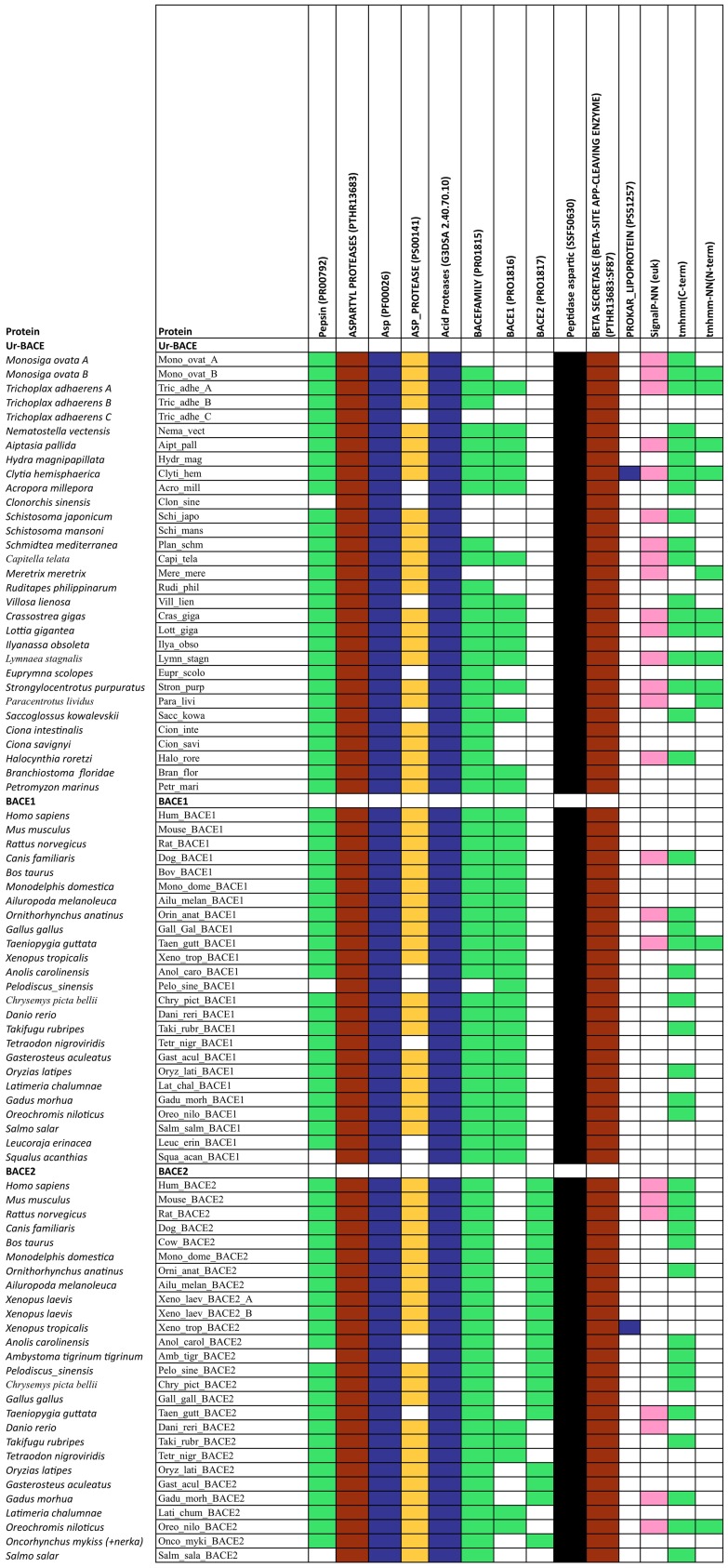
**Summary of InterProScan pattern hits for the proteins included in Table 2**.

The diagnostic utility of InterProScan results is not only for family and subfamily memberships but also for judging sequences to be full-length. As expected, the pepsin/cathepsin architecture is confirmed by all the global alignment matches. Three of the PRINTS local alignment patterns proved useful to distinguish between BACE family (PRO815), BACE1 (PRO1816), and BACE2 (PRO1817) (Attwood et al., 2012). Notably, the specificity of the matches extends to the Ur-BACEs, even though the profile is historically compiled from mammalian sequences. Ur-BACEs predominantly matched the BACE1 profile and none the BACE2 profile. For sequences with complete N-terminals, InterProScan detected either a tmhmm (~50%) or a SignalP (~30%) hit, and sometimes both. We interpret this as indicating a secretion-associated signal peptide cleavage as a universal feature. Similarly, all UrBACEs indicated the presence of a C-terminal transmembrane section. We provide a summary of matching across the entire sequence set in Figure 2.

### Extended phylogeny

**Figure 3:**
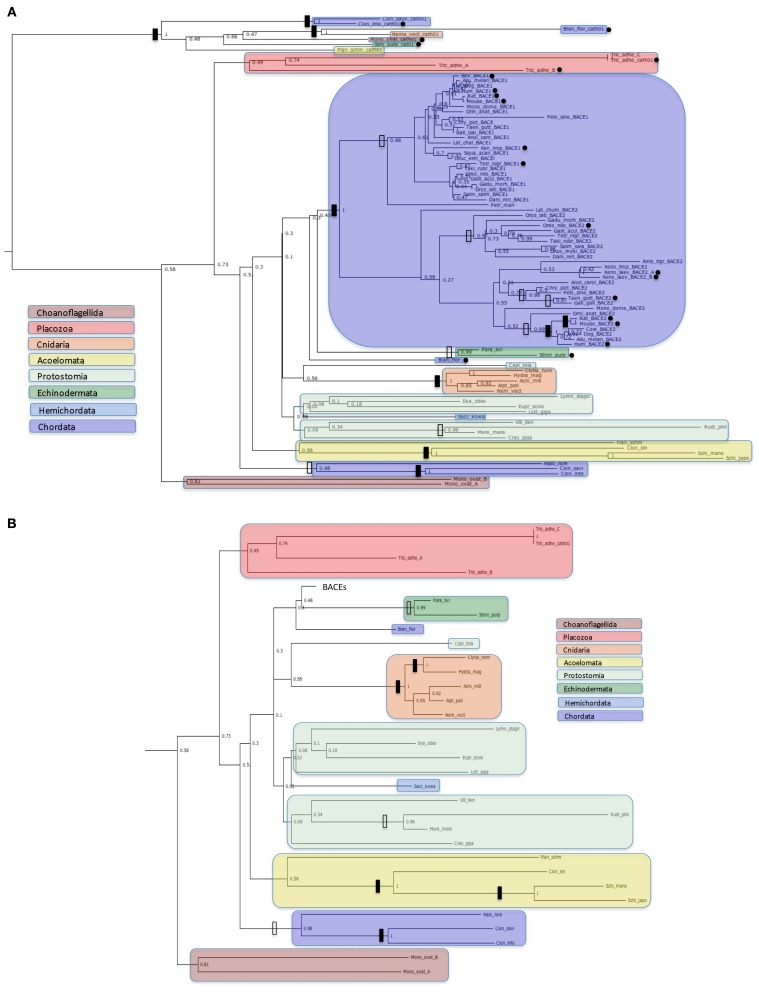
**Protein trees of BACE protein sequences**. Sequences are labeled as in Table 2. Black dots indicate sequences with accession numbers for complete ORFs in Table 2. Solid vertical bars represent bootstrap values of 100% and open bars represent bootstrap values of 95% or greater but less than 100%. Sequences corresponding to high order taxa are enclosed in colored boxes as indicated by the legends in the figures. **(A)**: All sequences analyzed; **(B)**: Ur-BACE sequences only.

A gene tree of the entire set of protein sequences from Table 2 is shown in Figure 3A. The UrBACE sequences alone are shown in Figure 3B. The alignment underlying this analysis is available as a Nexus file at http://purl.org/phylo/treebase/phylows/study/TB2:S14732 and both sets of sequences are supplied in FASTA format in the Supplementary Data file.

Figure 3 shows four distinct clusters with the two vertebrate BACEs generally well resolved from the Ur-BACEs and their cathepsin homologs. While we have restricted the vertebrate sequences to major groupings (more orthologs can be accessed via Ensembl GeneTree if required) they recapitulate expected features. For example, BACEs from the new Turtle genomes cluster with birds, as concluded in the recent publication (Shaffer et al., 2013). However, there are departures from a simple evolutionary model based on duplication in the fish linage. In the vertebrate clades, *Xenopus tropicalis* contains a clearly identifiable BACE1 and BACE2. However, the two homologs identified in *X. laevis* both clustered with *X. tropicalis* BACE2, share 90% sequence identity as paralogs, and are therefore designated as BACE2a and BACE2b.

Branch lengths within the BACE2 family appear longer than those for BACE1. To quantify this we compared equivalent sequence distances between the protein sets. The two *X. laevis* BACE2s were excluded from the analysis, as was the *P. marinus* UrBACE although this clustered with the duplicated BACEs. The average sequence difference for BACE1s was 0.317 whereas that for BACE2s was 0.551. The mean BACE2/BACE1 ratio for all comparisons was 2.36. Of 190 comparisons 150 showed greater distances for BACE2 than BACE1 while 40 showed a greater distance for BACE1 than BACE2. This suggests that BACE2 protein sequences have evolved more rapidly than BACE1. As this result is based on protein sequences, it likely suggests that purifying selection has acted more strongly on BACE1 than BACE2. The full distance matrix for the data set as generated by BIONJ is available in the Supplementary Data file.

The UrBACE sequences lie outside the two well-supported BACE clades with the exception of *Petromyzon marinus* UrBACE, which clusters close to the BACE1 grouping. Notably, two UrBACE sequences were identified for *Monosiga ovata* and three for *Trichoplax adhaerens*. The latter can be localized as an ordered cluster in the genome (Ensembl scaffold_4:758759-790622) but since none are complete ORFs in the browser mark-up (although our version of Tric_adhe_A was extended to 545 residues by *ab initio* gene prediction) the exon pattern and possible duplication history of the three is unclear. Both of these UrBACE paralogous sets have long branch lengths (e.g., the *Monosiga* pair shares only ~45% identity) suggesting that these duplications are ancient events independent of the duplication event giving rise to the BACE1 and BACE2 families. Amongst UrBACEs, most major groupings are supported, although protostomes do not resolve as a monophyletic group. An anomalous result is the high bootstrap value separating the tunicates (*Ciona* and *Halocynthia*) not only from other chordates but from all other UrBACEs. This may suggest that these sequences derive from a different duplication lineage to the other UrBACEs and the BACEs.

### Blocks analysis

**Figure 4:**
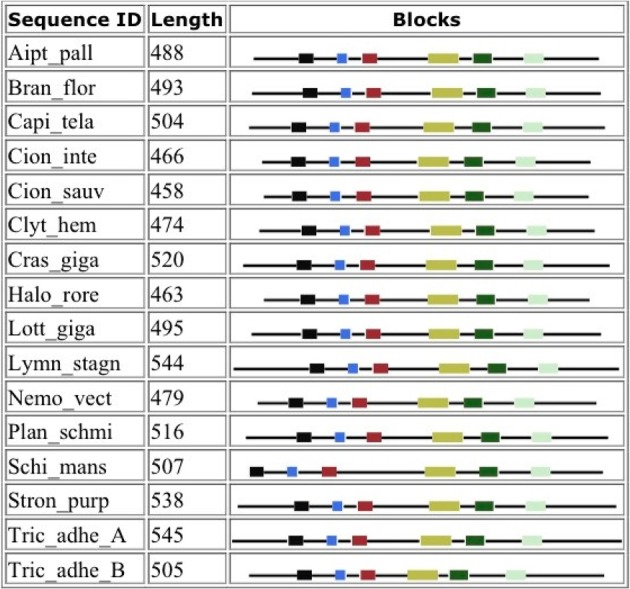
**Blocks generated from the UrBACE sequences**. The six conserved regions are shown in N-terminal to C-terminal order.

**Figure 5:**
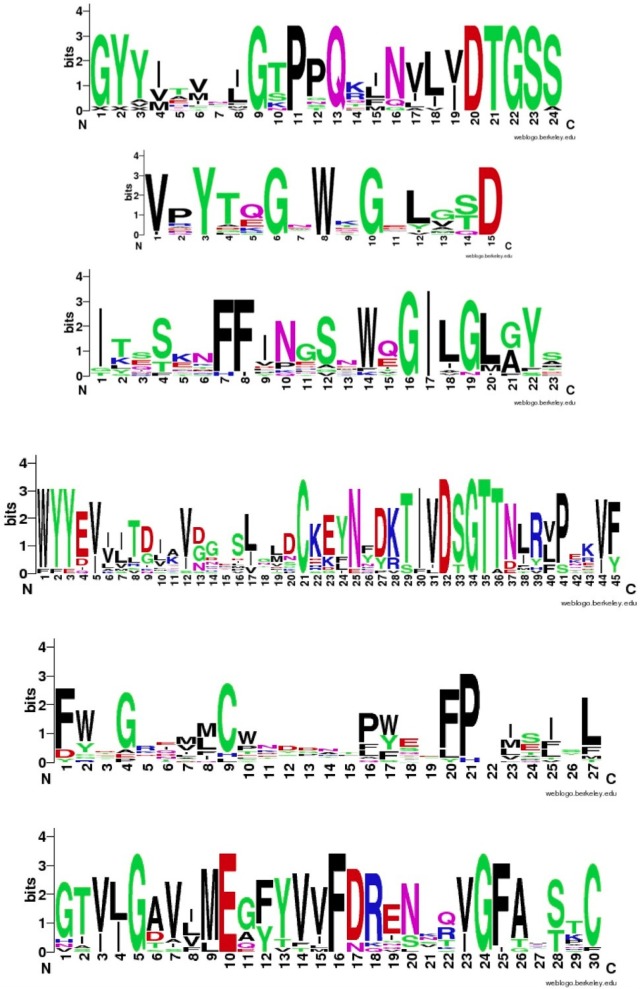
**Sequence Logos for the six conserved UrBACE blocks in N-terminal to C-terminal order (with spacing shown in Table 2)**. Residue letter height is an index of conservation.

**Figure 6:**
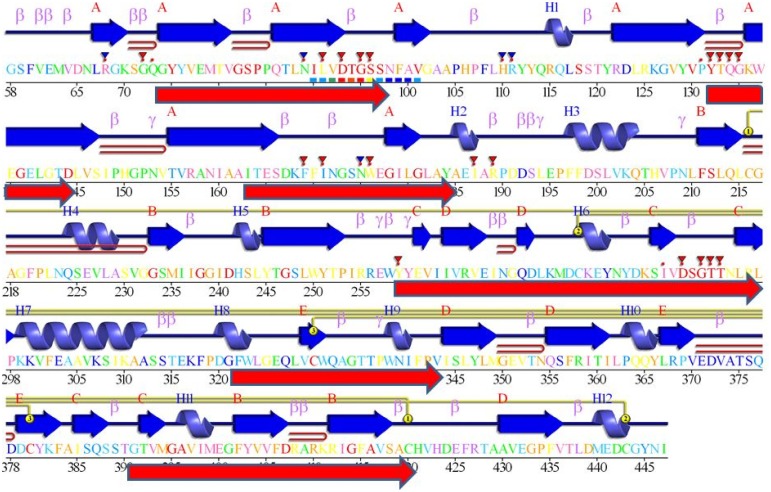
**The blocks from Figure 3 and Logos from Figure 4, aligned against a BACE1 PDBSum entry**. Among the many entries with different ligands the longest sequence was chosen, 3lpi. The 2D display is shaded red to purple for conserved residues but note these are derived from BACE1 alignments (i.e., do not include UrBACEs). The secondary structure elements in dark blue and other marked features are described in the PDBSum features. The red arrows, added from this work, correspond to the six UrBACE sequence blocks.

Figure 4 shows that despite the divergence of their sequences conserved blocks are consistently spaced between the different BACEs. Exceptions are the insert in *Schistosoma mansoni* and deletion in *Trichoplax adhaerens* (Tric_adhe_B). The Sequence Logo results are shown in Figure 5 and the mapping of these onto a BACE1 PDB structure in Figure 6.

As expected, the catalytic sites correspond to two of these blocks (blocks 1 and 4). Blocks 2 and 3 align with the ligand contact shell, indicated by the red triangles, while the remaining two blocks are associated with internal fold positions but also include conserved cysteines involved in disulphide bonding. Inspection of the ConSurf option in PDBSum indicates that essentially all 6 UrBACE blocks and the conserved (purple) residues in Figure 5 correspond to buried internal parts of the structure. Note that this analysis provides different but complementary results to the InterProScan and phylogeny results. The former show matches to pre-existing sequences and recognize the terminal domain features that typically do not crystallize. The blocks analysis shows divergence among the UrBACEs despite the common fold architecture. However, despite the nominal presence of catalytic residues, the extensive insertions in the *Schistosoma japonicum* sequence (if confirmed via cDNA data) suggest it could be catalytically non-functional, which could explain the anomalous branch length in the phylogenetic tree.

**Figure 7:**
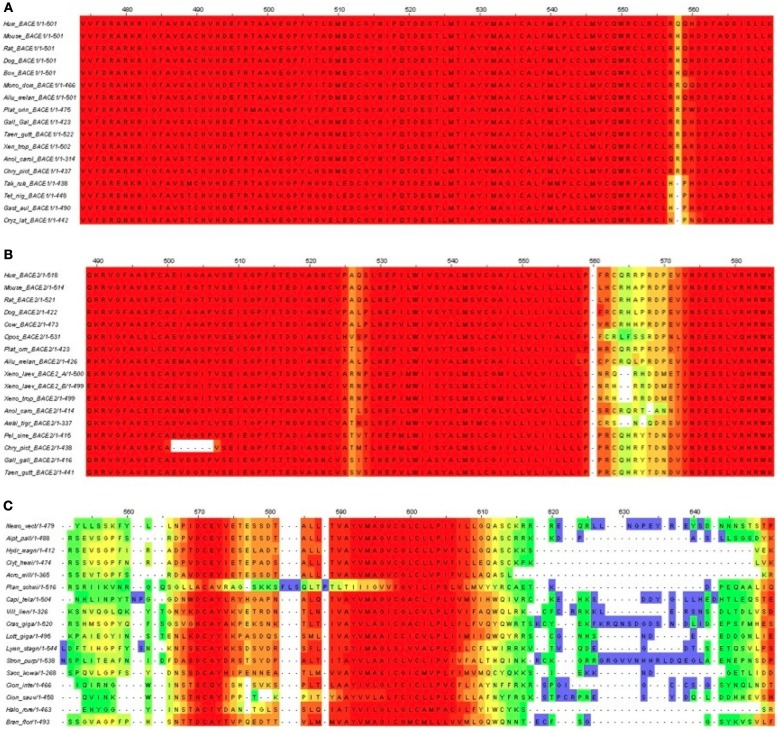
**T-Coffee alignments of C-termini for (A) BACE1, (B) BACE2, and (C) UrBACE**. The inputs were restricted to complete termini but also needed the removal of the *Monosiga* and *Trichoplax* sequences to give an informative result for panel **(C)**.

Many experiments have indicated the crucial role played by the BACE1 C-terminal, including S-palmitoylation at four Cys residues at the junction of the transmembrane and cytosolic domains. Although this has no discernible influence on BACE1 processing of APP in mouse, it traffics BACE1 dimers into cholesterol-rich lipid rafts (Vetrivel et al., 2009). While the question of the occupancy stoichiometry of the lipid chains between the Cys positions remains, there are no reports that BACE2 has the equivalent post-translational modification (PTM). For the UrBACE sequences block generation breaks down in this part of the multiple alignment although it did indicate all four Cys to be absolutely conserved in BACE1. While this suggests the palmitoylation could be pan-vertebrate, it raises the evolutionary question as to whether this is also a constitutive feature in BACE2 and/or the UrBACE. We explored the variation in the C-termini by using T-Coffee (Figure 7).

The results corroborate the very high conservation for the BACE1 C-terminal residues including the palmitoylated Cys candidates. It also indicates a possible single Cys candidate for BACE2 (at around 563 in the alignment) but this is absent in *Xenopus* and the gapping reflects low conservation. The difference for the UrBACEs is striking in that conservation falls off rapidly in the C-terminal cytoplasmic tail directly distal to the TM domain boundary. It can be noted here that the *Strongylocentrotus purpuratus* insert is confirmed by many ESTs (i.e., is not a gene prediction artifact). Significantly, a quartet of Cys candidates can be discerned, with the fourth one around position 615, despite the gapping in this region.

Notably, within the UrBACEs the block-type conservation co-exists with variation in conservation patterns and length at the termini. We interpret the blocks as being directly associated (as part of the active site) or indirectly (as the core buried 3D fold sections) associated with catalysis. In contrast the signatures of the signal peptide and the CTM are physical property-based. They are thus not only less conserved as sequences *per se* but also less constrained in position (even though the CTM needs to be a defined membrane-spanning length) as they are surface features. This also explains why UrBACEs vary by up to 100 residues in length.

### Ka/Ks analysis

The ratio of non-synonymous (protein sequence-changing) to synonymous (non-protein sequence-changing) mutation rates within a sequence, referred to as the Ka/Ks ratio, is an indicator of the strength of purifying selection. A low ratio indicates strong purifying selection; a value close to 1 indicates no selection, and a value above 1 positive selection (adaptive change of the sequence). Analysis was carried out on complete cDNA sequences and individual exons to reveal any differential selection along the length of the gene. The sets that could be analyzed (i.e., cDNAs of equal length) had to be different between BACE1 and BACE2, because of the limited availability of complete mammalian sequences. For BACE1, sequences from *H. sapiens, A. melanoleuca, S. scrofa. E. caballus, O. cuniculus, B. taurus, M. musculus*, and *R. norvergicus* were analyzed. For BACE2, these were from *H. sapiens, B. taurus, S. scrofa, M. musculus, R. norvegicus*, and *P. capensis*.

The values presented in Table 3 are the overall mean values for the sequence set generated by SLAC (Kosakovsky Pond and Frost, 2005). For the whole genes, Ka/Ks ratios were well below 1, indicating a predominant role for purifying selection in the evolution of mammalian BACE genes. Values for BACE2 were generally higher (i.e., less stringent selection) than for BACE1. This is consistent with more rapid evolution of BACE2 seen in the phylogenetic analysis, although the datasets here are not directly comparable due to their different compositions. Analysis of individual exons showed weaker purifying selection acting on the 5' (N-terminal) end of the gene, especially exon 1, while the central region of the gene showed low values (strongest selection) although the patterns differed between BACE1 and BACE2. Exon 8 of BACE2 in particular showed an elevated Ka/Ks value.

**Table 3:** **Results of Ka/Ks analysis for subsections of BACE1 and BACE2 cDNA sequences**.

**Sequence**	**BACE1**		**BACE2**	
	**AA Coords**	**Ka/Ks**	**AA Coords**	**Ka/Ks**
	**(Human protein)**		**(Human protein)**	
All	1–501	0.040	1–518	0.144
**DOMAINS**				
Signal	1–21	**0.284**	1–20	**0.156**
Propeptide	22–45	**0.127**	21–62	**0.282**
Chain	46–501	0.025	63–518	0.126
Extracellular	46–457	0.025	63–473	0.124
Transmembrane	458–478	0.000	474–494	**0.303**
Intracellular	479–501	**0.053**	495–518	0.093
**EXONS**				
Exon 1	1–87	**0.139**	1–104	**0.216**
Exon 2	88–117	0.000	105–134	0.105
Exon 3	118–189	0.014	135–206	0.060
Exon 4	190–235	0.032	207–249	0.057
Exon 5	236–280	0.007	250–294	0.040
Exon 6	281–314	0.000	295–328	0.059
Exon 7	315–364	0.000	329–378	0.027
Exon 8	365–422	0.008	379–435	**0.211**
Exon 9	423–501	0.028	436–518	0.140

Just coding sequences were used (i.e., excluding untranslated regions). Values marked in bold exceed the overall value for the complete cDNAs.

The signal and pro-peptide domains showed elevated Ka/Ks. In BACE2, but not in BACE1, high Ka/Ks was also observed for the C-terminal transmembrane domain. None of these Ka/Ks values approached 1, suggesting weaker purifying selection rather than positive selection when Ka/Ks ratio were higher. This is not unexpected, given that these surface-exposed sections are constrained more by the more general physical residue properties for membrane interaction than sequence conservation *per se*. The fact that we noted lower Ka/Ks in the BACE1 C-terminal may be related to the importance of the lipid anchoring features referred to above.

It should be noted that the Ka/Ks analysis is restricted to mammals as opposed to the deeper taxonomic levels dealt with via the larger protein sequence comparisons described in the previous section. However, the evolutionary insights they provide are complementary.

### Phylogenetic distribution of substrates

**Figure 8:**
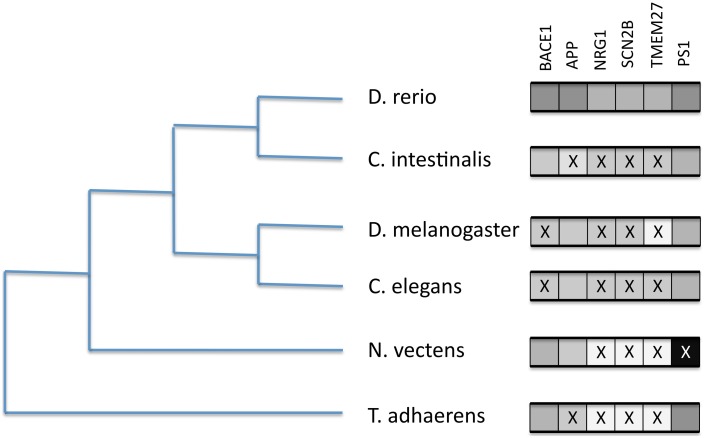
**Presence/absence of BACE1 and BACE substrates on six selected proteome sets from completed genomes**. Boxes to the right represent likely orthology matches to the human sequences for BACE1 (P56817), APP (P05067), NRG1 (Q02297), SCN2B (O60939), TMEM27 (Q9HBJ8), and PSEN1 (P49768). An X represents their probable absence by low BLASTP score.

We tested for the presence of a small number of reportedly significant BACE1 and BACE2 human substrates in model organisms and selected phylum representatives. We included presenilin 1 (PSEN1) as a highly conserved component of the gamma secretase complex as a comparison. The BLAST results (see Supplementary Data file) are summarized in Figure 8.

The figure indicates discordant evolutionary trajectories between the BACEs and the human substrates. Given the long divergence times, where BLAST scores were low (e-5–e-8) it was difficult to discriminate between short domain matches (e.g., the disulphide-rich Kunitz domain in APP) and extended but low similarity scores indicating possible orthology. However, identity and match length were useful guides. We thus conclude that APP is absent from *Trichoplax* and *Ciona*, NRG1 and SCN2B are only present in Zebrafish but TMEM27 is present only in Zebrafish and *Ciona*. In *Drosophila* and *C. elegans* we see the opposite case of APP homologs in the absence of BACE-like sequences.

### Gene expression

The consensus from many investigations is that BACE1 is predominantly a neuronal protein in humans, although glia cells may also produce significant amounts. However, systematic expression data is still sparse outside humans and mouse. We mined high-level expression patterns for BACEs using the Bgee resource (Bastian et al., 2008). While this collates expression data for human, mouse, zebrafish, *Xenopus* and *Drosophila*, coverage is patchy and difficult to standardize between ESTs and microarray probes. Expression patterns for BACE1 and BACE2 in human, mouse and *Xenopus* are compared in Table 4. Broadly, BACEs 1 and 2 showed similar expression patterns with a few exceptions. In human the only notable difference between them was that BACE2 was reported to be expressed in pancreas whereas BACE1 was not. In mouse, BACE2 was additionally expressed in the skin and prostate gland while BACE1 was not, and in the endocrine system BACE2 was restricted to the pineal gland whereas BACE1 was also expressed in the adrenal gland and neurohypophysis. In *Xenopus*, although both BACE1 and BACE2 were expressed in the testis they otherwise had distinct expression patterns: BACE1 was expressed in the brain while BACE2 was expressed in the oviduct, skin and spleen. No expression data were available for Zebrafish.

**Table 4:** **Expression of BACE1 and BACE2 in human, mouse, and Xenopus**.

**Anatomical locus**	**BACE1**			**BACE2**		
	**Human**	**Mouse**	**Xenopus**	**Human**	**Mouse**	**Xenopus**
Cardiovascular system	Y	Y		Y	Y	
Respiratory system	Y	Y		Y	Y	
Haematological system	Y			Y		
Lymphoreticular system	Y			Y		Spleen
Alimentary system	Y	Y		Y	Y	
Urogenital system	Y	Y	Testis	Y	Y	Testis Oviduct
Endocrine system	Y	Y		Y	Pineal gland only	
Musculoskeletal system	Y	Y		Y	Y	
Dermal system	Y			Y	Y	Y
Nervous system	Y	Y	Brain	Y	Y	
Pancreas	N			Y		
Sensory organ system		Y			Y	
Prostate					Y	

Presence of expression is marked by Y unless a more specific locus is mentioned. N, No expression is reported in contrast to BACE2.

## Discussion

In this paper we have mined existing complete and partial genome sequences for new BACE gene families. Blending a range of resources by manual annotation produced a rich dataset, much of it not readily available from sequence databases. This allowed us to shed new light both on the evolutionary history of the BACE genes and on putative shifts in protein function and substrate interactions. We can consequently suggest future functional characterization making use of a range of model organisms that will be relevant to development of new therapies for Alzheimer's Disease and Type 2 Diabetes Mellitus.

### Evolutionary trajectories

Our sampling of most major phyla clearly delineates an “UrBACE.” What we mean by this is the ancestral BACE-like sequence lineage with properties distinct from cathepsin-like precursors. The similarity scores and domain arrangement indicate that the emergence of this lineage was a distinct rather than gradual event and possibly related to the shuffling-in of a CTM domain into the 3′ exon. This would significantly change cellular trafficking and pH optima. We cannot detect any apparent intermediate form, such as a cathepsin with a CTM domain. By implication, these UrBACE sequences underwent selection for altered or new biochemical functions after duplication. This may have taken place before the origin of the choanoflagellates in the late Precambrian at least 860 MYA (million years ago) (Hedges et al., 2006; Blair, 2009).

Our results show that the Ur-BACE has a rich and previously undocumented history of gene duplication with preservation in different lineages. The most prominent is that leading to BACE1 and BACE2 after the divergence of the Hyperoartia (represented here by *P. marinus*) from the Gnathostomata (see Figure 3). This divergence, dated at c 530 MYA (Hedges et al., 2006; Blair, 2009), corresponds to the so-called 3R whole genome duplication (Holland et al., 1994; Miyata and Suga, 2001; Venkatesh et al., 2007; Hufton et al., 2008). This might have given rise to the paralogous and persistent BACE1 and BACE2 lineages, although phylogenetic analysis of other gene families suggests that different scenarios are possible (Kuraku et al., 2009).

Our phylogeny deviates from current taxonomic groupings but it should be noted that it has the known constitutive limitations of a gene-specific analysis. In particular, we would have expected the Cnidaria to be more basal. However, much of this deviation reflects the poor resolution of this part of the phylogeny, which contains few groups supported by high (>95%) bootstrap values. Despite this, the main taxonomic groups are mostly monophyletic except for the Protostomia. The clustering of the three *Ciona* UrBACEs away from the chordate lineage when we would have expected them to lie closest to *B. floridae* is also notable.

Gene duplication, whether or not it is associated with whole genome duplication, is a well-known process allowing the exploration of functional space by a gene family. It is thought that most duplicates are lost due to purifying selection or neutral drift soon after emergence, as they accumulate inactivating mutations (Lynch and Conery, 2000; Lynch and Force, 2000; Lynch et al., 2001). Long-term persistence of gene duplicates, as we have recorded here, is therefore *prima facie* evidence of both functional importance and differentiation in a gene family. This can be reflected in subfunctionalization (the redistribution of the ancestral gene's functions between its daughter genes) or neofunctionalization (the evolution of a new function by one of the duplicates) (Lynch and Force, 2000; Lynch et al., 2001).

It has been suggested that duplicated genes undergo accelerated evolution immediately after duplication as they adapt to new functions and undergo a concomitant increase in Ka/Ks (Lynch and Conery, 2000). Our analyses of branch lengths in the BACE1 and BACE2 families supports such an asymmetry in evolutionary rates leading to accelerated functional diversification of BACE2 with implied preservation of ancestral function in BACE1. Estimates of Ka/Ks based on complete cDNAs from a subset of mammalian species indicated higher values in BACE2 but no evidence of positive selection. This suggests relaxed purifying selection has acted on BACE2, which is consistent with functional diversification. Purifying selection on these proteins has nevertheless remained strong, implying important biological functions.

An anomaly in the scenario above is that the *X. laevis* whole genome duplication (produced by allotetraploidization circa 50 MYA) may have led to eventual loss of both BACE1s. This confounds the otherwise pan-vertebrate post-duplication persistence of the two paralogs. However, there is a caveat in that polyploid genomes are particularly difficult to assemble, so the “missing” BACE1(s) may yet be discovered in *X. laevis*. Notwithstanding, if their absence is confirmed, we make the following experimentally testable predictions. The first is an expression split in *X. laevis* if one of the BACE2s has shifted into the BACE1 neuronal role. There is a precedent for this from a study showing differential expression of 14% of *X. laevis* paralogous pairs, indicative of polyploidy-related subfunctionalization (Morin et al., 2006). We consider the alternative scenario of substitution of the BACE1 roles by another protease to be less likely, since this would need a radical functional shift to occur in *X. laevis* over a relatively short evolutionary timescale, but this is also a testable prediction.

Two loss-of-function fates can befall post-duplication enzyme copies. The first is decay to a non-translated pseudogene; the second is abrogation of catalytic function, typically due to mutations in the vicinity of the active site (i.e., the sequence is selectively maintained but for some non-proteolytic function). While neither of these can be ruled out for all individual species represented here (or their existence at a significant allelic frequency in certain populations) we suggest that, as a general case, neither of these processes has played a significant role in the evolutionary trajectory of the UrBACEs or the resulting persistence of the BACE paralogs in the vertebrates. Observations supporting this conjecture can be summarized as follows:

1. None of the 78 sequences showed frame shifts or premature stop codons characteristic of pseudogenes.
2. EST sampling or TSA virtual cDNA assembly serves as a useful proxy for protein expression (although pseudogenes can show low levels of transcription). While transcript coverage is patchy in UrBACE-containing phyla, in cases where EST numbers were high, we usually detected extensive ORF coverage. This was found to be full length for *Acropora millepora, Strongylocentrotus purpuratus*, and *Ciona intestinalis*. Even for less sampled species such as *Schmidtea mediterranea* we found ESTs covering the 5' and 3' of the cDNA (DN315134 and DN303898).
3. For vertebrates, 24 Unigene entries (i.e., clustered ESTs and mRNAs) for BACE1 and 26 for BACE2 can be found. This verifies gene transcription in a significant number of species.
4. In regard to the active site residues (equivalent to Asp93 and Asp289 in human BACE1) the proximal Asp was identifiable in 67 of 78 sequences and the distal Asp289 in 72 of 78 (regardless of whether the neighboring residues conformed to Prosite PS00141 or not). Cases where one of these was missing could be attributable to genomic assembly or gene prediction errors. Thus, pending cDNA verification for predicted ORFs, our data set contains no evidence of non-catalytic UrBACEs or BACEs.

There is a possible exception in the form of an extensive insertion in the *Schistosoma japonicum* gene sequence (Figure 4) which, if confirmed, suggests, despite the presence of two active site Asps, a severe disruption of folding. Consequent catalytic inactivity could also explain the anomalous branch length in the phylogenetic tree and could be associated with evolutionary drift into new functions for this parasite. We cannot rule out, and indeed might expect that additional ancient duplications in the UrBACE lineage could have resulted in pseudogenization of some copies (the alternative being complete deletion). However, completely finished genomes would be needed to detect such cryptic duplications.

### Substrate patterns

Our analysis of substrate co-occurrence is more of a preliminary assessment than a comprehensive analysis. It had to be limited, not only because the set of reported BACE substrates is continually expanding but also because of the scale of the analysis that would be needed to discern the evolutionary trajectory for all of them. Nevertheless, we show clear cases of phylogenetic discordance from mammalian protease/substrate pairings that could possibly have co-evolved. For example, *Drosophila* has an APP homolog but no Ur-BACE, while *Ciona* has an Ur-BACE but no APP homolog. We thus show that this type of investigation may provide an indication of significant role shifts occurring between species over long divergence times. The automated GeneTree feature in Ensembl can also be used to follow the evolution of any selected substrate (with the caveat that coverage of basal phyla is more limited than we explore here).

### Putative Ur-BACE functions

The minimum evolutionary model for the BACE gene family is that the UrBACE emerged at the base of the metazoan tree via the duplication of a cathepsin and the shuffling-in of the C-terminal TM domain. This is particularly significant because it thereby joins the limited number of proteases participating in regulated intramembrane proteolysis (RIP) of which APP beta-secretase is the archetype (Lal and Caplan, 2011; Lichtenthaler et al., 2011). Whether the Placozoans are basal to the Choanoflagellates in phylogenetic terms as urmetazoans, or vice versa, is still a subject for debate (Osigus et al., 2013). However, we detected further duplication, divergence and maintenance of the resulting paralogs in these smallest known urmetazoan gene repertoires [e.g., *Monosiga brevicollis* and *Trichoplax adherens* have only 9200 and 11,500 proteins, respectively (King et al., 2008; Srivastava et al., 2008)]. This argues for functional importance, as does their persistence for the subsequent ~800 million years. The absence of human-BACE substrates in organisms with one or more Ur-BACE sequences suggests these ancestral enzymes have different substrates and roles to those in humans. However, their loss in Ecdysozoans implies they can be functionally substituted by other proteases.

Considerations of possible ancestral functions based on bioinformatic evidence alone are speculative. Nevertheless, by extrapolating from mammalian BACE1 (which has greater similarity to Ur-BACE sequences than BACE2) we can suggest the Ur-BACEs might also have a neuronal role. While this would fit with our detection of the enzyme at the base of Bilateria (with primitive nervous systems) and the Cnidaria (with nerve nets), the counter argument would be that Placozoans and Choanoflagellates have no synaptically connected neurons. However, the surprise from the draft genomes of members of these phyla was that they not only showed extensive human protein homology but also similar domain combinations. The recent report that these phyla both express sodium channels included the suggestion that the evolution of these predates the origin of nervous systems (Liebeskind et al., 2011; Zakon, 2012). An independent observation echoes this in detecting a primordial neurosecretory apparatus in a choanoflagellate, identified via a SNARE protein complex (Burkhardt et al., 2011). Notably, *A. queenslandica* neither contains Na(v)-like channels nor an UrBACE while BACE1 regulates Na(v)1 channels in mouse neuronal cells by sequential processing of the SCN2B regulatory subunit (Kovacs et al., 2010). The question thus arises as to whether the UrBACE co-evolved for ion channel processing as one of its earliest functions after it acquired the CTM and RIP potential but this will also have to await experimental testing. These lines of evidence suggest that some of the molecular repertoire for multi-cellularity and signaling preceded the appearance of the nervous system and were later co-opted for their evolution.

The presumed functional divergence after duplication of BACEs points toward BACE1 maintaining the primary function of the UrBACE, or at least overlapping more closely with it than BACE2. However, intriguing implications arise from the recently reported functions of BACE2. One of these is the observation that pancreatic β-cells have many characteristics in common with nerve cells and may thus share with them a common evolutionary origin via gut cells co-opting a neuronal transcriptional program (Arntfield and van der Kooy, 2011). For example, while Hagfish and Lampreys have a pancreas made up almost entirely of β-cells, in *Amphioxus* these cells are associated with the intestinal tissue in a dispersed form (Pieler and Chen, 2006). An additional implication of an ancestral neuronal connection for BACE2 is that melanophore pigmentation cells are developmentally derived from the neural crest (Quigley et al., 2004). We can thus postulate that BACE2 was co-opted initially in a neuronal context (e.g., via the post-duplication gene dosage advantage) but later became selected for functions no longer confined to neural cells *per se* (i.e., neofunctionalization).

### Future work

Experimental characterization of ancestral BACE sequences, aided by the results presented, here, should have two important and related facets. The first is the purely scientific focus on biochemical and physiological roles for these enzymes in the complex degradomic protease webs that can now be compared between different phyla (auf dem Keller et al., 2007). Together with further bioinformatics analysis of basal eumetazoans, such work will reveal hitherto unknown roles, including the processing of new substrates that could have important implications for human BACE1 and BACE2. In addition, standardized comparative expression patterns across a wider variety of species will provide insight into pre- and post-gene duplication tissue specificity within and between taxa. Characterization of UrBACE(s) and putative homologous substrates may give more interpretable phenotypic and omics-profiling signatures compared to organisms with more complex gene repertoires. Some species with UrBACE sequences already have established experimental functional genomics platforms. For example, *Ciona* is used for studying chordate central nervous system regeneration (Dahlberg et al., 2009). These investigations will not only provide new testable hypotheses but also analogous putative substrate studies in *C. elegans* and *Drosophila* (UrBACE-negative organisms) could provide informative controls.

The second facet of further research relates to the crucial status of these enzymes as drug targets. This pair is unusual in being catalytically similar but the very different therapeutic indications in different tissues have been validated about a decade apart. Given the huge and increasing scale of unmet medical need for both AD and T2DM, progression toward clinical candidates for either target is to be earnestly hoped for. However, such first-in-class inhibitors face significant hurdles. Those directed against BACE1 will need (1) high dosing to cross the blood-brain barrier, (2) probably require life-long treatment, and (3) will need to be administered early to patients who are asymptomatic but with a robustly predictive biomarker profile (Karran et al., 2011). Under such circumstances, any new aspects of BACE biochemistry, implied or directly revealed by functional genomics, are going to be important. This would not only be in a predictive context for efficacy, toxicology and side effects, but also because new substrates can provide candidate biomarkers for clinical evaluation. While studies in mice and Zebrafish could be deemed potentially most informative, there is no phylogenetic grouping where experimental results could be considered irrelevant.

While BACE2 inhibition for T2DM has a longer way to go before clinical proof-of-concept, a review of recent patent publications indicates pharmaceutical company discovery programs for this indication have been running since 2010 (Southan, 2013). The major factors that might accelerate development are (1) the large collection of BACE1 inhibitor structures in PDB, (2) the collective experience in their optimization that can provide starting points for BACE2 inhibitors, and (3) unlike for BACE1, compounds need not be brain penetrant. Two years ago the numbers of compounds with inhibition results published in patents and papers was 5459 and 414 for human BACE1 and BACE2 respectively [supplementary data from (Southan et al., 2011)]. The BACE2 results were entirely from cross-screening of BACE1-directed inhibitors. The equivalent inhibitor structure ratio from published papers in ChEMBL (release 16, June 2013) is now 3815 to 527, suggesting increased interest in BACE2.

The potential importance of new insights revealed by functional genomics applies equally for both enzymes, especially since the double-target status poses important new specificity questions. In the hitherto absence of predictable *in vivo* consequences of BACE2 inhibition, the specificity ratio (i.e., the BACE1:BACE2, IC50, or Ki) for a BACE1 lead compound was probably set empirically at ~100-fold. Development projects will now need to be more circumspect on how they choose this ratio; including monitoring for effects on the turnover of BACE2 substrates and β-cell function, as well as reciprocally monitoring BACE2 inhibitors for effects on the turnover of BACE1 substrates.

Our results have additional utility for chemical biology where these pure and applied facets intersect. For example, BACE inhibitors specific for either or both paralogs could be used as system probes in the way that inhibitors have already been used for mouse and fish experiments. This is analogous to genomic loss-of-function but with the inherent advantages of small-molecule perturbation (e.g., rapid onset, precise dosing, mixture testing, analog testing, developmental stage specificity and reversal by wash-out). These can be informative when compared with activity ablation by RNAi, mutation or knockout. Such chemical perturbations could be tried out in basal phyla where we have identified Ur-BACEs with local active-site sequence similarities of 50% or above. Results are likely to be relevant to both the AD and T2DM drug discovery efforts. They could also lead to new types of BACE inhibitor cross-screens utilizing the moderate throughput phenotypic read-outs that these basal organisms can offer.

## Author contributions

Christopher Southan initiated the study, extracted sequences, analyzed data, and contributed to the writing. John M. Hancock analyzed data and contributed to the writing. Both authors have approved the final version of the manuscript.

### Conflict of interest statement

The authors declare that the research was conducted in the absence of any commercial or financial relationships that could be construed as a potential conflict of interest.
