# Supplementary material for: A tale of two drug targets: the evolutionary history of BACE1 and BACE2
Source: Front Genet. 2013 Dec 17;4:293. doi: 10.3389/fgene.2013.00293 (PMC3865767; doi:10.3389/fgene.2013.00293)
Supplement: Supplementary file 1 [file DataSheet1.DOCX]

**Supplementary Data for Southan & Hancock**

**Section 1**

Protein sequence similarity matches of reported human substrates for BACE1 and BACE2 (TMEM27 only) and the gamma secretase presenillin subunit (PSEN1) against the predicted proteomes for selected organisms. Parameters reported from the highest-scoring match are (a) the expect value, (b) the % identity and (c) the size of the best-match segment in number of residues. A diagrammatic simplification of these results is presented as Figure 8 in the paper.

| Identifier | Length | *D. rerio* | *T. adhaerens* | *C. intestinalis* | *N. vectans* | *D. melanogaster* | *C. elegans* |
| --- | --- | --- | --- | --- | --- | --- | --- |
| BACE1  (P56817) | 501 | 7.3e-66,  76%, 158 | 2e-92  49% 126 | 2.5e-74,  36%, 419 | 9e-116,  47%, 424 | 1.4e-24,  30% , 256 | 3.6e-21,  30%, 257 |
| APP  (P05067) | 770 | 2.6e-226,  62%, 439 | 3e-15  30% 73 | 1.9e-09, 3  9%, 89 | 4e-54,  26%, 603 | 1.3e-51,  39%, 167 | 1.8e-58,  39%, 163 |
| NRG1  (Q02297) | 640 | 1.2e-89,  47%, 274 | no hits | 0.0011,  21%, 129 | no hits | 1.8e-08,  34%,  89 | 0.0039,  22%, 96 |
| SCN2B  (O60939) | 215 | 2.4e-44,  51%, 172 | no hits | 0.012,  28%, 114 | no hits | 0.11,  32%, 77 | 0.86,  27%, 93 |
| TMEM27 (Q9HBJ8) | 222 | 2.9e-50  44%, 142 | no hits | 3.4e-13  32%, 129 | no hits | no hits | 0.28,  36%, 36 |
| PSEN1  (P49768) | 467 | 3.3e-134,  66%, 401 | e-159,  69%, 251 | 9.6e-105,  52% 397 | 1e-08,  84%, 26 | 1.3e-79,  43%, 401 | 1.2e-69,  50%, 280 |

**Section 2**

FASTA sequences. The short species name can be matched to the full names and Tax IDs from Table 1 in the manuscript. Any or all of the sequence strings can be copied over to a text file and then uploaded into one of the many programs for generating protein multiple alignments and tree displays. The can also be used as sequence search probes for updates that may extend the original ORF or detect new homologues.

>Schi_japo

MKTLVRLIFMYELCYIVNCYETNVINNNIEISSTLLLLSNSISSFLLYNLTGLPGQGYYM

TVYIGTPSQKIQLLVDTGSSNLAIAGRNLTNIDNWFKFNESSTLKCNNHLSQFVRYLKGY

WSGVYCQDQIDFTNKHDTDFNYFSIINNVIHDKLTISFGLIINSTKIFLQHTGSTWYGIN

GLAFPLLYIKPKSIKLFNKSIINKFIDWKIKSINQLFFNNNQLTYLDTLNSIWKIHKQFE

LLLCGTTNIHLPIHIYHQLILIIIINYVKKNSFINEIAYKHSFWTNQNAYCLNTTNDNDE

TIKRFYESFPLIEFQLVSSVNSSKQILSLLFSSQQYMRYLGRIRRNHQSKDCFAFAIQPT

HKYTILGSVFLEAYYTIFDQENMRIGFANSPCNSYTNNPSISNSKVNGLKYWNETFKSIK

KQYQKNAHLVQTHIHEPLSPLDCAVYRLTRTEQLRYFRELYLMIICLSSLIYLFLIPLLI

FLKIKYID

>Schi_mans

MTVYIGKPSQKIQLLVDTGSSNLAIAGRNLTNVDNWFKSTKSSTLRCSDHLIQHVRYLKG

YWTGVYCQDEFDFTNKQDTTIINQYSINTTNQIQLSFSLIFNSTKIFLSHIYNHSTWYGI

IGLGFTTLYIKPKLINIYHHHHYQKNLLINWKNQLINQLFDYHHHNNNQFTYIDQLNSIW

NIHKQFGLLLCGTTLFNDPDMNSRKMSGKLIIGRTDLNLLWPSTYITNNMSSSKIPSFNP

STVYYTPIRKAWYYEIILTDLLIDQYSLVDNCKELNLYKTIIDSGTTNIYLPIKIFQQLI

HYILIKYINQKSIFNHIKNKKLFWYGKNAYCLPKIINHEKSINLFYQSFPMIEFQLISSY

NTLNQVISLLLSPQQYVRYLGRINRNNQSRDCFAFAIQPTHKNTILGSVFLEAYYTIFDQ

ENMQIGFTNSPCNSYTNNPNIPLSKVNGLKQWNETFKYISSRRTYQDNINIDHLSSPLDC

AVYRPTKSVQLAYFKELHSRIFWLSTI

>Clon_sin

QSFSVIGSSPEEMNGLILIGGTNLTGMTQTVDTILYTPVREPWFYEVVLTDLRVEEQSVV

EDCKELNVEFSIVDSGTTNIQVPERVFQPLLGHIKTYVAKQSSSTAAMLQNYASFWTGAS

MLCETSSDNQIGGATGLPYTLFPVIEFQMLALEADVNKKALSLTLSPQQYIRFVGRHPKN

GVTRDCFAFGIRPHHSGTILGAVFLEGFFTVFHRDSLKVGFGNSTCNRYSNVSTVTSSMV

NGVRAWKSSIGRKTPSECAFYRPVQLRITDKLDGLIHHRLRYCRFVNWIVNLRLHWENHE

ERKILVLSMAIGRHTSMPMRRTLERLQNPGVQIASDENLTDLEYADDIVLIFEEEEKAQV

FLDELTKVIPSFVFRSYGIDFHLRNKMPVKNGKYGFLLL

>Tric_adhe_A

MAARKRTSNSIFLSLLLLQFIFLTNASYLRTLFDEIDDSNPSLIRIPLKRSNHLQSSANG

NLNNRMFKDPMTGTPGNGYVVHVWLGSPPQKLGVIVDTGSSNFAVAAAPNPAIRRFFQFQ

RSSTFVNLHRTFAVRYARGYWDGELVSDILRLGVGINGSARVQFAAIYRSGRFFVPYSNW

QGILGMAYPSIAMPPYHPVEPVFNQLVRETGIRDIFTLTLCGPIVQSQNYHLHDVDLGGM

MTFGGIDHSLFTGPIFYTPIIRKWYYQVALTGIAIGGRSLGFSCDEYNQYKTIVDSGTTN

FRVPESIFNRIIAFARSMTSVQVPNGFWEGREALCWEANNAQWNGFPYLEIALDLSDVNK

TNKQEHGQFTLMIPPQQYLRLAEHVTVHNSPCYGFGVERSQGSGIILGDVIMEGFTVMFD

RENTRVGFAASKCASNNSCDVTGTSNVSPVAIVIGSVGAAIFVIGFFMVMVVHLKVQSGR

NDMPYADPQEVQESKQLQPNSSSAYYDRPPPNPAGSAYSNSEAAGSYPYRYQAPPGNPPE

YAETE

>Tric_adhe_B

MNIVWRFILNILAFMMVAGDIPFDKIDKSVKIHKYSLYRVHKWKENNFQNAPSELSTRID

RMRGFPDIGYAIHITVGTPGQQFNALVDTGSSNFAIAASPNAGVPSYYHSRRSSTFQNRS

QQVTVQYGKGTWTGDLMSDIVHLDVGFNRAIRQDFSGTPTKTFFDQLVDKTGIANIFSLT

LYGPVLSKGLSWTSKDSNYGGEMTFGGIDRTLFLGHVYYTPVIKKWYYEVKLLNISVDGK

PITMCCHGYEKYVTFVDSGTTEIVVPPAVFRTIVHSLQKFIQAPKLFWEGREVICPAGLG

IAWSSLPIIEFTLPLAGVDGVLAYESSRFNLAISPQQYIRLSETVRNLGFPCYTFSISPS

SNDAPVIILGDALMEGYTVVFDRINNQVGFAVSRFAAKNLTVSPGINRLERVKNGSLNIQ

NISSSSEDSVAIVSVVLASISLFMTCISIGVMTCIYFNHHRNKSSNDYPYRQVSSPHHPP

AYSNAEEQYGSRDNSLADNGPTESD

>Nem_vect

MICLLDVSLTESNRKKTGIKIPLLRTKRDNNVAMENLKGRPGQGYYIASNLGSPPQRINV

LVDTGSSNFAVAASAHPYIPYYFHIDKSTSYKDLNKPVSVPYTQGSWEGELGSDLIKFVD

GPNVTLRVDVASILQSENFFINGSMWEGILGLGYARLAKPDSSVTPVFDQFVLDHGVEKD

SFSMQLCGSSEIDPSAEPTVAGTMVFGEIDEELYKGDLLYTPIVKKWYYEVVITDVAVDG

DSLALDCKKYNYDKTIVDSGTTNLRVSEEVFNAILDRLRKFDKVNLIPDDFWYGRQMMCW

NYDRTPWEEFPDMSISLLSSLSSSKEFRLKIPPQLYLRETIEHGPIMGQHCYKFAITRAE

RGTVIGAVIMEGFYVVFDRENVQVGFAATTCGGKEPMRYLLSSKFYLLNPIDCEYVETES

SDTALLTVAYVMAGVCGLCLLPIFILLGQASCKRRREQRLLNGPEYREYSDNNNSTSTP

>Aipt_pall

MRLQFNVFWVSFAVYFICLMNFTHGKKISKRKSIHRIPLIRTKRDLRDSHGVPMENLKGR

PGQGYYIATNLGKPPQRINVLVDTGSSNFAVAAAGHPYVPYYFHIEKSKTYKDLNRPVSV

PYTQGNWEGELGSDLLKFIEGPNATVRVDVASILSSENFFINGSMWEGILGLGYARLAKP

DSSVVPVFDQFVRERAVQKDSFSMQLCGSSEIEPSSEPTVAGTMVFGDIDDELYKGDMLY

TPIVKKWYYEVVITDIAVDGESLNLDCKKYNYDKTIVDSGTTNLRVSEEVFNSILDRLRK

FDRLGVPDDFWYGRQMMCWNYDKTPWEEFPDMSISLLSSISPSKEFRLKIPPQLYLRETI

EHGPLLGQHCYKFAITRAEKGTVIGAVIMEGFYVVFDRENVQVGFAATTCGAEGRLNPRS

EVSGPFSRDPVDCEYIETESSDTALLTVAYVMAGVCGLCLLPIFILLGQASCRRKKDPAS

LLSGSDYK

>Hydra_mag

INVLVDTGSSNFAVAATPHPYLPVYFHPDSQSTYFAHELRSSTFTDLNRAVAVPYTQGNW

EGKLGSDLIRFIDAPNATVRVNIAAIHSSENFFINGSNWEGILGLGYARLSKPDSSVTPL

FDQFVISDVVEDTFSMQLCSTSELDPSSEPTVAGTMVFGEMDDDLYKGDMLYTPIVKKWY

YEVVITDMSVNGISLGVHCKKYNYDKTIVDSGTTNLRVPEEIFNLILTQLKKYDKVTLIP

DEFWIGRQMMCWNYDKTPWEEFPHMSISLLSSLSSSKEFVLKIPPQLYLRETIERGPLPG

QHCYKFAITKAEKGTVIGAVVMEGFYVVFDRENIRVGFAATTCGAEGRLNPRSEVSGPFI

RADPTDCEYIESELADTALLTVAYVMAGVCGLCLLPVFVLLGQASCKKSVEK

>Clytia_hem

MIKPDGVITRSLYVLVLTIMFLISTCSCGGSKKFPLMKRRNQATKDVVTTENLKGRPGQG

YYIAILFGTPPQRINVLVDTGSSNFAIASRPHPYLPLYFHADQSKTFVPLNRPVAVPYTQ

GNWQGELATDFIQFIDALNVTVNVNVALIKSSENFFINGSNWEGILGLGYTHLSKPDSSV

IPVFDQFVIDNVVDDTFSMQLCSSSELDPSAEPTVAGTLVFGETDEDLYKGDLFYTPIVK

KWYYEVIITDFAVNGVSLGLNCKKYNYDKTIVDSGTTNLRVPEDVFNAILVHLKKYDRLG

VPDEFWIGRQMMCWNYDKTPWEEFPDMSISLLSSVSSSKEFILKITPQLYLRETIERGPL

PGQHCYKFAITKAERGTVIGAVIMEGFYVVFDRENVQVGFAATTCGAEGRLNPRSSVSGP

FNRADPVDCEYIETEPADSALLTVAYVMAGVCGLCLLPVFILLEQASCKPKLVK

>Acro_mill

TSYKDLNRPVAVPYTQGNWEGELGSDLLRFVEGPNATVRVNVAAILSSENFYINGSMWEG

ILGLGYTRLSKPDSSVLPVFDQFVNDGVVKDSFSMQLCGSSEIDFSSEPTVAGTMVFGEV

DTDLYEGDLLYTPVVKKWYYEVVITDIAVGGESLNLDCKKYNYDKTIVDSGTTNLRVSEE

VFDGILDRLKKFDKLGVPDDFWIGRQMMCWNYDKTPWEEFPDMsisllssisnsKEFRLK

IPPQLYLRETIEHGPLLGQHCYKFAITKADKGTVIGAVIMEGFYVVFDRENVQVGFAATT

CGAEGRLNPSSEVTGPFSRDAVDCEYIETESSDTALLTVAYVMAGVCGLCLVPIFVLLAQ

ASLKR

>Mere_mere

MPSKNCEWPRQILTTFFLTNVILTTVLGNVLHVNRLKRLAKPSETGEIAQRDIQYGQQIG

NLMGKPGEGYYLQVEIGTPSQKLNVLIDTGSSNLAVACKPQKEIQSHFEPEKSSTYKKLG

RSVNVPYTIGSWSGDLGEDEIRMSTFSDSALRANIACINQNTVGGSLIFGGTSSDLYTGE

LFSTPINKEWYYEVIIVDIQVAGSSLNMDCKEYNFGKTIVDTGTTNLRLPYRVFNKLVQY

LKQDLQSNPSVTRIPTNAFWSGDEVLCWQAGQVPYHVFPTVSLVLPESDTAAFKLVISSQ

QYLRPVGEENDDSPENDCYKFGISSLDSGSVIGAIVMEGFYIVFDRENKQVQFANSTCGV

LD

>Rudi_phil

YGSQIGNLLGRPGEGYYLQVEIGTPPQKFNVLIDTGSSNLAVACKPSIEINTHFQTDKST

TYKELGKSVTVPYTLGSWDGELGTDVIRITSIPNVNITANIACINKAEHFFINNSNWQGI

LGLGYAEIARPDSSVEPLFDTMVHDRQSSNIFSVQLCGLGYFSPNPTNKVGGSLIFGSTS

PELYDG

>Vill_lien

AEIARPDSSITPFFDSLVHQTGIPNMFSMQLCGPVSNSNSSDIQMGGTMILGGLDSSLLH

IGPIFYTPIYKTWYYEVILLDVQVGGVSLNMDCKEYNFGKTIVDSGTTNLRLPVKVFSSI

VTRVQQSLLHTNLTIQLTSFWTGDEVLCWGRNKVPYNDFPVITLALPATTDSMFSLNLSP

QQYLRPVNDDSNTTKGEDCFKFAITSSESGTVIGAIVMEGYYVIFDRQNLQVGFAESVCS

VRDDRARKSNVQGLQKYTGNYKDCAYVKVETRDNTLNIVAYVMAGICGLCVLPLFVIFAQ

WQLRKCFCRRKKLERSNSDLTDLVSD

>Ilya_obso

KQAREEGVTQQMEGISGEGYFLNISVGTPPQQMQILVDTGSSNTALAASPNPHITQYFNR

SLSSTYYDSGETIHVPYTQGEWNGDLGSDLVALTSLPGVEFRANMAFITQSQGFFIPDSN

WQGIVGLAYQSIARPETLTPFWDSLRESSNLSDVFSMQLCGAAHSHNASAPGPGMQGNLV

LGGVSVDLYTGDIFYTPIIKEMYYEVVITDMAVQGKSLHLDCKEYNFAQTIVDSGTTHLR

LPTRVFDEVV

>Stron_purp

MHFSLPTSRIVVVVPAAAICIVCVLIETCTAARSHVYTIPLRKGKETSFAETVGEPVRTN

QVNVSVEEQKNNIRGRPGLGYYIEVDIGTPPQKLNVLIDTGSSNFAVAASSHNAISTYYR

RNESSTYEDQGTYVKVPYTQGEWSGDLGQDLVQIASLGNQSFQANIAAITESKMFFLNDS

RWQGILGLGYAEIARPDSSVEPFFDSLTSQTSIQDIFALQMCGALASTNDTNLGSSADGP

VEEVIGSMNIGGLDASLYHGTMQYAPLRDEWFYEVIMTDIRVGNDSLGLDCKEYNFDKTI

VDSGTTNLRLPVRVFEAITNAIKAHTTKHMPDVPSEFWTGMNLMCPTDSTSPYEPYHWFP

TLTLDLQSTNQGQAFSLVVSPQQYLRRDYDHEDKKNCFKFAIAPSTNHAVIGAVIMEGFY

VVFDRENKRVGFARSTCPGACEKTGTCVGNSPLITEAFNIDFDASDCGYDRSTSYDPALT

ITAYVLAAICLVCLIPVIVFALTHQINKRCKGRRGRGVVNHHRLDQEGLAENEPNSDP

>Sacc_kowa

MGGTMTVGGIDEELYTGSIFYSHIRKQWYYEVVITDMEVDGKSLLEMDCKEYNFDKTIVD

SGTTNLRVPLKVFNRIVKGIDSTINVTITPGFWTGAELMCYGEDVTPWQIFPSFSISLLS

ENLSQVYKLIISPKQYMRKVDDVSNTINDCYKFAITTSASGTVIGAVIMEGFYVIFDREN

QRVGFAASSCAADDSTISPQVLGPFSYDNSSDCAYHIPENNEATLMVVAYVMASICGICM

VPLFIMVMQCYQKKSIEKKNDGETLIDD

>Mono_ovat_A

MRVLLLIALAAVAAAWRVELQRVTATETHESPAWRRTLYPSAPLKGIPATAYQLTMAFGT

PPQNFSIIVDTGSSNLAVAGSAAAGASSYFVTAQSSTFSNTQTPVSVSYMIGAWSGTIVS

DKVEMVGSGTAPQTAQFALITASQSFFVSNSPYPAQGIIGLGYRSLAFPAANPITPFFDT

LVASQGVPNAFALQLCEALVVHTGGDIVATSVTSYMEVGATESSVSDLSRGTWFYTPIVE

ELYYGVTVVGMAVNGTRLDVDCSLYNSPRAAYDAIVAILSTKITFASSSDQSKFFSGQEC

FLLTPAFIETLPSVTVTLAGHNGTEFDLTVQPSHYLIAQSVPASAVTNPSVLVGRDCRSF

GIQPTCGTIIGIAAMAPYTVIFDRAGQRIGFAVSTCSNSSGYSAVPSIASRASSATCSGS

GRCGSSSSTSTNTAAIIGGVVGGTGAVAVLGLLAWKFLA

>Mono_ovat_B

MAVSQVLAIVMALACVSEAIVSIPVHRHRVSRRIARDEVTMLSGSSYTAPIPPDSYMIYV

SIGTPPQQLKLLVDTGSSNLAVSISAQAGGPPPYFQAASSSTYQLVAANALDMEYVVGDW

RGDIVQDIVTVENAPNISLDVSFGTITSSSEFFTEDCAYNGILGMAYSAIAQPQSHPIPP

YFDQLVSANLVPNAFALQLCMPENAAVDNGKMVLGGYDPSLSYGTPVYTPLMQQGYYSVL

VTDFAVNGASLGLACGGAYNSPSYSIVDSGTTELLVPQAVFNALIGAIPEGLRSYFSDQN

NVVYVPPAACDVSQPSVNCLGGWPTLTTYLAGTAAGTEFPLTIRPWHYMRVVQDQFGAWT

FQSAIVPACNEGSGITLGVVAMTEYQVVFDRANSRLGFAPSTCAQSSQLGPPPAIAIQAR

VDQSSCEAEKVSCSPPGQPSIFMIYFWVAVICGGLAVILVIGSVVWCFVSKGPRTRSRYE

MMQNVDMNSSPRGYDTGRSDGGYYMHDGMDDDLPDDNSPPAFDGADSRPLPFRLGWARTP

V

>Lott_giga

MKVGYNMRGLVHQSFILGLVLSLLLSLCHGLTYHIPLKRSVNHDRHESHRDRVTRDVNTN

EKLFGHSGNGYYMTMLIGTPPQKINVLIDTGSSNLAIAAVPDINIDKHFVRINSSTYEED

GREVQVPYTQGEWKGLLGTDDVQLTVLPNITVRPNIAFITQSQNFFINGSEWQGIVGMAY

ESIARPDSSIVPFFNSLVKDGGVRDIFAVQLCGTPYLAEDDKMGGSMTIGGLSSDLYNSG

IYYTPLQKLWYYEVLITDIAVDSNSLGLDCKEYNFDRTIVDTGTTNMRFPKRVYKSILNH

IQHKVQIMERPFSPSFWSGDEVMCWEDAMLPFQVFPTISLSMYHSENSLFKLHIPSEKYL

RMVIDPFETGKCFKFGIASSEAGTVLGAILMEGYYVVFDRENKRVGFATSPCNAVYNNHS

KPAIEGYINSTENLKDCYYIKPASDQSSMLIVAYVMAAICCICVLPLIIMIIQWQYRRSC

GNHSNDEEDDGNLIE

>Petr_mari

MLDNLRGKPGQGYYIEMALGTPPQMLNILVDTGSSNFAVGAAPHLFLKRYYRPERSSTYR

PSHSSVYVPYTQGNWQGDLGTDLVSVPHGGNSSVRANVAAITYSNSFFINGSNWEGILGL

AYANIARPDDSLEPFFDSLVKQTGIPNVFSLQLCGLFPDPNSTDGTAGGTMIVGGVDTDL

YIGDMWYSPIRREWYYEVIIVRMEVHGRDLKMDCKETEKFPEGFWLGEQLVCWRPGSTPW

HLFPNISLYLMGEATNQSFRVTIMPPQQYLRAVEDVASMLDDCYKFAISQSSTGTVIGAV

VMEGFYVVFDRAHKRVGFAVSRCSVRGPGDSPSVSGPFVLPDVNMAECAYSAARPDETAL

LTVAYVMAAICALFMLPVCLLTLQGQCGRCRRRAGDELTDDTSLLK

>Bran_flor

MCGFSALASSLLVLLWTSVCVSPARLLGDDGKVLRIPLKRISREIREDVLERNVGADPST

QEDNLRGKPGQGYYVQMALGTPPQLLNILVDTGSSNFAVGAAPHPFLDKYFDRSLSSTYT

DTQKSVYVPYTQGNWKGSLGTDEVSLPSGPNVTVRANIASITSSSNFFINGSHWEGILGL

AYSEIARPDSTVEPFFDSMVKEGRVSNIFSMQLCGTIDQGNTTDISVGGTMVVGGIDADL

YEGPILYSSLRREWYYEVVITKVEVDGEDLGMDCKEYNFDKTIVDSGTTNLRVPKKVFRK

VKQMLDAKTDIDIPAEFWTGEDLMCWKIGSTPWEHFPPMGIYLQGTSNSEAFRLSISPQQ

YMRAVSDGLGRTEDCYKFAITSSDTGTVIGAVVMEGFYVVFDRENKTVGFAKSTCGVRDT

TQSSGVAGPFPHSNTTDCAYTVPQEDTTVLMMVAYVMAGICGMCMLLLVVMLGQWQNNTE

CFSGGSYKVSVLF

>Cion_inte

MEFNFGNLLFMAVLLCMGDAKIRIPLRYKRASANNLKGTTVTGYYITVNLGTPPQTFNVI

VDTGSSNFAVSGTKTSFSDSFYNKALSNTSIDTTLPTVDVHYTEGWWKGNIVSDLLTIPS

ANLIQPVRVHVADISETKNFFINESQWIGILGLAYSSLVLPKNGGLWSVMHDIVEQTTLE

DILSMQLCSTPTRDETQGVLLFGEWDSSLATGQIYWTRIVKQWYYDILITGLKVGDTMIE

VDCEELNNDRTIVDSGTTNLRLPQKVYNLVVSVIKTKLVKDDFDDDFYDGTKMFCTSEPN

VLFSWFPNLSIFLPSTEANKTIELTVFPQSYLQLTEGLVTETLLGRSCYKFAIFPSTSGS

VLGTVVMEQYYVVFDRGNNTVGFATSKCAVLPTTSIQIRNGWINSTSCQYISHSVKSPLT

LAAYIVLGLFCICLLPVCFLIAYNFFRKRSPGIGCCSGSYNQLNDT

>Cion_savi

MVKFLWCLWLGVFIVCSGARIRLKRAATDNLHGTTITGYYVTVKLGNPPQELNVIVDTGS

SNFAVAGGPNPVISNFYNKSSSNTSVDTGVRSVKVDYTEGWWMGDVVSDVLSVPSAQLDT

SVRVPVADITNSNKFFVNGSNWVGILGLAYSDLVLPKGNGLKSVMHEITHQTSTPDLLSM

QLCSTSLQDATYGALLIGEIDLSLAAGPLYWTPIVKQWYYDIIVSGLKIGDKVVDIDCSD

INYDRTIVDSGTTNLRFPQKVYDIILPIIKASVVRFYFDQDFYDGKTMFCTTDPAELYGE

FPNITIYLPSTNENQTIELTIHSASIPQFTTMLDASKQSCYKFAIFPSAIGTVLGTVLME

EYYVVFNRINSSIGFALSACHKGSTIQVINKWINSTNCRYIPPTKPITYAAYVVLALFCI

CLLPLCFLLAFNYFRSKSTPCRPRESYDQLDDHHEVSH

>Halo_rore

MKLVSSLLLMYWLINQGQFQFGLCSLTYEIKSKRAIVGSAIENGNLHGLIEKGYYIEVLM

GTPPQRLNILIDTGSSNFAVASFNSSLTRRYYDSEKSSSVINSGIPDVDIEYTEGFWKGP

LVTDLVSIPEAGLTEQVRVDIVKITSSKKFFINGSGWQGIIGLGYDELVRPNNPKVKSFM

TSVIENTSVRNVFSIQLCAANTMNFSDVTTGSLVFGDYDRTDGTIFRTRIVHEWYYEVIV

LGMKVGNSLVTVPCREFNNDKSIVDSGTTNLHLPEKVFNSVIDLMKKITAKTIEDFVIDD

AFWDGLTVLCWDPDFNPYLFFPNLTVYLPSVEINNTISLMLSSYHYILYNKNLTNEDADN

TRCFKFGISSSQTGTVLGAVLMEQYVVVFDRENRTVGFTRSNCSEYSELEHYGGYINSTA

CTYDANTGLSSLQIATYVILGLLGLCAMPACILFYIWCYKSSR

>Eupr_scolo

HAIVWTSSPSTSDDSYQTGGTLVLGNIDETMYQEPIYYTPIQKTSYYEVLIADMLVNGTS

LDLDCKEYNFDKTIVDSGTTNLRLPYTVFQAVVDKLKQDVLVVNQRPPDIFWKGEDMLCW

ENSDVPFRSFPPITIALVTGNNSVFHLVISPHQYLRAVGDASNSMLNNGDCFKFAIAGSD

SGTVIGAVVMEGYYIIFDRANKSIGFAETTCHAHNVQTIKSKIEGPFPSKVNTSTCLFQT

MEPDDSSLLDVAYVLAGICALCAIPLVVLVFQWQKKILDFKERKR

>Plan_schm

MIKQIQIYIFIFGILYSSGEIHSPLFRMIPPQNIYPEESDFSLKGFNVAEKSVDNRIITL

IETDLKGISNYGYYMTVGIGSPPQKFNLVIDTGSSNTAVAGSPQQFLSNYFKSQLSSTYV

DHKISVGVPYDSGKWNGLYSSDIFTFEGVAYIDANKTQRYSKEINVRSNFGLIKESTTFF

PKNGNFQGILGLAYPVLAQPANLIHKSTLFDDVVMKYGLPNMFSIVLCGHNRNESQPTTN

GFFIAGGKNDSLYNPPLVDILIRRKWFYEIIISRISISNKPIILSSCKEYNRDKSILDTG

TADTYFPNVIFHSLVSSIFNMKNAPFKVTIHSPFWDRRAVICILSNSPNHPSTYFPEIQI

EFVINDTSSLEVALSSQQYIQYVGDQIDSFGNRRNCYKFGFGVSKSGTILGDSFLEGFYF

LFNKELSKVGIAQSKCNLHVATVSRSRIIKVNRGQSGLLACAVRAGSKKSFLSQLTPTLT

IIIGVVFGVILIPSLVLMVYYRCAETKDPEQAALIQ

>Hum_BACE1

MAQALPWLLLWMGAGVLPAHGTQHGIRLPLRSGLGGAPLGLRLPRETDEEPEEPGRRGSF

VEMVDNLRGKSGQGYYVEMTVGSPPQTLNILVDTGSSNFAVGAAPHPFLHRYYQRQLSST

YRDLRKGVYVPYTQGKWEGELGTDLVSIPHGPNVTVRANIAAITESDKFFINGSNWEGIL

GLAYAEIARPDDSLEPFFDSLVKQTHVPNLFSLQLCGAGFPLNQSEVLASVGGSMIIGGI

DHSLYTGSLWYTPIRREWYYEVIIVRVEINGQDLKMDCKEYNYDKSIVDSGTTNLRLPKK

VFEAAVKSIKAASSTEKFPDGFWLGEQLVCWQAGTTPWNIFPVISLYLMGEVTNQSFRIT

ILPQQYLRPVEDVATSQDDCYKFAISQSSTGTVMGAVIMEGFYVVFDRARKRIGFAVSAC

HVHDEFRTAAVEGPFVTLDMEDCGYNIPQTDESTLMTIAYVMAAICALFMLPLCLMVCQW

RCLRCLRQQHDDFADDISLLK

>Mouse_BACE1

MAPALHWLLLWVGSGMLPAQGTHLGIRLPLRSGLAGPPLGLRLPRETDEESEEPGRRGSF

VEMVDNLRGKSGQGYYVEMTVGSPPQTLNILVDTGSSNFAVGAAPHPFLHRYYQRQLSST

YRDLRKGVYVPYTQGKWEGELGTDLVSIPHGPNVTVRANIAAITESDKFFINGSNWEGIL

GLAYAEIARPDDSLEPFFDSLVKQTHIPNIFSLQLCGAGFPLNQTEALASVGGSMIIGGI

DHSLYTGSLWYTPIRREWYYEVIIVRVEINGQDLKMDCKEYNYDKSIVDSGTTNLRLPKK

VFEAAVKSIKAASSTEKFPDGFWLGEQLVCWQAGTTPWNIFPVISLYLMGEVTNQSFRIT

ILPQQYLRPVEDVATSQDDCYKFAVSQSSTGTVMGAVIMEGFYVVFDRARKRIGFAVSAC

HVHDEFRTAAVEGPFVTADMEDCGYNIPQTDESTLMTIAYVMAAICALFMLPLCLMVCQW

RCLRCLRHQHDDFADDISLLK

>Rat_BACE1

MAPALRWLLLWVGSGMLPAQGTHLGIRLPLRSGLAGPPLGLRLPRETDEEPEEPGRRGSF

VEMVDNLRGKSGQGYYVEMTVGSPPQTLNILVDTGSSNFAVGAAPHPFLHRYYQRQLSST

YRDLRKSVYVPYTQGKWEGELGTDLVSIPHGPNVTVRANIAAITESDKFFINGSNWEGIL

GLAYAEIARPDDSLEPFFDSLVKQTHIPNIFSLQLCGAGFPLNQTEALASVGGSMIIGGI

DHSLYTGSLWYTPIRREWYYEVIIVRVEINGQDLKMDCKEYNYDKSIVDSGTTNLRLPKK

VFEAAVKSIKAASSTEKFPDGFWLGEQLVCWQAGTTPWNIFPVISLYLMGEVTNQSFRIT

ILPQQYLRPVEDVATSQDDCYKFAVSQSSTGTVMGAVIMEGFYVVFDRARKRIGFAVSAC

HVHDEFRTAAVEGPFVTADMEDCGYNIPQTDESTLMTIAYVMAAICALFMLPLCLMVCQW

RCLRCLRHQHDDFADDISLLK

>Dog_BACE1

MARALPWLLLWMGSGVLPARCTPPGIRLPLRSGLGAPPLGLRLPRETVEEPDEPGRRGSF

VEMVDNLRGKSGQGYYVEMTVGSPPQTLNILVDTGSSNFAVGAAPHPFLHRYYQRQLSST

YRDLRKGVYVPYTQGKWEGELGTDLVSIPHGPNVTVRANIAAITESDKFFINGSNWEGIL

GLAYAEIARPDDSLEPFFDSLVKQTHVPNLFSLQLCGAGFPLNQSEVLASVGGSMIIGGI

DHSLYTGSLWYTPIRREWYYEVIIVRVEINGQDLKMDCKEYNYDKSIVDSGTTNLRLPKK

VFEAAVKSIKAASSTEKFPDGFWLGEQLVCWQAGTTPWNIFPVISLYLMGEVTNQSFRIT

ILPQQYLRPVEDVATSQDDCYKFAISQSSTGTVMGAVIMEGFYVVFDRARKRIGFAVSAC

HVHDEFRTAAVEGPFITLDMEDCGYNIPQTDESTLMTIAYVMAAICALFMLPLCLMVCQW

RCLRCLRHQHDDFADDISLLK

>Bov_BACE1

MAQALPWLLLWMGSGVLPAHGSQPGIRLPLRSGLGGAPLGLRLPRETDEESEEPGRRGSF

VEMVDNLRGKSGQGYYVEMTLGSPPQTLNILVDTGSSNFAVGAAPHPFLHRYYQRQLSST

YRDLRKGVYVPYTQGKWEGELGTDLVSIPHGPNVTVRANIAAITESDKFFINGSNWEGIL

GLAYAEIARPDDSLEPFFDSLVKQTHVPNLFSLQLCGAGFPLNQSEALASVGGSMIIGGI

DHSLYMGSLWYTPIRREWYYEVIIVRVEINGQDLKMDCKEYNYDKSIVDSGTTNLRLPKK

VFEAAVKSIKAASSTEKFPDGFWLGEQLVCWQAGTTPWNIFPVISLYLMGEVTNQSFRIT

ILPQQYLRPVEDVATSQDDCYKFAISQSSTGTVMGAVIMEGFYVVFDRARKRIGFAVSAC

HVHDEFRTAAVEGPFVTPDMEDCGYNIPQTDESTLMTIAYVMAAICALFMLPLCLMVCQW

RCLRCLRHQHDDFADDISLLK

>Mono_dome_BACE1

MAQALPWLLLWMSSGVLCTQPGIRLPLKSGLPLGLRLPREAIEELDEPSRLLSHRSSFVE

MVDNLRGKSGQGYYVEMTVGSPPQTLNILVDTGSSNFAVGAAPHPFLHRYYRRQLSSTYR

DLRKGVYVPYTQGKWEGELGTDLVSIPHGPNVTVRANIAAITESDKFFINGSNWEGILGL

AYAEIARPDDSLEPFFDSLVKQTQVPNLFSLQLCGTGFPNLNQSEALASVGGSMIIGGID

HSLYVGSLWYTPIRREWYYEVVIVRVEINGQDLKMDCKETEKFPDGFWLGEQLVCWQAGT

TPWNIFPVISLYLMGEVTNQSFRITILPQQYLRPVEDVATSQDDCYKFAISQSSTGTVMG

AVIMEGFYVVFDRAHKRIGFAVSACHVHDEFRTAAVEGPFVITDMEDCGYNIPQTDESTL

MTIAYVMAAICALFMLPLCLMVCQWRCLRCLRRQQDDFADDISLLK

>Ailu_melan_BACE1

MAQALPWLLLWMSLGVLPAHGTQPGIRLPLRSGLGAPPLGLRLPRETVEEPEEPGRRGSF

VEMVDNLRGKSGQGYYVEMTVGSPPQTLNILVDTGSSNFAVGAAPHPFLHRYYQRQLSST

YRDLRKGVYVPYTQGKWEGELGTDLVSIPHGPNVTVRANIAAITESDKFFINGSNWEGIL

GLAYAEIARPDDSLEPFFDSLVKQTHVPNLFSLQLCGAGFPLNQSEVLASVGGSMIIGGI

DHSLYTGSLWYTPIRREWYYEVIIVRVEINGQDLKMDCKEYNYDKSIVDSGTTNLRLPKK

VFEAAVKSIKAASSTEKFPDGFWLGEQLVCWQAGTTPWNIFPVISLYLMGEVTNQSFRIT

ILPQQYLRPVEDVATSQDDCYKFAISQSSTGTVMGAVIMEGFYVVFDRARKRIGFAVSAC

HVHDEFRTAAVEGPFITPDMEDCGYNIPQTDESTLMTIAYVMAAICALFMLPLCLMVCQW

RCLRCLRHQHDDFADDISLLK

>Orin_anat_BACE1

MARALPWLLLWAVLPARGAHPGIQVPLKTRQGSFVEMVDNLRGKSGQGYYVEMTVGSPPQ

TLNILVDTGSSNFAVGAAPHPFLRRYYRRQLSSTYRDLRKGVYVPYTQGKWEGELGSDLV

AVPHGPNVTVRANIAAITESDKFFINGSNWEGILGLAYAEIARPDDSLEPFFDSLVKQTS

VPNLFSLQLCGAGFSLNETEAPASVGGSMIIGGIDPSLYVGGLWYTPIRREWYYEVIIVR

MEINGQDLKLDCKEYNYDKSIVDSGTTNLRLPKKVFAAAVKSIKTASSTEKFPDGFWLGE

QLVCWQAGTTPWDIFPVISLYLMGEVTNQSFRITILPQQYLRPVEDVATSQDDCYKFAIS

QSSTGTVMGAVIMEGFYVVFDRARKRIGFAVSTCHVHDEFRMAAVEGPFVTPDTEDCGYN

IPQTDESTLMTIAYVMAAICALFMLPLCLMVCQWRCLRCLRRPWDDFADDISLLK

>Gall_Gal_BACE1

MTMGSPPQKLNILVDTGSSNFAVGAAPHPFLRRYYQRQLSSTYRDLRKGVYVPYTQGKWE

GELGTDLVTIPHGPNVTVRANIAAITESDKFFINGSNWEGILGLAYAEIARPDDSLEPFF

DSLVKQTQVPNIFSLQLCGAGFSPNETETLASVGGSMIIGGIDRSLYVGDIWYTPIRKEW

YYEVIIVKLEVNGQDLNMDCKEYNYDKSIVDSGTTNLRLPKKVFEAAVKSIKTASSTEKF

PDGFWLGEQLVCWQVGTTPWHIFPVLSLYLMGEATNQSFRITILPQQYLRPVEDVATSQD

DCYKFAISQSSTGTVMGAVIMEGFYVVFDRARKRIGFAVSACHVHDEFRTAAVEGPYLHS

NMEDCGYNIPQTDESTLMTIAYVMAAICALFMLPLCLMVFQWRCFRCLRRDHDDFADDIS

LLK

>Taen_gutt_BACE1

PRGRPPRRGAGRCGPADRVPRGLPAAMAPAWPWLLLWLGALRALPAPPRIRLPLRAGAAP

PSGLRQRRAPLGAEPDSAGSFLEMIDNLRGKSGQGYYVEMTVGSPPQKLNILVDTGSSNF

AVGAAPHPFLRRYYQRQLSSTYRDLRKGVYVPYTQGKWEGELGTDLVTIPHGPNVTVRAN

IAAITESDKFFINGSNWEGILGLAYAEIARPDDSLEPFFDSLVKQTRVPNIFSLQLCGTG

FSPNETEAVASVGGSMIIGGIDRSLYVGDIWYTPIRKEWYYEVIIVKLEVNGQDLNMDCK

EYNYDKSIVDSGTTNLRLPKKVFEAAVKSIKTASSTEKFPDGFWLGEQLVCWQVGTTPWH

IFPVLSLYLMGEATNQSFRITILPQQYLRPVEDVATSQDDCYKFAISQSSTGTVMGAVIM

EGFYVVFDRARKRIGFAVSACHVHDEFRTAAVDGPHPHSNMEDCGYNIPQTDESTLMTIA

YVMAAICALFMLPLCLMVFQWRCFRCLRRDHDDFADDISLLK

>Xen_trop_BACE1

MELVVYARTGSILLVLLPCFLPVTGQVAIKLPLKNCPGTCSPMAYRAKRSAEKGMELDTN

FLDMIDNLRGKSGQGYYVEMRVGTPPQTLNILVDTGSSNFAVGAAPHPFLKRYYHRQHSR

TYRDVRRGVYVPYTQGKWEGELGTDLVTIPHGPNVTVTANIAAITDSDKFFINGSNWEGI

LGLAYAEIARPDDTLEPFFDSLVKQTRVPNIFSLQLCGTGFHFNESDTSASVGGSMVIGG

IDPSLYIGSIWYTPIRKEWYYEVIIVKIEINGQDLHMDCKEYNYDKSIVDSGTTNLRLPK

RVFDAAVKSIKAASSTEKFPDGFWLGEQLVCWQEGTTPWHIFPVISLYLMGEVANQSFKI

TILPQQYLRPVEDIATAQEDCYKFAVSQSTTGTVMGAVIMEGFYVVFDRANKRIGFAVST

CHVHDYFRTASVEGPFFASDLEDCGYNTPQLDESALMTTAYVMAGVCALFMLPLCLMLFQ

WRCFRCLKRARDDLADDISLLK

>Anol_caro_BACE1

SQPDDSLEPFFDSLVKQTRVPNVFSLQLCGAGFLPNDTEALASVGGSMIIGGLDPTLFRG

SVWYTPIRKEWYYEVIIVKIEINGQDLQMDCKEYNYDKSIVDSGTTNLRLPKKVFEAAVK

SIKTASSTEKFPDGFWLGEQLVCWQVGTTPWHIFPVISLYLMGEATNQSFRISILPQQYL

RPVEDVATSQDECYKFAISQSSTGTVMGAVIMEGFYVVFDRARKRIGFAVSTCHVHDEFR

TVAVEGPFPQSNMEDCGYNIPQTDESTLMTIAYVMAAICALFMLPLCLMVSQWRCLRCLQ

RGRDDFADDISLLK

>Dani_reri_BACE1

MRAARLLLLCGVAVCVARLCASSGPALLRVPLRQGPLTPHSTPAAHRSPHGPRARSRRAA

SGISFINMIDNLRGKSGQGYYIEMAVGSPAQRLNILVDTGSSNFAVGAAAHPFLHRYYHR

SLSSSYRDLGRGVYVPYTQGRWEGELGTDVVSVPCGPNVSLRANIAAITQSDRFFINGSN

WEGILGLAYAEIARPDETLEPFFDSLLRQSTVADVFSLQLCGAGYNHNYSTGSSSTVGGS

MIIGGVDPSLYVGELWYTPIRREWYYEVIIVRIEVNGQDLNMDCKEYNYDKSIVDSGTTN

LRLPRKVFQAAVKAIEAASSTEQFPSGFWLGEQLVCWQAGTTPWHIFPVISLYLMSENRN

QSFRISILPQQYLRPVEDVASAQEDCYKFAVSQSSTGTVMGAVIMEGFYVVFERQHKRIG

FAVSTCHVHDEFRTAAVEGPFHGLDLEDCGYNVPQTDESTLMTIAYIMAGICALFMLPLC

VMVCQWRFTCCLQPADGSDDISLLK

>Taki_rubr_BACE1

MIDNLRGKSGQGYYVEMAVGSPPQKLNILVDTGSSNFAVGAAAHPYLRRYYHRSLSSSYH

DQGKSVYVPYTQGRWEGELGTDLVSVPHGPNATLRANIAAIIQSDRFFINGSNWEGILGL

AYADIARPDETLEPFFDSLVRQTSVPNIFSLQLCGAGFTQNYSLGSATVGGSMIIGGVDP

SLYVGEIWYTPIRREWYYEVIIVRIEVNGQDLNMDCKEYNYDKSIVDSGTTNLRLPKKVF

QAAVKAIEAASSTEQFPSGFWLGEQLVCWQAGTTPWHIFPVISLYLMSENHNQSFRISIL

PQQYLRPVEDVASAQEDCYKFAVSQSSTGTVMGAVIMEGFYVVFDRENKRIGFAVSMCHV

HDEFRTASVKGPFHGVDLEDCGYNIPQTDESMLMTIAYIMAGICALFMMPLCLMVCQWRF

ARCLHPHGDLADDISLLK

>Tetr_nigr_BACE1

MRTGTSVLFPSLKLASWPSRRIRITGSPSLKSPATHLLKASGLPMARTAPAPTMTRVRVF

MSRTPRVAGRESWALTWCLCPTVPTPHMRANIAAIIQSDRFFINGSNWEGILGLAYADIA

RPDETLEPFFDSLVRQTSVPNIFSLQLCGAGFTQNYSLGSATVGGSMIIGGVDPSLYVGE

IWYTPIRREWYYEVIIVRIEVNGQDLNMDCKEYNYDKSIVDSGTTNLRLPKKVFQAAVKA

IEAASSVSLDNVGHLKMEKTEKTEQFPSGFWLGEQLVCWQAGTTPWHIFPVISLYLMSEN

HNQSFRISILPQQYLRPVEDVASAQEDCYKFAVSQSSTGTVMGAVIMEGFYVVFDRENKR

IGFAVSTCHVHDEFRTASVKGPFHGGDLEDCGYNIPQTDESMLMTIAYIMAGICALFMLP

LCLMVCQWRFARCLHPHGDLADDISLLK

>Gast_acul_BACE1

LLLCTSACLLGGGRSAPDASGLPLAIRVPLRQGARPSRRRRGAAARSVSFVDMVDNLRGK

SGQGYYVEMAVGSPPQKLNILVDTGSSNFAVGAAAHPFLRRYYRRSLSSSYRDQGRSVYV

PYTQGRWEGELGTDLVSVPHGPNASLRANIAAITQSDRFFINGSNWEGILGLAYADIARP

DERLEPFFDSLVRQTPVPNLFSLQLCGAGFTQNYSLGSATVGGSMIIGGVDPSLYVGELW

YTPIRREWYYEVIIVRIEVNGQDLNMDCKEYNYDKSIVDSGTTNLRLPRKVFQAAVKAIE

AASSTEQFPSGFWLGEQLVCWQAGTTPWHIFPVISLYLMSENHNQSFRISILPQQYLRPV

EDVASAQEDCYKFAVSQSSTGTVMGAVIMEGFYVVFDRERKRIGFAVSTCHVHDEFRTAS

VEGPFHGVDLEDCGYNIPQTDESTLMTIAYIMAGICALFMLPLCLMVCQWRFARCLHPHG

DFADDISLLK

>Oryz_lati_BACE1

SFVDMIDNLRGKSGQGYYVEMAVGSPPQKLNILVDTGSSNFAVGAAAHPFLRRYYHRSLS

HSYRDLGRSVYVPYTQGRWEGELGTDLVSVPHGPNATLRANIAAIIQSDRFFINGSNWEG

ILGLAYAEIARPDEMLEPFFDSLVRQTSIPDLFSLQLCGAGFTQNYSLGSATVGGSMIIG

GVDPSLYVGELWYTPIRREWYYEVIIVRIEVNGQDLNMDCKEYNYDKSIVDSGTTNLRLP

RKVFQAAVKAIEAASSTEQFPSGFWLGEQLVCWQAGTTPWPIFPVITLYLMSENRNQSFK

ISILPQQYLRPVEDVASAQEDCYKFAVSQSSTGTVMGAVIMEGFYVVFDRQNKRIGFAVS

TCHVHDDLRTASVEGPYHGVELEDCGYNIPQTDESTLMTIAYIMAGICALFMLPLCLMVC

QWRFSRCINPNGDFADDISLLK

>Lat_chal_BACE1

IPYFPLLLVWMGIAVLVEVRGGQMRIRFHLKNGPGDPERHSRVRRSAASEAAQTINFVDM

IDNLRGKSGQGYYVEMTVGTPPQKLNILVDTGSSNFAVGAASHPFLRRHYRRHLSSSYRD

LRKTVYVPYTQGKWEGELGTDIVTIPHGPNVTIRANIAAITESDKFFINGSNWEGILGLA

YAEIARPDESLEPFFDSLVKQTQVPNIFSLQLCGAGFSLNDSDATVSVGGSMIIGGTDPS

LYIGNVWYTPIRKEWYYEVIIVKIEINGQDLKMDCKEYNYDKSIVDSGTTNLRLPKKVFE

AAVKSIKAASSTEKFPDGFWLGEQLVCWQIGSTPWHIFPVISLYLMGEFTNQSFRITILP

QQYLRPVEDVATSQDDCYKFAISQSTTGTVMGAVIMEGFYVVFDRAQKRIGFAVSSCHVH

DEFRTAAVEGPFFTANMEDCGYNIPQTDESTLMTIAYIMAAICGLFMLPLCLMVFQWRCF

RCLRRDQDDFADDISLLK

>Gadu_morh_BACE1

CVLDGSTGHSAVPNTSGLPLAIRLPLRQGARTRRRRSGVNFVDMIDNLRGKSGQGYYVEM

AVGSPPQKLNILVDTGSSNFAVGAASHPFLRRYYHRSLSHSYRDLGRSVYVPYTQGRWEG

ELGTDLVSVPHGPNATLRANIAAIIQSDRFFINGSNWEGILGLAYAEIARPDEMLEPFFD

SLVRQTSIPDLFSLQLCGAGFTQNYSLGSATVGGSMIIGGVDPSLYVGELWYTPIRREWY

YEVIIVRIEVNGQDLNMDCKEYNYDKSIVDSGTTNLRLPRKVFQAAVKAIEAASSTEQFP

SGFWLGEQLVCWQAGTTPWPIFPVITLYLMSENRNQSFKISILPQQYLRPVEDVASAQED

CYKFAVSQSSTGTVMGAVIMEGFYVVFDRQNKRIGFAVSTCHVHDDLRTASVEGPYHGVE

LEDCGYNIPQTDESTLMTIAYIMAGICALFMLPLCLMVCQWRFSRCINPNGDFADDISLL

K

>Oreo_nilo_BACE1

SSGLSLAIRVPLRQGARGEQPPPLPPAASASRSPARRRRGAAAGGISFVDMIDNLRGKSG

QGYYVEMAVGSPPQKLNILVDTGSSNFAVGAAAHPFLRRYYHRSLSTSYRDLGRSVYVPY

TQGRWEGELGTDLVSVPHGPNATLRANIAAITQSDRFFINGSNWEGILGLAYADIARPDE

KLEPFFDSLVRQTSVPNLFSLQLCGAGFTQNYSLGSATVGGSMIIGGVDPSLYVGELWYT

PIRREWYYEVIIVRIEVNGQDLNMDCKEYNYDKSIVDSGTTNLRLPRKVFQAAVKAIEAA

SSTEQFPSGFWLGEQLVCWQAGTTPWHIFPVISLYLMSEVRNQSFRISILPQQYLRPVED

VASAQEDCYKFAVSQSSTGTVMGAVIMEGFYVVFDREKKRIGFAVSTCHVHDEFRTASVE

GPFHGVDLEDCGYNIPQTDESTLMTIAYIMAGICALFMLPLCLMVCQWRFARCLHPHGDF

ADDISLLK

>Hum_BACE2

MGALARALLLPLLAQWLLRAAPELAPAPFTLPLRVTAATNRVVAPTPGPGTPAERHADGL

ALALEPALASPAGAANFLAMVDNLQGDSGRGYYLEMLIGTPPQKLQILVDTGSSNFAVAG

TPHSYIDTYFDTERSSTYRSKGFDVTVKYTQGSWTGFVGEDLVTIPKGFNTSFLVNIATI

FESENFFLPGIKWNGILGLAYATLAKPSSSLETFFDSLVTQANIPNVFSMQMCGAGLPVA

GSGTNGGSLVLGGIEPSLYKGDIWYTPIKEEWYYQIEILKLEIGGQSLNLDCREYNADKA

IVDSGTTLLRLPQKVFDAVVEAVARASLIPEFSDGFWTGSQLACWTNSETPWSYFPKISI

YLRDENSSRSFRITILPQLYIQPMMGAGLNYECYRFGISPSTNALVIGATVMEGFYVIFD

RAQKRVGFAASPCAEIAGAAVSEISGPFSTEDVASNCVPAQSLSEPILWIVSYALMSVCG

AILLVLIVLLLLPFRCQRRPRDPEVVNDESSLVRHRWK

>Mouse_BACE2

MGALLRALLLPVLAQWLLSAVPALAPAPFTLPLQVARATNHRASAVPGLGTPELPRADGL

ALALEPVRATANFLAMVDNLQGDSGRGYYLEMLIGTPPQKVQILVDTGSSNFAVAGAPHS

YIDTYFDSESSSTYHSKGFDVTVKYTQGSWTGFVGEDLVTIPKGFNSSFLVNIATIFESE

NFFLPGIKWNGILGLAYAALAKPSSSLETFFDSLVAQAKIPDIFSMQMCGAGLPVAGSGT

NGGSLVLGGIEPSLYKGDIWYTPIKEEWYYQIEILKLEIGGQNLNLDCREYNADKAIVDS

GTTLLRLPQKVFDAVVEAVARTSLIPEFSDGFWTGAQLACWTNSETPWAYFPKISIYLRD

ENASRSFRITILPQLYIQPMMGAGFNYECYRFGISSSTNALVIGATVMEGFYVVFDRAQR

RVGFAVSPCAEIEGTTVSEISGPFSTEDIASNCVPAQALNEPILWIVSYALMSVCGAILL

VLILLLLLPLHCRHAPRDPEVVNDESSLVRHRWK

>Rat_BACE2

MGALLRALLLPLLAQWLLRAVPVLAPAPFTLPLQVAGAANHRASTVPGLGTPELPRADGL

ALALEPARATANFLAMVDNLQGDSGRGYYLEMLIGTPPQKVRILVDTGSSNFAVAGAPHS

YIDTYFDSESSSTYHSKGFEVTVKYTQGSWTGFVGEDLVTIPKGFNSSFLVNIATIFESE

NFFLPGIKWNGILGLAYAALAKPSSSLETFFDSLVAQAKIPDIFSMQMCGAGLPVAGSGT

NGGSLVVTVFPQVLGGIEPSLYKGDIWYTPIKEEWYYQIEILKLEIGGQSLNLDCREYNA

DKAIVDSGTTLLRLPQKVFDAVVEAVARTSLIPEFSDGFWTGAQLACWTNSETPWAYFPK

ISIYLRDENASRSFRITILPQLYIQPMMGAGFNYECYRFGISSSTNALVIGATVMEGFYV

VFDRAQRRVGFAVSPCAEIAGTTVSEISGPFSTEDIASNCVPAQALNEPILWIVSYALMS

VCGAILLVLILLLLFPLHCRHAPRDPEVVNDESSLVRHRWK

>Dog_BACE2

MVWGKAGQLQILVDTGSSNFAVAGAPHSYTDSYFDADRSSTYRSKGFDVTVKYTQGSWTG

FVGEDFVTIPKGFNSSFLVNIATIFESENFFLPGIKWNGILGLAYAALAKPSSSLETFFD

SLVAQAKIPNVFSMQMCGAGLPVAGSGTNGGSLVLGGIEPSLYKGDIWYTPIKEEWYYQI

EILKLEIGGQSLNLDCREYNADKAIVDSGTTLLRLPQKVFNAVVEAVARTSLIPEFSDGF

WTGSQLACWTNSETPWSYFPKISIYLRDENSSQSFRITILPQLYIQPMMGAGLNYECYRF

GISPSTNALVIGATVMEGFYVVFDRARKRVGFAASPCAEIAGAPVSEISGPFSTDDIASN

CVPALPLNEPILWIVSYTLMSVCGIILLILIVLLLLPFRCRHLPRDPEVVNDESSLVRHR

WK

>Cow_BACE2

MSVEHVPGHWGHYAQESSPGSALPVAGYVGALDVDVGCQPGNLAFQAAGLCTHILLGCWL

QILVDTGSSNFAVAGAPHPYIDSYFDAERSSTYRPKGFDVTVKYTQGSWTGFVGEDVVTI

PKGFNSSFLVNIATIFESENFFLPGIRWNGILGLAYATLAKPSSSLETFFDSLVAQAKIP

NIFSMQMCGAGLPVAGSGTNGGSLVLGGIEPTLYKGDIWYTPIKEEWYYQIEILKLEIGG

QSLNLDCREYNADKAIVDSGTTLLRLPQKVFDAVVEAVARTSLIPEFSEGFWTGSQLACW

TNSETPWSYFPKISIYLRDENSSRSFRITILPQLYIQPMMGAGLNYECYRFGISPSTNAL

VIGATVMEGFYVVFDRAQKRVGFAASPCAEIAGAPVSEISGPFSTEDIASNCVPALPLNE

PVLWIVSYTLMSVCGVILLVLITLLLLPVRCRHHPRDPEVVNDESSLVRHRWK

>Mono_dome_BACE2

SGGTQVLLFLQLFFPWTAQAQAQAPAQAPAPFIFPLKVSPGGRKGISPRPPQVRASVAAP

LGNEPGGQVSAVAAGGLALALDPERGAVSFLPMVDNLQGDAGKGYYVEMLIGTPPQKLQI

LVDTGSSNFAVAGVPHPYVTSYFDFEKSSTYRSKNLNINVKYTQGSWTGSVGEDMVTIPK

GFNSTFLVNIAIIFESKDFFLPKTKWDGILGLAYAMLAKPSSSLETFFDSLVKQVKIPNI

FSIQMCGAGLPRDKTGTSGGSLVMGGIEPSLYEGDIWYTSIKKEWYYQIEILKLEIGGQS

LNLDCIEYNVDKAIVDSGTTLFHLPKKVFDAVIEAVSHTSLISEFSEGFWAGSQLACWAN

YETPWLYFPNISIYMRDENSSQAFRITILPQLYILPILGTDSKYECYRFGISSSTNSLII

GATIMEGFYVVFDRAQKRIGFALSLCAEVGGVPVSEVSGPFSTDDIASSCLHVSPLPDSV

LWIVSYALMSLCAVIFLVLFIALLFPFCRLFSSRDPNLVNDESRLVQHRWK

>Orni_anat_BACE2

YITEVLGLVMHILVDTGSSNFAVAGSPHSDISSYFDAERSSTYSSKNFDVTVKYTQGSWT

GSVGTDVITIPKGFNGSFVVNIATIFESENFFLPGIQWNGILGLAYAALAKPSSSLETFF

DSLVKQAKIPNIFSMQMCGAGLPVAGTGINGGSLVMGGIESSLYTGDIWYTPIKEEWYYQ

IEILKLEVGGQNLNLDCREYNANKAIVDSGTTLLRLPQKVFEAVVETITSTSSIQDFAEG

FWTGSQLACWSNSDKPWSLFPKISIYLRDENSSRSFRITILPQLYIQPMMGVASNYECYR

FGISSSTNALVIGATVMEGFYVVFDRAQKRVGFAVSLCAEVAGSPVSEISGPFPTDDVAS

NCVTTLPFNEPILWIVSYTLMSICGIILLVLIILLLLPHRCQRRPRDPDTVNDESSLVRH

RWK

>Ailu_melan_BACE2

YLEMLIGTPPQKLKILVDTGSSNFAVAGAPHSYIDSYFDAERSSTYRSKGFDVTVKYTQG

SWTGFVGEDVVTIPKGFNSSFLVNIATIFESENFFLPGIKWNGILGLAYAALAKPSSSLE

TFFDSLVAQAKIPNVFSMQMCGAGLPVAGSGTNGGSLVLGGIEPSLYKGDIWYTPIKEEW

YYQIEILKLEIGGQSLNLDCREYNADKAIVDSGTTLLRLPQKVFNAVVEAVARTSLIPEF

SDGFWTGSQLACWTNSETPWSYFPKISIYLRDENSSRSFRVTILPQLYIQPMMGAGLNYE

CYRFGISPSTNALVIGATVMEGFYVIFDRARKRVGFAASPCAEMAGAPVSEISGPFSTDD

IASNCVPALPLNEPILWIVSYALMSVCAAILLILIVLLLLPCPCRQLPRDPEVVNDESSL

VRHRWK

>Xeno_laev_BACE2_A

MKVPLVRLLLLCAAACASNKFIVPLNVSPAEIKGTLPVAPATPKDKPGLLLASDPGGTIN

FFSMVDNLAGDSGRGYYLELLIGSPPQKVNILVDTGSSNFAVAGSPNPDVNTFFDSKLST

SYQSLNTEVTVRYTQGSWTGLLGKDVVSIPKGVNGTFLINIASIFQSESFFLPNINWQGI

LGLAYSTLAKPSSSVEPFFDSLVQQENIPDVFSMQMCGAGQSSPGNGINAGSLVLGGVEP

SLYKGNIWYTPITEEWYYQVEVLKFEVGGQRLNLDCTVYNSDKAIVDSGTTLLRLPDKVF

NAMVDAIVQTSLIQNFNAEFWAGLQLACWDKTQQPWNYFPDISIYLRDTNTSRSFRLTLK

PQLYIQSVLTFQESLNCFRFGISQSASTLVIGATVMEGFYVIFDRAEKRVGFAVSSCAEV

SGITVSEIAGPFGTSDVSSNCIARNPLREPIMWIISYSLMSLCGMILLVLVILLLLSNRQ

RHDDMETINDESSLVQHRWK

>Xeno_laev_BACE2_B

MKIPFLSLLVLCAACASSKFIVPLKVSPSEITGTLPVASATPKDTPGLLLASDPGGTINF

FSMVDNLEGDSGRGYYMELLIGTPPQKMNILVDTGSSNFAVAGSPNPDVKTFYDSKLSTS

YQHLNTEVTVRYTQGSWTGLLGKDVVSMPKGVNGTFLINIASILQSDNFFLPNINWQGIL

GLAYSTLSKPSSSVEPFFDSLVQQRNIPDIFSMQMCGAGQPTPGNGINAGSLVLGGIEPS

LYKGDIWYTPITEEWYYQVEVLKFEVGGQNLNLDCTVYNSDKAIVDSGTTLLRLPDKVFN

AMVDAIVQTSLIQNFNAEFWAGLQLACWDKTQQPWNYFPDISIYLRDTNTSQSFRLTLKP

QLYIQSVLTLQESLNCFRFGISHSASALVIGATVMEGFYVIFDRTEKRVGFAVSSCAEVS

GITVSEIAGPFGTSDVSSNCIVRNPLREPIMWIISYSLMSLCGIILLVLVILLLIPNRHR

RDDMETVNDESSLVQHRWK

>Xeno_trop_BACE2

MKVPLLAVLLLGAACASANFIFPLKVSPAEMKGAVPVAPATPKGAPGLLLASDPGGQINF

FSMVNNLEGDSGRGYYLELLIGTPPQKMNILVDTGSSNFAVAGALNPDITTFFDSKLSTS

YEPLNTQVTVRYTQGSWTGLLGKDVISMPKGVNGTFLINIASIFQSENFFLPNINWQGIL

GLAYSTLAKPSSSVEPFFDSLVQQENIPNIFSMQMCGAGQPSPGIGINAGSLVLGGIEPS

LYQGDIWYTPITEEWYYQVEVLKFEVGGQNLNLDCTVYNSDKAIVDSGTTLLRLPDKVFN

AMVDAIVQTSLIQNFNAEFWAGLQLACWDKTQDPWNYFPDISIYLRDTNSSRSFRLTLKP

QLYIQSVLTFQESLNCFRFGISQSASALVIGATVMEGFYVIFDRAEKRVGFAVSSCAEVS

GITVSEIAGPFGTSDVSSNCIARNPLREPIMWIISYSLMSLCGMILLVLVILLLLPNRHR

RDDMETVNDESSLVQHRWK

>Anol_carol_BACE2

QLNILVDTGSSNFAVAGTPNTDVTSYFNSEKSSTYKPLGIDVAVKYTQGSWIGTLGTDVI

TMPKGINGTYTINIASISQSENFFLQGIQWQGILGLAYDALAKPSGSLETFFDSLVNQAK

IPNIFSLQMCGAGLPVSGTGTNGGSLILGGIEPSLYKGEIWYTPIQREWYYQVEILKLEV

GGQNLNLDCKEYNSDKAIVDSGTTLLRLPEKVFSAVVGAIIQTSLIQDFPGGFWSGTQLA

CWIKTEKPWTFFPEISIYLRDENVSRSFRITILPQLYIQPVLEYGQNLGCYRFGISSSDS

ALVIGATVMEGFYVIFDRAQKRVGFALSTCAEMDGSPVSEIKGPFTTADVASTCVSTLSL

HEPLLWIVSYTLMTFCGLVLLVLVILLLLPSRCRQRTANNIVNDESSLVRHRWK

>Gall_gall_BACE2

RQLNILVDTGSSNFAVAGVPDPDVTSYFNTELSSTYQSQGIEVTVKYSQGSWTGVLGTDV

VTIPKGIDGRYTINIATILESENFFLPGVKWHGILGLAYDTLAKPSSSVETFFDSLVKQA

KIPNIFSLQMCGAGLPVSGSGTNGGSLVLGGIEPSLYKGNIWYTPIKEEWYYQVEILKLE

VGGQNLELDCREYNADKAIVDSGTTLLRLPQKVFSAVVQAIARTSLIQEFSSGFWSGSQL

ACWDKTERPWSLFPKLSIYMRDENSSRSFRISILPQLYIQPILGIGENLQCYRFGISSST

NALVIGATVMEGFYVIFDRAQRRVGFAVSPCAEVDGSPVSEIEGPFTTTDVASNCVSSIT

FHEPVLWIASYALMSLCGIILLILIILLLIPPRCQHRYTDNDVVNDESSLVRHRWK

>Taen_gutt_BACE2

MAGHNQPLLSGIHVLLINGAKNVLGLPSCLPEPGLCQDCQPREQERCPDIVPRRKEDAGS

EERRSSAAALPRSRSGKLNILVDTGSSNFAVAGVPDPDVTSYFNPELSSTYRSEGIEVTV

KYSQGSWTGVLGTDVITMPKGLYGSYTINIATILESENFFLPGVKWHGILGLAYDALAKP

SSSVETFFDSLVKQAKIPNIFSLQMCGAGLPVSGSGTNGGSLVLGGIEPSLYSGDIWYTP

IKEEWYYQVEILKLEVGGQNLQLDCREYNADKAIVDSGTTLLRLPEKVFSAVVQAIARTS

LLYIQPILLIGDNMQCYRFGISSSTNALVIGATVMEGFYVIFDRAQRRVGFAVSPCAEVD

GSPVSEIEGPFTTEDVASNCVSSVSFHEPVLWIASYALMSLCGIILLVLIVLLLIPPRCQ

HRYTDNDVVNDESSLVRHRWK

>Dani_reri_BACE2

MRLYGLLLLSLTFWKSHSVFKIPLNIFAGKFNASVQLDLRPLQKNEAAAKSGLSLASDPA

GIVNFLDMINNLKGDSGRGYYMQMIIGTPGQTLNILVDTGSSNFAVAAAAHPYITHYFNR

ALSSTYQSTERAVAVKYTQGEWEGELGTDLITIPEGPSGAITINIAAILTSEGFFLPGIN

WQGILGLAYPLLARPDPSVEPFFNSVVRQTGIPDVFSLQMCGAGVPASTTGDPAGGSLIM

GGVEPTLHRGPIWYTPVLEEWYYQVEVLKLEVGDQNLNLDCKEYNSDKAIVDSGTTLLRL

PANVFTAVVDTIMQTSLIDDFSAGFFDGTKLACWMRGESPWRFFPKVSIYLRGTNTSQSF

RITILPQLYIQPVTDIDGTLDCFRFGISPSANGLVIGATVMEGFYVIFDRAQKRVGFAVS

TCAENGGVPFAEIAGPFLAEGVASDCTSGMTLREPVHVGDRIHSDGRLCCGSHHTSRLAH

TALPTTHRRRDYR

>Taki_rubr_BACE2

SLASDPTGTVNFLDMVNNLKGDSGRGYYIELSIGTPGQKMNILVDTGSSNFAVAAAPHPF

ITHFFNTALSSSYQYTGQGVAVRYTQGNWNGELGMDLVSIPEGPNGTFPINIAAILSSEG

FFLPDINWQGILGLAYPLLARPDPSVTPFFDSLVQQLGIPDIFSLQMCGAGLSASSTVDA

AGGSLIMGGTESTLYTGPVWYTPIVEEWYYQVEVLKLEVGNQNLELDCKEYNTDKAIVDS

GTTLLRLPVNVFNALVTAITRSSLVRFRPHGTEKRRADLSHLCQIQEFSSGFWDGTKLAC

WMKGETPWRFFPKLSLYLRATNSSQSFRLTILPQLYIQQITDVDGTLDCFRFGVSSSVNG

LVIGATVMEGFYVVFDRAQRRLGFALSNCAVSAGLPLSEIAGPFSAADVASNCSGGVRNE

PVLWVISYALIAVCALVLIVLLLLLVVPCRRRNSFGEITDESSLVRHRIK

>Tetr_nigr_BACE2

ALASDPTGAVNFLDMVDNLRGDSGRGYYIEMSIGTPGQKMNILVDTGSSNFAVAAAPHPF

ITHFFNTALSTSYRSAGRPVAVRYTQGNWEGELGVDLVSMPTGPNGTVPINIAAILSSDG

FFLPDIHWQGILGLAYPLLARPDSSVTPFFDSLVRQLGIPDVFSLQMCGAGLSAGAAAAA

ALGGSLIMGGTEPTLHRGPVWYTPIVEEWYYQVEVLKLEVGDQNLDLDCREYNTDKAIVD

SGTTLLRLPVPVFNALVDAITRSSLVRRRSRAGLTAGVDLPLICQIQDFSAGFWDGSKLA

CWMKGETPWRFFPKLSIYLRATNTSESFRITILPQLYIQQITDLDGTLDCFRFGVSSSVN

GLVIGATVMEGFYVVFDRAQRRLGFALSTCAASGGLPLSEIAGPFSAADVASNCSGGVLK

APVLWVLSYALIAVCALVLVVLLLLLLLAPCRRRNSFGEITDQSALVRHRIK

>Oryz_lati_BACE2

FPRHFLRVTILSFGLSKCHFSVPLRIHPGKFNLSAHVNVSQLHRVALKSDGSGLSFASDP

AGIVNFLDMVGNLQGDSGRGYYIEMSVGTPGQKLNILLDTGSSNFAIAAAPHPDITRYFD

TDLSSSFQALDRFVAVRYTQGNWEGELGIDSVSIPKGPNGTITVNIAAILSSNGFFLSGI

KWQGILGMAYPALARPDSSVEPFFDSVVKQLGIPDVFSLQMCGAGLSGSSAADPPGGSLI

MGGAEPTLYRGSVWYTPIVEEWYYQVEVLKLEVDDQNLNLDCKEYNMDKAIVDSGTTMLR

LPAKVFNAVVEAITRSSLIQEFSSGFWEGTKLACWMKGETPWRVFPKLSIYLRATNTSQS

FRITILPQLYIQLITDENSALDCFRFGVSSSANGLVIGATVMEGFYVVFDRAHKRLGFAL

SSCAAVSGMHITMASMP

>Gast_acul_BACE2

MARAAAAAVSTRAFIFSVFLGVSQCYFAVPLKISTGSYNVSSDVGVTRRVVLTADGDGLS

LASDPSGAVNFLDMVNNLQGDYGRGYYIEMSIGTPGQKVNILVDTGSSNFAVAAAPHPFI

THYFDAALSGTYQSSGRTVAVRYTQGNWEGELGTDRVSIPNGPNGTATINIAAILSSEGF

FLPGVNWQGILGLAYPMLARPDSSVVPFFNSLVQQLGIPDIFSLQMCGAGISASSTADPP

GGSLIMGGIEPTLYQGSVWYTPIVEEWYFQVEVLKLEVGDQNLDLDCREYNMDKAIVDSG

TTLLRLPVNVFNAVVEAITRSSLIQDFSSGFWDGSKLACWMKGETPWRFFPKLSIYLRAT

NTSQSFRITILPKLYIQPITDVDGTLDCFRFGLSSSANGLVIGATVMEGFYVVFDRAQRR

LGFALSSCAVSGGEALSEVAGPFSAADVAANCSWAPPKEPFMWVISYALMAVCAVVLIML

LLLLVLPCRRNRSGEITDESSLVRHRIK

>Gadu_morh_BACE2

FFFRLCFDICDGHFAVPLKIYLGKFNFSRDVDLTPLQQTPGVGGKLLSLASDPAGIVNFL

DMINNLEGDSGRGYYIEMSIGTPAQKLNILVDTGSSNFAVASTPHPFITHYFNTSLSTTY

QPTGRTVAVRYTQGNWEGGLGIDRVSIPKGPNQTIVINIAAISASEGFYLPGVNWQGILG

MAYPLLARPDSSVEPFFNSVVRQTGIPDIFSLQMCGAGVSAGGATDPPGGSLIMGGAEPT

LYRGVMWYTPIKEEWYYQVEVLKLEVGEQGLNLDCSEYNMDKAIVDSGTTLLRLPANVFN

AVVDAITRTSMIQDFSSGFWEGTKLACWMKGETPWRFFPKISIYLRASNTSESFRITILP

QLYIQPITDVDSTLDCFRFGVSSSANGLVIGATVMEGFYVVFDRAQKRVGFAVSSCAVSG

GAALSEIAGPFSASDVGTDCAGRSLNEPLLWVISYAMMALCATILFTLLLLLVMPCRRRH

RSGDITDQSSLVRHRIK

>Lat_chum_BACE2

MKLLLGLLAFLALAGLGRAYIYLPLNISFGFNSSWSPSPEQPERTDGRQGQELELALASD

PSGKVRFIEMVNNLKGGSGRGYYLPMLIGTPVQKLNILVDTGSSNFAVAGAPDPSISAYF

KPQLSSTYRNLDTSVSVTYTQGSWIGELGTDVVTLSQGLNSSITVDIAVILESENFYLEG

AKWQGILGMAYKPLSMPSGSVETFFDSLVKQTNIPDVFSLQMCGAGLPVSTDIEDIPAGG

SLILGGVEPSLYVGDVWYTPITKEWYYQVEILKLEVGGQNLHLDCKEYNAGKAIVDSGTS

LLRLPNNVFDAVVEAIIWTSAVQTFSAEFWTGAEMACWSKGATPWNFFPKISIYLRGENA

SQSFRITVLPQVRALICLMHVL

>Oreo_nilo_BACE2

MAHSTFVSFGAFALSLYFGVSYCYFSIPLKIYPGKYNVSSDVNLTSLRRVALKSDGSGLS

LASDPTGTVNFLDMVNNLQGDSGRGYYIEMSIGTPGQKLNILVDTGSSNFAVAAAAHQFI

THYFNTALSSTYSSTGRTVAVRYTQGNWEGELGIDRVSIPRGPNGTITINIAAILSSDGF

FLPGINWQGILGLAYPVLARPDSSVEPFFNSVVKQLGIPDIFSLQMCGAGLSASSTTDTT

GGSLIMGGAEPTLYRGSVWYTPIIEEWYYQVEVLKLEVGDQNLNLDCREYNMDKAIVDSG

TTLLRLPVNVFNALVEAITRSSLIEEFSSGFWEGTKLACWMKGENPWRFFPKLSIYLRAT

NTSESFRITILPQLYIQPITDVDGTLDCFRFGVSSSTNGMVIGATVMEGFYVVFDRTQQR

LGFALSSCAVSDGVALSEIEGPFSAADVAADCSGGPLKEPLVWMISYALMAVCALALLIL

LLMLILPCRCGNRNGEITDESSLVRHRIK

>Plan_schm_cath01

MNIKSLIIALVINGIFGGLLRVPLKKMESARSNIVKFNMMLNHFMKSEIKNPIPEPLDNY

MDAQYFGEITIGTPPQPFTVLFDTGSSNLWVPSKKCKLTNLACLTHRKYDSSKSSTYVKN

GTAFSIRYGTGSLSGFLSTDTVIVATAKVVSQTFGEAMVEPGTTFVVAKFDGILGMAYPS

ISVDHVTPVFQNMVAQKLVDAPIFSFYLNRDVNGKPGGEIIFGGSDPNYYQGPFTYTPVT

SKTYWQFSMQSISMLGKTYCTNCTAIADTGTSLIVGPQSIIDDINSKFNGTKINGMTIIL

CSSVSKLPVINIQIAGRNFPLKPSDYILKLQDTVCVSGFTSLSGIPFFILGDVFIGRYYT

EFDLGNSRIGFAPVKTPSKDETLYYKPLF

>Tric_adhe_cath01

MFAFILNILYSIICISTLSLASGNGIYDGSLTNIPIEHHRLENDRIYLDRVRGSGQAGYS

MLIWLGTPPQKLRVILDTGSSFCGIMAAPSPVVKHYFHMNRSSTLEETNLRIDSSYVKGY

WSGQLVSDMLHLGIGLHKQVRIQFAAITNQKEFFTETTRFDGILGLAYPSLAVQGNFYQK

PVFNEIVQQAGIRDIFTLTYCASKMRKDLFGNQYITGGGFMTLGGIDNNLLAGPVFYTPI

VEKYYYQFQLTNVLVDGQSIGFSPYDYMHYPALVDSGTSILRFPPFMYKRLMPIFLRSIQ

DRSVFSHGFFYRGHAVCMEESQLLQHRFPTIRLSIRLASFEKTNFKTPRQFTLVLSPMQY

FILSGKERHGKPCYHFGIAGTSGAFGIILGDVVMKGFSVTFDRVNSMLGFAVSKCAGLKL

QTAYSKQF

>Mono_ovat_cath01

MMRTAVLLLALVAAAAALSKVTLHGMERTRDSLRRQGAMLTTKYQNIMAGTNVPLSNYED

AQYFGEISIGTPAQKFKVIFDTGSSNLWVPSSQCPKTNIACDVHAKYDSSASSTYKANGT

KFAIQYGTGSLSGFLSTDTACIGDLCVKDQTFAEALEEPGVTFVAAKFDGILGMGFSTIS

VDHVVPVWYNMVQQQVVEQNMYSFYLNRNPNGVSGGELTLGGYDESHFAGPIHWTDVTVD

GYWQFTMTGLSIENTPYCTNCKAIADTGTSLLAGPTDVVKQINKAIGATTIAAGEAIVDC

NKIPHMPNVTIVINGIQYSLSAEQYVLQVTAEGETECISGFAGIDVPAPEGPLWILGDVF

IGAYTTVFDMGNNRVGFGASA

>Bran_flor_cath01

MKVLYVLLAIVAIASALHRIPLTKMKTLRRQLADVGIIYDQIMGHKGYNGTYDNIQDAPE

PLHNYLDAQYYGTIAIGTPPQSFQVVFDTGSSNLWVPSSHCPLTDIACCTLYRRVPLTTG

LVQIDKIKMMSSTKTSSPILPRIDDCECTPSVQHIFRRHYLLQSPMYYIRWIYAILYSLY

LLFVLRTPKDRDIVGYIENTTMAMLIRPATDGKSGEYEVTVCDCKLRASRGYKLNNMSLR

DPLGTTGGELLLGGTDPKYYSGDFTFVNVTEPGYWQFKMDGIMINGQASAYCKGGCNAIA

DTGTSLIAGPTSEIQALNKLIGATPIVGGEYTVDCNKIPSLPTISFVLGGKSFGLKGEDY

VLKVSTMGQTECISGFLGIDVPPPRGPLWILGDVFIGPYYTQFDLGNNRVGFARAAYNNA

TRV

>Nema_vect_cath01

FYRIPLHKMPTPRQSLKEVGISVEQLLGKYGGKYEGGDVPEPLINYMDAQYYGEITIGTP

PQKFTVVFDTGSSNLWVPSKKCSWTNIACLLHDKYDSTKSSTYKKNGTEFAIRYGSGSLS

GFLSIDTVSVGGIDVKGQTFAEALKEPGLTFVAAKFDGILGMGFSSISVDQVVPVFYDMV

LQKLVPAPVFSFYLNREPGASPGGELLLGGSDPKYYKGNFSYVPVTQEGYWQFKMDGISV

KEGSFCSDGCQAIADTGTSLIAGPTDEIEKLNNLIGAKIIIGGEYTVNCSAIDSLPDITF

TIGGKKYVLTGKQYILKVTTLGQSVCISGFLGLDVPPPRGPLWILGDVFIGPYYTEFDFG

NKRVGFAEAV

>Stro_purp_cat01

MKTFLAVLLLAVAAHAQLVRVPLYKMETVRRQMANTGLPFKDLSQLSNKYNMMNNNRLGA

PWPINMSDYLDAQYYGPISLGTPPQEFTVVFDTGSADLWVPSSQCGFLDIACKFHRKYDS

SKSSTYKKNGTKWAIQYGSGSCSGFVSNDVIELGALKAKNQSFGEATAEPGLTFVAAKFD

GILGLGYPTITRISKVPVFEKIVQQGLVTDAXXLSVGNNGTYCTNCAAIADTGTSLIAGP

SAEIAKLQLQIGAAPFASGEVQQGTVTLCISGFIGLDVPPPAGPLWILGDPFLRTYYSKF

DRTNNQLGFATAV

>Cion_inte_cath01

MKVACVFLAILAVCSAAHVIKLRKQKTLRQYMKEKGTSPSKYTQHWARQTSNEPLTNYMD

AQYFGEISIGTPEQTFTVIFDTGSSNLWVPSASCPSTNYACMTHNKYNSAASSTYVADGE

EFRIQYGTGSMVGYDSVDTVKIAGVPSTSQTFAEALEEPGITFVAAKFDGILGMGYPNIA

VNGMKPVFNQMFEQGAVDQNLFAFYLNRDPEAADGGEITLGGVNPARYVGDFNYHDVTRQ

GYWQIKMDGLSIADTAKTTACNGGCQVIVDSGTSLITGPSADTDAINQAIGAIKFVQGEY

LVICRRIPEMPDITFVLDGIEYVLTPQDYVIQMTADGQTQCLSAFMGMDIPEPTGPLWIL

GDAFMGKFYTSFDFGTNQVGFAKLA

>Cion_sauv_cath01

MQVRIKLHKQKTLRQIMKEKGTKAFKYTPSFGRSTSNEPLTNYLDAQYFGEIAIGTPEQK

FTVIFDTGSSNLWIPSATCPSNNYACLLHNKYDSTASSTYVADGDNFAIQYGTGSMVGFE

SVDTVKIAGIPSTNQVFAEATEEPGITFVAAKFDGILGMGYPTISVNGIKPVFNQMYEQG

AVDKNQFAFYLNRDPTGEEGGEICLGGVNEARYTGEFNYHSVTRKGYWQLKMDSVAVADA

DATAACVGGCQIIVDSGTSLITGPTVDTDAINHAIGAIRFVQGEYLVICRNIPNMPDITF

VLDGIEYVLTPDDYVLKITQDGVTQCLSAFMGMDIPEPTGPLWILGDAFMGKYYTSFDFD

TDQVGFAKLA

>Cras_giga

MVTGPHCSRKRGLIYDTCKWILAFTLLVNTLHVNAGSFPTLPLKVGYIHNVGHDRYRRSD

QFQSQRNNLKGKPGQGYYVEMEIGTPTQKINVLIDTGSSNFAIAATPDPDIDKFFQRENS

STYSLIGTSVYVPYTQGNWKGTLGSDLVTLTSLPNVTVTANIAFITESKQFFINGSNWQG

ILGLAYSSIARPDQSVTPFLDSLIDNTGITDVFSIQLCGDKFEQQTDKDVELGGTITFGG

MDPKLHSGPVFFTNIHKKWYYEVVIVDVQVNGESINLDCKEYNFPKTIVDSGTTDLRLPN

KVYTSVIDHIKNYVQNSPKAPALTHGFWTGDIDLCYKDGAIPYDIFPFVALGFAENDQQS

FKIIVAPQQYIKSVGKDDENPDEICFKLGIASSSSGTVIGAVVMEGFYVVFDRQSNRIGF

ADTTCPAVAQQFNRSHMSGPYQFSGSVGNCAYAKPEKSNKTLTIVAYVLAGICGACILPL

LFLFVQYQWRTSKCYEKFKRQNSDGDSNDLIDEPSFMHES

>Pelo_sine_BACE1

PGLLRGDDSREPPAKGNHLGIRVPLRNGMGTLQQGPRVQRSVAEEQEDSRQRSSFVNMID

NLRGKSGQGYYVEMTLGSPPQKVEEQEDSRQRSSFVNMIDNLRGKSGQGYYVEMTLGSPP

QKVIIMAHELPWLLLWLGAGEPPAKGNHLGIRVPLRNGMGTLQQGPRVQRSVAEEQEDSR

QRSSFVNMIDNLRGKSGQGYYVEMTLGSPPQKTYALGDRGPSRFAHAALPRPLAPRLPGE

RARSSTYRDLRKGVYVPYTQGKWEGELGTDLVTIPHGPNVTVRANIAAITESDKFFINGS

NWEGILGLAYAEIARPDDTLEPFFNSLVKQTRVPNIFSLQLCGAGFSPNESEALASVGGS

MVSAGAGLDGRDPRRGAVPGGREAEHSPRPLPAPSQAGRVLWWEGGAGLRAVSPLALGLC

SPALSFQQYLRPVEDVATSQDDCYKFAISQSSTGTVMGAVIMEGFHVVFDRARKRIGFAV

STCH

>Salm_salm_BACE1

NFVDMIDNLRGKSGQGYYVEMAVGSPPQKLNILVDTGSSNFAVGAAAHPFAAARYYHRSL

SKSYRDLGRSVYVPYTQGRWEGELGTDLVSVPHGPNASLRANIAAITQSDRFFINGSNWE

GILGLAYAEIARPDETLEPFFDSLVRQTPVPNLFSLQMCGAGFTQNYSLGSATVGGSMII

GGVDSSLYVGDLWYTPIRREWYYEVIIVRIEVNGQDLNMDCKEYNYDKSIVDSGTTNLRL

PRKVFQAAVKAIEAASSTEQFPSGFWLGEQLVCWQAGTTPWHIFPVISLYLMSENRNQSF

RISILPQQYLRPVEDVASAQEDCYKFAVSQSSTGTVMGAVIMGGFYVV

>Leuc_erin_BACEi

SDTVSVVGGSMIIGGVDTSLYLGNIWFTPIRKQWYYEVIIVKIEINGQDLNLDCKEYNYD

KSIVDSGTTNLRLPKKVFQAAVKSIKAASSTEKFPDGFWLGEQLVCWQVGTTPWHIFPTI

SLYLMGEATNQSFQITILPQQYLRPVEDVATSQDDCYKFAISQSTTGTVMGAVIMEGFYV

VFDRARKRIGFAVSTCHVHDEFR

>Squa_acan_BACE1

RPDENLEPFFDSLVKQTQVPNIFSLQLCGAGFSLNDTDSASSVGGSMIIGGVDSSLYLGN

IWFTPIRKEWYYEVIIVKIEINGQDLNLDCKEYNYDKSIVDSGTTNLRLPKKVFQAAVKS

IKAASSTEKFPDGFWLGEQLVCWQVGTTPWHIFPTISLYLMGEATNQSFQITILPQQYLR

PVEDVATSQDDCYKFAISQSTT

>Amb_tigr_BACE2

DKAIVDSGTTLLRLPQIVFDAVVGGITRTSLIQDFTSEFWAGTQLACWNKAEKPWLYFPD

LSIYLRDMNSSKSFRITVKPQLYIQYVLTMQGAMDCYRFGISSSTGALVIGATVMEGFYV

IFDRAQKKVGFAVSTCADVAGTLVSEITGPFPTNDVSSNCVATNSLREPIMWIVSYALMT

MCGIVLLILVILLLFPCRSNQDREVVNDESSLVRHRWK

>Pelo_sine_BACE2

QLNILVDTGSSNFAVAGAPTPDVTSYFNTEKSSTYKSQGTEVTVRYTQGSWVGVLGTDVI

TIPKGINGTYTVNVATILESENFFLPEVKWHGILGLAYDALAKPSSSVETFFDSLVSQAK

IPNIFSLQMCGAGMPVSGSGTNGGSLVMGGIEPSLYKGDIWYTPIKEEWYYQVEILKLEV

GGQNLNLDCREYNADKAIVDSGTTLLRLPQKVFNAVVEAIVRTSLIQTFSSGFWTGSQLA

CWDKTETPWSLFPKLSIYLRDENSSRSFRITILPQLYIQPLLGIGQNLECYRFGISSSTN

ALVIGATVMEGFYVIFDRAHKRVGFAVSPCAEVGGSPVSEIEGPFTTADIASNCVSTVTF

HEPMLWIISYALMSLCGIILLVLIILLLLPPRCQHRYTDNDVVNDESSLVRHRWK

>Onco_myki_BACE2

ERRGEELNILVDTGSSNFAVAAAAHPFITHFFNTALSSTYQSTGRGVAVKYTQGNWEGEL

GTDHVRIPSIPGTPTINIATILSSNGFFLPGVNWQGILGLAYPLLAQPDSSVEPFFNSVV

RQTGIPDVFSLQMCGAGLSDSTTADPAGGSLVMGGVEPTLYSGSMWYTPIKEEWYYQVEV

LKLEVGDQNLNLDCKEYNKDKAIVDSGTTLLRLPVNVFSAVVEAISRTSLIQDFTSGFWG

GTKLACWLKGETPWRFFPKLSIYLRATNTSQSFKISILPQLYIQPITDVDGTLDCFRFGI

SSSANGLVIGATVMEGFYVIFDRAEKRVGFAVSRCAVNGGIAVSEISGPFSSADVASDCA

AGGLLKEPLLWVISYALVGRLCRGAPHPAPPLSSALSSPRRGPRDH

>Salm_sala_BACE2

KSTGRGVAVKYTQGNWEGELGTDLVLIPSIAGTLTINIATILSSDGFFLPGVNWQGILGL

AYPLLARPDSSVEPFFNSVVRQTGIPDVFSLQMCGAGLSASTTADPTGGSLVMGGVEPTL

FSGSMWYTPIKEEWYYQVEVLKLEVGDQNLNLDCKEYNKDKAIVDSGTTLLRLPVNVFSA

VVEAISRTSLIQDFTSGFWGGTKLACWLKGETPWRFFPKLSIYLRATNTS

>Tric_adhe_C

MFAFILNILYSIICISTLSLASGNGIYDGSLTNIPIEHHRLENDRIYLDRVRGSGQAGYS

MLIWLGTPPQKLRVILDTGSSFCGIMAAPSPVVKHYFHMNRSSTLEETNLRIDSSYVKGY

WSGQLVSDMLHLGIGLHKQVRIQFAAITNQKEFFTETTRFDGILGLAYPSLAVQGNFYQK

PVFNEIVQQAGIRDIFTLTYCASKMRKDLFGNQYITGGGFMTLGGIDNNLLAGPVFYTPI

VEKYYYQFQLTNVLVDGQSIGFSPYDYMHYPALVDSGTSILRFPPFMYKRLMPIFLRSIQ

DRSVFSHGFFYRGHAVCMEESQLLQHRFPTIRLSIRLASFEKTNFKTPRQFTLVLSPMQY

FILSGKERHGKPCYHFGIAGTSGAFGIILGDVVMKGFSVTFDRVNSMLGFAVSKCAGLKL

QTAYSKQF

>Capi_tela

MRHPIVNVLCALFAALQSHARVIEIPLHQLTDELRESVSLDRYDVPHNSGEKNLQGQPGL

GYYIKMGIGTPPQQMNILIDTGSSNFALAAADNQAIDKYFDSSNSSTFSATQKQVSVPYT

QGNWQGYLATDVVTIPSMNITVDDATLSCIYSSENFFINGSNWQGILGLGYSQIARPDSS

VEPLFDAIVRNGIVNDTFSMQLCGNKFNGTAQGVLVGGTSLSQFLVLGGISESLYEGPIY

YAPIHEPWYYEVVLTDIKVGRESLNMDCKEYNYDKTIVDSGTTNLRVPKRVFQEIIERMR

MIMEALPIGGIPATFWDGSEKNILCPDDGTLPWETLPSIELHLGMTPNSSFGLVVAPRMY

LRPVHDIFDPLSNGTCYKFAITPSDTGTVIGAVVMEAFYVIFDRVSQQVGFASSSCGLSV

EALNHLINPYTNPGGDNWDCAYLRYHGAPNALQTVAYVMAGVCGACLIPLVVMVIHWQIV

RCKEHKSDDYGLLHEDHTLEQSTE

>Lymn_stagn

MLPPYISNCDIRAFISQIAGLFVQYKWHCMQSHFSIACVVILLLHTLVPSCAGVVYRFPV

RRFHSQVVMAKDDSNAALKSQLRIVQRDLSSGDLNDMKNNLNGFSGDGYYIDLNIGTPNQ

TMQVLIDTGSANFAIAASFDPKVRKFFHRENSSTYVQIGTEVHVAYTEGQWTGVLGTDIV

SVSSAPNISARAHIACITQTKDFFIPEAEWQGILGLGYSQLSMPDRSILPFWDSVVQERK

DLDDIFSMHLCGSSFSHTDEEALMEGSLIFGGVIESLSRGPILYTPIIKQWYYEVVLTDI

KVSGKSLKMKCKEFNFDKTIVDSGTTNIRLPTKVFQAVVDAIGQHIETNGLDNFVGASFY

NGIDVLCLQNVADFFSSFPIVSFSLYASENSSFQLDLSPQHYLRPVQTDMADLMLQSCVK

FGFSSSSSGTVLGAVLMEAFYVVFDRNSSRIGFGQTTCPLPDPKNPVLDFTIHGPFYSNK

NFSECAYKKSDSVDRSFLIISYIMAGLSIVVALPLFILCFLWIRRKVTVQDEGDTSETQS

VLPS

>Para_livi

MNLLKRVLMPFIWFVCILIDLTCAVARTHVYTLPLRKGTEISLAEIVDDPGTSHLNVSVK

DQRSNIRGRPGLGYYIEVDLGTPPQKLNVLIDTGSSNFAVAAAWHKAISTYYKTNESSSY

EDQGRHVKVPYTQGEWSGDLGRDLVQITSLGNQTFQANVAGITESEMFFLNGSRWQGILG

LAYAEIARPDNSVEPFFDSLTSQTSIRDIFALQMCGTLASENDTHLGSTGPVEEVIGSMD

IGGIDPSLFQAPMQYTPLRDEWFYEVIMTDIKVGNDSLGLDCKEYNFDKTIVDRLRK

>Chry_pict_BACE

MIDNLRGKSGQGYYVEMTLGSPPQLNILVDTGSSNFAVGAAPHAFLRRYYQRQSSTYRDL

RKGVYVPYTQGKWEGELGTDLVTIPHGPNVTVRANIAAITESDKFFINGSNWEGILGLAY

AEIARPDDTLEPFFNSLVKQTRVPNIFSLQLCGAGFFPNESEALASVGGSMVIGGVDHSL

HVGSIWYTPIRKEWYYEVIIVKMEINGQDLKMDCKEYNYDKSIVDSGTTNLRLPKKVFEA

AVKSIKTVSSTEKFPDGFWLGEQLVCWQVGTTPWHIFPVISLYLMGEATNQSFRITILPQ

QYLRPVEDVATSQDDCYKFAISQSSTGTVMGAVIMEGFYVVFDRARKRIGFAVSACHVHD

EFRTAAVEGPYLHSNMEDCGYNIPQTDESTLMTIAYVMAAICALFMLPLCLMVFQWRCFR

CLRRDHDDFADDISLLK

>Chry_pict_BACE2

FLAMVENLQGDSGRGYYLEMLIGTPPQKLNILVDTGSSNFAVAGAPNPDVTSYFNTERSS

TYKSQGTDVTVRYTQGSWTGVLGTDIITIPKGINGTYTINIATILESENFFLPGVKWHGI

LGLAYDALAKPSSSVETFFDSLVSQAKIPNVFSMLQMCGAGMPVSGSGTNGGSLVLGGIE

PSLYKGDIWYTPIKEEWYYQIEILKLEIGGQSLNLDCREYNADKAIVDSGTTLLRLPQKV

FNAVVEAIVSTSLVIQAFSSGFWTGSQLACWAKTETPWSLFPKLSIYLRDENSSRSFRIT

ILPQLYIQPILGIGQNLECYRFGISSSSSALVIGATVMEGFYVIFDRAQKRVGFAVSPCA

VSEIEGPFTTADIASNCVATMTLHEPMLWIISYALMSLCGIILLVLIIllllPPRCQHRF

TDNDVVNDESSLVHHRWK

**Section 3**

Protein sequence distance matrix generated by BIONJ in PHYLIP format. Each block represents the protein sequence similarity with each of the other sequences in the matrix.

91

Sc.japo 0.000000 0.434171 1.043019 1.506509 1.806952 1.658993

2.010460 2.022281 2.062771 2.093089 2.050186 2.017993 2.289093

2.131841 1.546626 1.553691 1.546626 1.546626 1.554495 1.538437

1.542628 1.556277 1.611561 1.545577 1.549566 1.454260 1.749932

1.519732 1.560326 1.558189 1.628806 1.514658 1.505534 1.505781

1.535341 1.491238 1.444341 1.518395 1.663238 1.532994 1.372532

1.525483 1.641839 1.565797 1.664728 1.545932 1.552856 1.657845

1.575275 1.687648 1.533079 1.669804 1.528366 1.732018 1.589978

1.593332 1.613040 1.838675 1.602837 1.683221 1.993668 1.581537

1.637437 1.628853 1.578803 1.645475 1.638852 1.671997 1.362062

1.549205 1.385979 1.643834 1.430833 1.587157 1.629472 1.886854

1.666573 1.540809 1.454586 1.329414 1.336382 1.453909 1.446769

1.456271 1.574071 1.758595 1.749677 1.601640 1.528601 1.754986

1.754986

Sc.mans 0.434171 0.000000 0.977946 1.574966 1.508813 1.736644

1.788744 2.091747 2.091963 2.091576 2.002106 2.088942 2.069729

1.999258 1.516910 1.528177 1.516910 1.516910 1.538148 1.533386

1.592399 1.535832 1.503929 1.500543 1.513095 1.282169 1.451284

1.458366 1.407671 1.500133 1.539728 1.476908 1.469320 1.455320

1.482188 1.441111 1.355390 1.503659 1.841050 1.627397 1.417287

1.497123 1.559005 1.531998 1.549466 1.573388 1.567752 1.595276

1.505204 1.618952 1.438069 1.589136 1.515671 1.647977 1.541594

1.574087 1.582216 1.815530 1.669884 1.642846 1.879921 1.589072

1.626157 1.659017 1.602337 1.669200 1.607946 1.725090 1.267995

1.353647 1.244088 1.905711 1.494717 1.492499 1.463865 1.615868

1.487520 1.446022 1.327809 1.157809 1.140828 1.218909 1.346488

1.311145 1.379494 1.613297 1.649315 1.534614 1.487652 1.728221

1.728221

Clo.sin 1.043019 0.977946 0.000000 1.387638 1.602562 1.879490

1.806599 1.893870 2.054637 1.821309 2.020219 2.123788 2.040031

2.050280 1.245303 1.252069 1.245303 1.245303 1.262504 1.262504

1.321549 1.271075 1.246958 1.245263 1.250062 1.189193 1.300473

1.231257 1.222110 1.266578 1.252344 1.256075 1.256075 1.253061

1.252111 1.260855 1.242742 1.218575 1.390428 1.438994 1.195566

1.432787 1.418659 1.432182 1.405877 1.438443 1.410189 1.412098

1.515219 1.473497 1.357040 1.440801 1.399690 1.433326 1.489891

1.468726 1.476458 1.460497 1.429652 1.488126 1.599311 1.404069

1.383224 1.520188 1.426461 1.416617 1.395233 1.556075 1.170904

1.170967 1.190832 2.267019 1.347475 1.363472 1.369731 1.384795

1.389978 1.343951 1.172273 1.152824 1.218049 1.216350 1.254215

1.280926 1.232505 1.514081 1.589418 1.338761 1.416550 1.640797

1.640797

Pl.schm 1.506509 1.574966 1.387638 0.000000 1.457904 1.582123

1.900241 1.854854 1.927337 1.855162 2.017800 2.515682 1.979931

1.926260 1.151038 1.151123 1.151038 1.151038 1.146656 1.135996

1.196013 1.147043 1.167579 1.173293 1.187656 1.234421 1.302940

1.161235 1.335938 1.209210 1.209110 1.195349 1.194347 1.152426

1.194994 1.161656 1.217640 1.198063 1.437837 1.267520 1.235412

1.374785 1.467291 1.375840 1.471150 1.398114 1.394086 1.417040

1.407925 1.425165 1.318408 1.425904 1.315965 1.342536 1.344096

1.351480 1.357746 1.445851 1.362494 1.379254 1.632696 1.304017

1.368154 1.372170 1.293920 1.354793 1.385947 1.269110 1.383329

1.386471 1.343478 1.567862 1.390667 1.448968 1.465599 1.590934

1.377137 1.443902 1.442767 1.458773 1.429669 1.448341 1.219900

1.438448 1.400030 1.428297 1.465514 1.519590 1.377130 1.629985

1.629985

Tr.adhe 1.806952 1.508813 1.602562 1.457904 0.000000 1.346108

1.337856 1.593227 1.633311 1.624246 1.648003 1.988585 1.693670

1.690328 1.225789 1.224624 1.225789 1.225789 1.217427 1.215123

1.217867 1.232312 1.182338 1.191380 1.215723 1.182610 1.205504

1.188415 1.210969 1.260618 1.276451 1.254123 1.253012 1.264062

1.248004 1.214682 1.311969 1.195947 1.325964 1.190513 1.257717

1.189248 1.158249 1.155434 1.160015 1.207899 1.200769 1.110607

1.273565 1.100456 1.064499 1.114790 1.118908 1.165948 1.223796

1.198687 1.242398 1.303566 1.236866 1.168919 1.413023 1.192865

1.211134 1.243600 1.224042 1.218158 1.185803 1.238466 1.258066

1.373851 1.228934 1.511309 1.305590 1.261056 1.295415 1.386608

1.250880 1.317640 1.284177 1.118986 1.279919 1.140927 1.222259

1.376643 1.230499 1.402399 1.544230 1.379022 1.031994 1.269503

1.269503

Mon.ovat 1.658993 1.736644 1.879490 1.582123 1.346108 0.000000

1.117029 1.683494 1.596498 1.617038 1.637163 2.030644 1.636583

1.709364 1.243690 1.244989 1.243690 1.243690 1.258828 1.251699

1.259776 1.229484 1.221511 1.230783 1.220384 1.225413 1.278503

1.255847 1.187125 1.315829 1.281967 1.290897 1.299964 1.243114

1.277077 1.278458 1.270411 1.232336 1.282632 1.182745 1.255005

1.264341 1.285475 1.258389 1.319597 1.319818 1.306218 1.224691

1.345769 1.279564 1.235155 1.223529 1.190055 1.260040 1.175702

1.220043 1.202965 1.377922 1.209554 1.276591 1.467369 1.265015

1.208969 1.271458 1.233152 1.265477 1.264193 1.263386 1.311442

1.320379 1.353964 1.496852 1.320599 1.275626 1.390582 1.458682

1.281741 1.324165 1.279877 1.363411 1.284069 1.379295 1.122629

1.407408 1.461408 1.362687 1.402366 1.376140 1.348810 1.574753

1.574753

MonoB 2.010460 1.788744 1.806599 1.900241 1.337856 1.117029

0.000000 1.680229 1.765262 1.712769 1.791871 2.064492 1.733832

1.748969 1.274116 1.269936 1.274116 1.274116 1.268923 1.266040

1.339714 1.249519 1.217579 1.233775 1.249815 1.254202 1.300144

1.254144 1.261319 1.291080 1.299833 1.302962 1.311936 1.270944

1.288486 1.280179 1.295395 1.230190 1.355331 1.320719 1.227154

1.257303 1.242227 1.206159 1.280704 1.269769 1.250418 1.285689

1.371414 1.308879 1.256108 1.237643 1.240458 1.279716 1.319607

1.326215 1.329205 1.288158 1.281756 1.271535 1.408080 1.211602

1.174972 1.240186 1.247200 1.276907 1.232339 1.318092 1.257665

1.347914 1.435917 1.637506 1.403216 1.293781 1.369589 1.391965

1.281247 1.275001 1.364424 1.375660 1.349873 1.296607 1.223670

1.471632 1.358513 1.461246 1.460015 1.429797 1.297496 1.546710

1.546710

Pl.sc.cath 2.022281 2.091747 1.893870 1.854854 1.593227 1.683494

1.680229 0.000000 0.766283 0.773043 0.620199 1.052593 0.970961

0.958370 1.667581 1.669680 1.667581 1.667581 1.653429 1.648801

1.608129 1.677537 1.674556 1.713375 1.667503 1.850743 1.916234

1.620406 1.676629 1.663489 1.764600 1.710738 1.708863 1.639401

1.671244 1.630143 1.567447 1.619050 1.784582 1.628463 1.681671

1.611061 1.652851 1.610502 1.646864 1.595421 1.592964 1.670587

1.698887 1.603461 1.625618 1.543639 1.543105 1.577295 1.664708

1.666147 1.638304 1.727274 1.637889 1.660652 1.787569 1.626017

1.661665 1.593823 1.647189 1.586842 1.633786 1.563905 1.700440

1.763883 1.821251 1.631547 1.743919 1.727516 1.782040 1.850140

1.714951 1.670158 1.768409 1.760257 1.682576 1.916012 1.752510

1.743396 1.635906 1.806717 1.996165 1.945225 1.549109 1.794823

1.794823

Mon.o.cath 2.062771 2.091963 2.054637 1.927337 1.633311 1.596498

1.765262 0.766283 0.000000 0.596745 0.609762 0.859235 0.739295

0.749368 1.816937 1.831218 1.816937 1.816937 1.809038 1.805738

1.833440 1.849661 1.787906 1.776564 1.801416 1.873572 1.952646

1.809567 1.853028 1.800793 1.824740 1.789772 1.789741 1.729563

1.759485 1.724943 1.652658 1.799487 1.944405 1.743270 1.663810

1.700559 1.761645 1.716866 1.778072 1.730609 1.720713 1.745246

1.687919 1.687588 1.636589 1.686258 1.628924 1.791252 1.588505

1.592159 1.594669 1.763606 1.695790 1.676658 1.741554 1.610342

1.700076 1.591453 1.673878 1.586661 1.660704 1.650518 1.647190

1.779606 1.882471 1.921877 1.900050 1.969919 1.998080 2.039728

1.927043 1.926207 1.786073 1.818214 1.774506 1.620712 1.821756

1.720346 1.759020 1.777293 1.931694 1.744977 1.651417 1.738978

1.738978

St.pu.cat0 2.093089 2.091576 1.821309 1.855162 1.624246 1.617038

1.712769 0.773043 0.596745 0.000000 0.709225 1.080352 0.928051

0.919985 1.911877 1.891132 1.911877 1.911877 1.911877 1.907154

1.821773 1.877249 1.815557 1.888127 1.896038 1.886169 2.087863

1.886915 1.856137 1.906834 1.972184 1.935364 1.935200 1.878300

1.913057 1.889796 1.699037 1.910457 2.074020 1.692733 1.698876

1.618782 1.653468 1.596113 1.642879 1.695377 1.685809 1.645685

1.759772 1.635090 1.676114 1.550969 1.580353 1.605304 1.657126

1.618183 1.653044 1.645568 1.648237 1.580027 1.668232 1.574258

1.630772 1.609512 1.631242 1.592745 1.678955 1.752318 1.765052

1.763414 1.894535 1.888651 1.914183 1.771864 1.803676 1.820351

1.845866 1.802540 1.697787 1.768015 1.862480 1.773494 1.877079

1.694608 1.871451 1.802603 1.729734 1.972724 1.806161 1.671900

1.671900

Na.v.cath0 2.050186 2.002106 2.020219 2.017800 1.648003 1.637163

1.791871 0.620199 0.609762 0.709225 0.000000 0.660430 0.749607

0.745579 1.824645 1.820426 1.824645 1.824645 1.800205 1.805226

1.797101 1.830601 1.796313 1.834183 1.827814 1.853626 1.951058

1.740650 1.761550 1.789790 1.853564 1.791720 1.789703 1.750466

1.766506 1.770839 1.691268 1.787547 1.891263 1.702571 1.854728

1.728026 1.796572 1.740201 1.813091 1.780130 1.770298 1.903774

1.817074 1.760684 1.708309 1.697751 1.661555 1.755088 1.640568

1.649721 1.680689 1.835564 1.747098 1.742755 1.837081 1.755818

1.754933 1.704784 1.715994 1.606928 1.696146 1.750957 1.744823

1.873697 1.818540 1.792482 2.034847 1.967418 2.008548 2.091880

1.987710 1.917276 1.857556 2.053069 1.935135 1.958087 1.935348

1.838496 1.847921 1.891125 2.073870 1.919048 1.827079 1.990165

1.990165

Br.f.cath0 2.017993 2.088942 2.123788 2.515682 1.988585 2.030644

2.064492 1.052593 0.859235 1.080352 0.660430 0.000000 0.998279

1.063425 2.208530 2.230977 2.208530 2.208530 2.191469 2.183322

2.252965 2.184643 2.253936 2.245422 2.244673 1.821237 2.038922

2.087370 1.997669 2.119712 2.180325 2.119765 2.112363 2.033218

2.067629 2.046128 1.942650 2.148762 2.386838 2.019057 2.254700

2.107503 2.210395 2.180519 2.237333 2.159042 2.183904 2.284561

2.146297 2.262046 2.236655 2.143862 2.039395 2.274916 2.096065

2.079128 2.080535 1.873492 2.200490 2.181090 2.604304 2.148171

2.213390 2.180346 2.082841 2.050414 2.131531 2.161561 2.114041

2.547891 2.148348 2.228688 2.289704 2.445910 2.577355 2.711795

2.545071 2.381806 2.238450 2.088164 1.966682 2.059615 2.345588

2.201145 2.209419 2.320104 2.504390 2.350305 2.329973 2.406692

2.406692

Ci.i.cath0 2.289093 2.069729 2.040031 1.979931 1.693670 1.636583

1.733832 0.970961 0.739295 0.928051 0.749607 0.998279 0.000000

0.228749 1.660727 1.652165 1.660727 1.660727 1.674029 1.678579

1.607809 1.612696 1.656536 1.667659 1.668148 1.773582 1.900575

1.651735 1.816508 1.668818 1.683154 1.702756 1.691008 1.614364

1.655345 1.656067 1.647865 1.656717 1.801860 1.605009 1.776325

1.641644 1.676976 1.657116 1.683609 1.655239 1.645395 1.734617

1.673743 1.743064 1.627767 1.655321 1.637711 1.692428 1.667654

1.681155 1.658193 1.553255 1.652800 1.635259 1.883725 1.708496

1.694271 1.681248 1.639799 1.610257 1.659853 1.579199 1.717041

1.929020 1.940041 1.775148 1.919427 1.844547 1.856447 1.807197

1.776147 1.841210 1.815940 2.045762 1.937597 1.902647 1.834556

1.840917 1.772181 1.787279 1.873965 2.012422 1.824381 1.990751

1.990751

Ci.sau.cat 2.131841 1.999258 2.050280 1.926260 1.690328 1.709364

1.748969 0.958370 0.749368 0.919985 0.745579 1.063425 0.228749

0.000000 1.767374 1.787385 1.767374 1.767374 1.784133 1.773050

1.751856 1.800952 1.776045 1.792445 1.799396 1.949949 2.090362

1.777587 1.982425 1.739595 1.738166 1.750782 1.750409 1.669269

1.704912 1.699869 1.714587 1.826625 1.959759 1.697062 1.750426

1.708791 1.743588 1.716192 1.763412 1.729975 1.738429 1.778627

1.723020 1.837810 1.701040 1.751928 1.718684 1.784736 1.718918

1.730997 1.702337 1.622929 1.749646 1.681107 1.909160 1.744668

1.768197 1.718503 1.682391 1.606497 1.711966 1.698643 1.761424

1.938440 1.908567 1.702450 1.940611 1.836027 1.864450 1.835521

1.862428 1.868235 1.796884 1.944322 1.818929 1.868874 1.805278

1.766773 1.907500 1.979341 1.966915 2.089627 1.847608 1.956686

1.956686

Hu.BACE1 1.546626 1.516910 1.245303 1.151038 1.225789 1.243690

1.274116 1.667581 1.816937 1.911877 1.824645 2.208530 1.660727

1.767374 0.000000 0.018445 0.024695 0.018501 0.037215 0.037233

0.050314 0.082376 0.084824 0.111589 0.084101 0.131554 0.157557

0.115130 0.120502 0.244412 0.183679 0.201695 0.224462 0.201594

0.185183 0.190770 0.226392 0.195070 0.284346 0.367261 0.579381

0.745817 0.785800 0.778567 0.800701 0.767760 0.773086 0.753068

0.793997 0.779380 0.765373 0.758906 0.700365 0.777382 0.845614

0.841803 0.865411 1.013638 0.716318 0.734931 0.756209 0.734862

0.759773 0.726342 0.703618 0.727928 0.654277 0.652961 0.864379

0.660573 0.800388 1.235625 0.857405 0.830244 0.841640 0.891524

0.877743 0.913450 0.861988 0.919016 0.905956 0.747777 0.787292

0.919971 0.983301 1.182554 1.060704 1.126508 0.935221 1.423375

1.423375

Bo.BACE1 1.553691 1.528177 1.252069 1.151123 1.224624 1.244989

1.269936 1.669680 1.831218 1.891132 1.820426 2.230977 1.652165

1.787385 0.018445 0.000000 0.030953 0.022627 0.035055 0.039263

0.047833 0.077781 0.084384 0.109111 0.078961 0.129927 0.155058

0.115002 0.116699 0.250704 0.186923 0.204431 0.223607 0.198650

0.184590 0.191221 0.222906 0.197257 0.275059 0.359561 0.577330

0.746910 0.785428 0.780646 0.794408 0.770601 0.775954 0.758362

0.795330 0.777332 0.768833 0.756425 0.699734 0.774804 0.845648

0.841836 0.865089 1.006071 0.716676 0.735647 0.761147 0.739504

0.754372 0.724990 0.702628 0.727607 0.659739 0.653049 0.864401

0.650528 0.811198 1.243413 0.865827 0.821828 0.835974 0.888358

0.871580 0.908752 0.863854 0.920179 0.909582 0.760621 0.802896

0.921500 0.982612 1.181058 1.055884 1.124240 0.942676 1.428856

1.428856

Do.BACE1 1.546626 1.516910 1.245303 1.151038 1.225789 1.243690

1.274116 1.667581 1.816937 1.911877 1.824645 2.208530 1.660727

1.767374 0.024695 0.030953 0.000000 0.016385 0.041527 0.041547

0.047713 0.080144 0.084765 0.107357 0.084044 0.131554 0.157557

0.115053 0.120858 0.240314 0.183679 0.201010 0.220847 0.195693

0.181846 0.187837 0.222804 0.197576 0.287933 0.370409 0.580151

0.745442 0.786314 0.779063 0.795299 0.769083 0.774421 0.753677

0.793579 0.777503 0.763271 0.757068 0.698729 0.775485 0.848074

0.844247 0.867899 1.008615 0.715734 0.734892 0.756209 0.734826

0.759746 0.726302 0.703570 0.727890 0.654277 0.652961 0.862186

0.660573 0.800388 1.235625 0.857906 0.829855 0.843909 0.891552

0.872572 0.913972 0.856695 0.921542 0.911591 0.751162 0.787292

0.917704 0.984831 1.177514 1.055749 1.121388 0.935221 1.423375

1.423375

Ail1 1.546626 1.516910 1.245303 1.151038 1.225789 1.243690

1.274116 1.667581 1.816937 1.911877 1.824645 2.208530 1.660727

1.767374 0.018501 0.022627 0.016385 0.000000 0.041380 0.041400

0.047596 0.080026 0.084685 0.109351 0.083968 0.131554 0.157557

0.114780 0.120697 0.248791 0.183679 0.201624 0.221646 0.196692

0.182618 0.188421 0.222650 0.194774 0.284183 0.366785 0.579259

0.745126 0.785936 0.778698 0.794927 0.768748 0.774081 0.753325

0.793213 0.775726 0.762871 0.754857 0.696810 0.773185 0.846387

0.842579 0.866149 1.008482 0.715440 0.732675 0.756209 0.732746

0.757595 0.724250 0.701596 0.726543 0.654277 0.652961 0.861780

0.660573 0.800388 1.235625 0.856277 0.829855 0.843909 0.891552

0.869992 0.911397 0.856695 0.918328 0.911613 0.751162 0.787292

0.915798 0.984816 1.177514 1.055749 1.121388 0.935221 1.423375

1.423375

Mou.BACE1 1.554495 1.538148 1.262504 1.146656 1.217427 1.258828

1.268923 1.653429 1.809038 1.911877 1.800205 2.191469 1.674029

1.784133 0.037215 0.035055 0.041527 0.041380 0.000000 0.006091

0.066454 0.087033 0.084314 0.109219 0.086299 0.141956 0.155603

0.114528 0.119788 0.255174 0.190321 0.200894 0.223619 0.203529

0.187333 0.187406 0.221497 0.196518 0.283388 0.362119 0.579184

0.746007 0.786594 0.779368 0.791543 0.765641 0.770942 0.738657

0.779702 0.774261 0.760780 0.749228 0.695613 0.756661 0.841873

0.834053 0.853377 1.012303 0.706404 0.718382 0.735228 0.707034

0.736040 0.703022 0.676594 0.711209 0.643081 0.644809 0.848469

0.634239 0.806287 1.235625 0.857657 0.841774 0.855578 0.905348

0.875784 0.914159 0.866432 0.923279 0.915834 0.758096 0.787374

0.917874 0.972086 1.176561 1.067017 1.136336 0.924959 1.422968

1.422968

Ra.BACE1 1.538437 1.533386 1.262504 1.135996 1.215123 1.251699

1.266040 1.648801 1.805738 1.907154 1.805226 2.183322 1.678579

1.773050 0.037233 0.039263 0.041547 0.041400 0.006091 0.000000

0.068782 0.089320 0.086804 0.111560 0.088707 0.141956 0.155603

0.113943 0.119788 0.255005 0.186576 0.197891 0.220613 0.200629

0.184477 0.184432 0.221497 0.198966 0.287652 0.358001 0.574238

0.746793 0.787440 0.780196 0.792390 0.766438 0.772500 0.739485

0.776906 0.775923 0.762640 0.750844 0.696383 0.757518 0.843566

0.835730 0.852044 1.012303 0.705400 0.722973 0.742842 0.711332

0.732912 0.699586 0.673289 0.707781 0.639799 0.637472 0.845427

0.631870 0.797327 1.214875 0.851159 0.838276 0.852107 0.901053

0.874387 0.910421 0.865132 0.923279 0.915834 0.758096 0.781212

0.919626 0.973931 1.170791 1.058574 1.137316 0.919867 1.423972

1.423972

Mon.d.BACE 1.542628 1.592399 1.321549 1.196013 1.217867 1.259776

1.339714 1.608129 1.833440 1.821773 1.797101 2.252965 1.607809

1.751856 0.050314 0.047833 0.047713 0.047596 0.066454 0.068782

0.000000 0.082553 0.095694 0.116996 0.091992 0.167797 0.193467

0.120324 0.139052 0.260317 0.195665 0.213711 0.229735 0.208948

0.201358 0.206791 0.249688 0.197798 0.270094 0.358152 0.585667

0.791117 0.837922 0.828631 0.852143 0.811591 0.817749 0.805109

0.824263 0.812518 0.812903 0.789194 0.729818 0.815644 0.892202

0.887678 0.912917 1.161863 0.740328 0.767398 0.816009 0.767338

0.777954 0.755523 0.726534 0.756201 0.679397 0.660435 0.908373

0.678418 0.846401 1.246729 0.895440 0.840092 0.856908 0.918481

0.885170 0.951864 0.909014 0.976971 1.000223 0.802117 0.857566

0.932962 1.040551 1.253308 1.105402 1.213687 0.968493 1.416023

1.416023

Ori.an.BAC 1.556277 1.535832 1.271075 1.147043 1.232312 1.229484

1.249519 1.677537 1.849661 1.877249 1.830601 2.184643 1.612696

1.800952 0.082376 0.077781 0.080144 0.080026 0.087033 0.089320

0.082553 0.000000 0.092835 0.122059 0.099394 0.132866 0.144054

0.120385 0.121036 0.254896 0.192882 0.200830 0.228481 0.204136

0.190794 0.194752 0.231489 0.206072 0.299202 0.355984 0.588554

0.752291 0.796354 0.788849 0.813484 0.775271 0.780669 0.768171

0.786006 0.782286 0.758920 0.759970 0.701485 0.753937 0.854111

0.846626 0.867462 1.026017 0.701610 0.720607 0.736435 0.730581

0.736352 0.717387 0.695687 0.712288 0.651767 0.644006 0.834875

0.620184 0.818686 1.303395 0.847122 0.815295 0.831521 0.864746

0.860799 0.908690 0.893894 0.953586 0.890003 0.769289 0.757587

0.914313 1.001070 1.186898 1.056676 1.130640 0.966970 1.438032

1.438032

Gl.G.BACE1 1.611561 1.503929 1.246958 1.167579 1.182338 1.221511

1.217579 1.674556 1.787906 1.815557 1.796313 2.253936 1.656536

1.776045 0.084824 0.084384 0.084765 0.084685 0.084314 0.086804

0.095694 0.092835 0.000000 0.019127 0.043697 0.108900 0.114953

0.102250 0.098121 0.234054 0.198642 0.221579 0.221458 0.194982

0.194669 0.204559 0.234034 0.156103 0.248668 0.373088 0.571177

0.767727 0.793449 0.782738 0.805623 0.786131 0.791837 0.733154

0.787435 0.764142 0.741587 0.729214 0.693941 0.758366 0.864030

0.853632 0.870327 1.042600 0.700123 0.708940 0.703617 0.729114

0.760886 0.705979 0.688196 0.708695 0.641200 0.624783 0.848542

0.641270 0.797733 1.284583 0.847419 0.847079 0.854856 0.886984

0.878789 0.927601 0.849070 0.911238 0.889988 0.756684 0.718418

0.894584 0.983358 1.180005 1.098261 1.110168 0.977192 1.393100

1.393100

Tae.gu.BAC 1.545577 1.500543 1.245263 1.173293 1.191380 1.230783

1.233775 1.713375 1.776564 1.888127 1.834183 2.245422 1.667659

1.792445 0.111589 0.109111 0.107357 0.109351 0.109219 0.111560

0.116996 0.122059 0.019127 0.000000 0.049237 0.108638 0.125130

0.101267 0.098352 0.274543 0.196407 0.220567 0.245856 0.227514

0.212689 0.197101 0.238816 0.212062 0.366500 0.365778 0.550568

0.754375 0.795075 0.781906 0.807215 0.770327 0.775652 0.727709

0.770897 0.771388 0.749781 0.736371 0.691994 0.763835 0.848691

0.839426 0.860135 1.032633 0.700644 0.712506 0.709936 0.715361

0.732578 0.706114 0.682309 0.700062 0.633337 0.633581 0.838658

0.616057 0.780196 1.217152 0.824016 0.828291 0.838300 0.883557

0.875448 0.910357 0.856439 0.909456 0.891275 0.758606 0.755010

0.891685 0.977070 1.172772 1.086580 1.121152 0.978723 1.398805

1.398805

Ch 1.549566 1.513095 1.250062 1.187656 1.215723 1.220384

1.249815 1.667503 1.801416 1.896038 1.827814 2.244673 1.668148

1.799396 0.084101 0.078961 0.084044 0.083968 0.086299 0.088707

0.091992 0.099394 0.043697 0.049237 0.000000 0.113866 0.142850

0.106404 0.097380 0.234867 0.204513 0.224122 0.224004 0.202449

0.198454 0.205652 0.239649 0.142528 0.209899 0.367963 0.568422

0.774080 0.807961 0.799719 0.826186 0.787598 0.793096 0.764551

0.802380 0.793522 0.784021 0.765802 0.717941 0.793394 0.875369

0.878788 0.904720 1.061163 0.704207 0.730114 0.727797 0.764615

0.772385 0.743567 0.710222 0.723637 0.675874 0.673810 0.863534

0.650553 0.806812 1.209208 0.836507 0.836609 0.849293 0.898356

0.880423 0.925143 0.867802 0.915189 0.882803 0.755623 0.777008

0.891483 0.978569 1.175605 1.074323 1.094048 0.963168 1.397962

1.397962

Le.e.BACEi 1.454260 1.282169 1.189193 1.234421 1.182610 1.225413

1.254202 1.850743 1.873572 1.886169 1.853626 1.821237 1.773582

1.949949 0.131554 0.129927 0.131554 0.131554 0.141956 0.141956

0.167797 0.132866 0.108900 0.108638 0.113866 0.000000 0.036625

0.105652 0.125595 0.188803 0.206134 0.215768 0.215768 0.183853

0.187855 0.185311 0.179134 0.134320 0.359937 0.342130 0.683275

0.688568 0.667372 0.692457 0.707743 0.683577 0.691640 0.663894

0.735214 0.729808 0.668554 0.695436 0.661288 0.694031 0.818034

0.828527 0.837012 0.892738 0.714222 0.723314 0.814748 0.783191

0.809537 0.779629 0.742885 0.724001 0.693673 0.663095 0.808667

0.824590 0.720843 2.277533 0.877075 0.865669 0.864298 0.831099

0.919993 0.890175 0.861331 0.826529 0.769380 0.713682 0.775299

0.947183 0.870587 1.075586 1.043888 1.206328 1.032253 1.450973

1.450973

Sq.aca.BAC 1.749932 1.451284 1.300473 1.302940 1.205504 1.278503

1.300144 1.916234 1.952646 2.087863 1.951058 2.038922 1.900575

2.090362 0.157557 0.155058 0.157557 0.157557 0.155603 0.155603

0.193467 0.144054 0.114953 0.125130 0.142850 0.036625 0.000000

0.094541 0.149298 0.242392 0.216016 0.245702 0.245702 0.220857

0.217641 0.200938 0.200938 0.159902 0.406001 0.336469 0.711364

0.676204 0.673790 0.692172 0.695384 0.684840 0.689614 0.634050

0.698872 0.707956 0.642219 0.687895 0.703211 0.662189 0.826745

0.836645 0.835360 0.991439 0.709786 0.710784 0.715754 0.739400

0.798327 0.750624 0.713907 0.714921 0.700032 0.635236 0.775009

0.694432 0.753530 2.495439 0.965571 1.049441 0.984835 0.978763

1.074047 1.042270 0.940370 0.845927 0.852917 0.794432 0.782055

0.968831 0.971030 1.265517 1.264096 1.291022 1.172536 1.691218

1.691218

La.cha.BAC 1.519732 1.458366 1.231257 1.161235 1.188415 1.255847

1.254144 1.620406 1.809567 1.886915 1.740650 2.087370 1.651735

1.777587 0.115130 0.115002 0.115053 0.114780 0.114528 0.113943

0.120324 0.120385 0.102250 0.101267 0.106404 0.105652 0.094541

0.000000 0.124492 0.227043 0.198106 0.218403 0.215475 0.192825

0.189921 0.201861 0.238764 0.159770 0.292314 0.389277 0.577633

0.743019 0.789783 0.777079 0.792327 0.760230 0.765644 0.751510

0.774122 0.759400 0.739557 0.742134 0.685698 0.766149 0.839346

0.832390 0.851960 1.029133 0.712359 0.742688 0.764832 0.715582

0.730778 0.709746 0.651903 0.690383 0.630968 0.650138 0.822904

0.616131 0.779726 1.164368 0.863388 0.820108 0.842545 0.868881

0.871740 0.894399 0.872290 0.942147 0.927240 0.763323 0.773147

0.901452 0.973071 1.184944 1.127299 1.138111 0.989571 1.428428

1.428428

An.ca.BACE 1.560326 1.407671 1.222110 1.335938 1.210969 1.187125

1.261319 1.676629 1.853028 1.856137 1.761550 1.997669 1.816508

1.982425 0.120502 0.116699 0.120858 0.120697 0.119788 0.119788

0.139052 0.121036 0.098121 0.098352 0.097380 0.125595 0.149298

0.124492 0.000000 0.252938 0.227353 0.256926 0.256926 0.211202

0.214163 0.230822 0.221905 0.184875 0.287964 0.425592 0.708613

0.817278 0.809125 0.824411 0.826094 0.839664 0.846854 0.771626

0.845453 0.785796 0.773919 0.790398 0.744481 0.791584 0.938926

0.944666 0.941352 1.052710 0.776229 0.738541 0.704776 0.818703

0.841960 0.813292 0.776717 0.774238 0.674947 0.666740 0.967701

0.720600 0.762100 2.354950 1.021705 0.993813 0.999516 0.987294

1.055080 1.027895 0.965090 0.924023 0.892477 0.767862 0.872237

0.982398 1.052412 1.370495 1.293571 1.340801 1.131110 1.507761

1.507761

Da1 1.558189 1.500133 1.266578 1.209210 1.260618 1.315829

1.291080 1.663489 1.800793 1.906834 1.789790 2.119712 1.668818

1.739595 0.244412 0.250704 0.240314 0.248791 0.255174 0.255005

0.260317 0.254896 0.234054 0.274543 0.234867 0.188803 0.242392

0.227043 0.252938 0.000000 0.084539 0.108120 0.126225 0.117906

0.102956 0.111005 0.138780 0.304650 0.489957 0.444246 0.602216

0.831087 0.853052 0.843399 0.862154 0.834103 0.839888 0.843047

0.864534 0.839143 0.830422 0.800450 0.761994 0.818270 0.879404

0.884886 0.915822 1.032145 0.744503 0.711019 0.715524 0.733471

0.744925 0.733977 0.676858 0.692851 0.630152 0.693341 0.824680

0.644116 0.754143 1.216120 0.898949 0.821252 0.840766 0.876066

0.888645 0.909058 0.890892 0.918647 0.918933 0.775233 0.727549

0.936952 0.949586 1.230980 1.126227 1.179762 0.980011 1.489034

1.489034

Sal.salm.B 1.628806 1.539728 1.252344 1.209110 1.276451 1.281967

1.299833 1.764600 1.824740 1.972184 1.853564 2.180325 1.683154

1.738166 0.183679 0.186923 0.183679 0.183679 0.190321 0.186576

0.195665 0.192882 0.198642 0.196407 0.204513 0.206134 0.216016

0.198106 0.227353 0.084539 0.000000 0.050378 0.050395 0.041273

0.038408 0.056802 0.105218 0.205207 0.384117 0.324660 0.540273

0.721550 0.745800 0.741989 0.772485 0.728476 0.733272 0.739672

0.760077 0.768353 0.744603 0.744731 0.708613 0.731348 0.806432

0.799764 0.818829 0.891740 0.658334 0.661794 0.701753 0.653694

0.658772 0.662302 0.627133 0.630101 0.623732 0.651038 0.690106

0.632459 0.757213 1.214675 0.788740 0.766481 0.752529 0.797769

0.836681 0.876735 0.851007 0.785370 0.864668 0.723101 0.771477

0.911853 0.887745 1.140508 1.016309 1.094942 1.000250 1.529039

1.529039

Ory1 1.514658 1.476908 1.256075 1.195349 1.254123 1.290897

1.302962 1.710738 1.789772 1.935364 1.791720 2.119765 1.702756

1.750782 0.201695 0.204431 0.201010 0.201624 0.200894 0.197891

0.213711 0.200830 0.221579 0.220567 0.224122 0.215768 0.245702

0.218403 0.256926 0.108120 0.050378 0.000000 0.004548 0.058427

0.046554 0.070572 0.115922 0.230363 0.381701 0.440877 0.620621

0.835356 0.872294 0.861913 0.886035 0.834249 0.840890 0.865196

0.857617 0.852964 0.845666 0.820044 0.762208 0.831955 0.898121

0.889236 0.920149 1.055818 0.733622 0.704659 0.691354 0.728458

0.736715 0.732941 0.685008 0.699827 0.631374 0.689577 0.828217

0.614483 0.745666 1.242272 0.868401 0.818057 0.831788 0.874448

0.892276 0.923502 0.911464 0.914079 0.912278 0.779786 0.776294

0.944919 0.991268 1.215337 1.078213 1.165725 0.959391 1.480710

1.480710

Gd1 1.505534 1.469320 1.256075 1.194347 1.253012 1.299964

1.311936 1.708863 1.789741 1.935200 1.789703 2.112363 1.691008

1.750409 0.224462 0.223607 0.220847 0.221646 0.223619 0.220613

0.229735 0.228481 0.221458 0.245856 0.224004 0.215768 0.245702

0.215475 0.256926 0.126225 0.050395 0.004548 0.000000 0.081056

0.064503 0.072988 0.115922 0.261520 0.450267 0.440608 0.620253

0.834819 0.871700 0.861337 0.885431 0.833713 0.840348 0.864604

0.857067 0.852376 0.844999 0.819481 0.761718 0.831372 0.897525

0.888652 0.918842 1.055818 0.738375 0.709753 0.691354 0.727982

0.736228 0.732461 0.689683 0.699369 0.630935 0.689002 0.822806

0.611880 0.745002 1.240298 0.867859 0.823149 0.831615 0.874448

0.891663 0.922904 0.911979 0.914079 0.912278 0.779786 0.775468

0.944320 0.987991 1.214628 1.077456 1.160714 0.958649 1.479522

1.479522

Gs.acu.BAC 1.505781 1.455320 1.253061 1.152426 1.264062 1.243114

1.270944 1.639401 1.729563 1.878300 1.750466 2.033218 1.614364

1.669269 0.201594 0.198650 0.195693 0.196692 0.203529 0.200629

0.208948 0.204136 0.194982 0.227514 0.202449 0.183853 0.220857

0.192825 0.211202 0.117906 0.041273 0.058427 0.081056 0.000000

0.034623 0.042138 0.075818 0.277634 0.453456 0.402740 0.603497

0.797398 0.829197 0.819841 0.844613 0.810587 0.817053 0.823452

0.828967 0.812355 0.807992 0.789665 0.729612 0.806630 0.890940

0.884605 0.902674 1.016262 0.724445 0.706256 0.708548 0.719063

0.731794 0.724465 0.673627 0.694279 0.633243 0.668014 0.808957

0.609247 0.737337 1.250081 0.894336 0.832409 0.844342 0.867545

0.900747 0.935201 0.896959 0.943891 0.922802 0.771546 0.772303

0.941102 0.987173 1.223603 1.091379 1.169707 0.955825 1.475101

1.475101

Ore1 1.535341 1.482188 1.252111 1.194994 1.248004 1.277077

1.288486 1.671244 1.759485 1.913057 1.766506 2.067629 1.655345

1.704912 0.185183 0.184590 0.181846 0.182618 0.187333 0.184477

0.201358 0.190794 0.194669 0.212689 0.198454 0.187855 0.217641

0.189921 0.214163 0.102956 0.038408 0.046554 0.064503 0.034623

0.000000 0.042173 0.081608 0.249970 0.422202 0.412574 0.606081

0.817259 0.855481 0.845497 0.866108 0.833765 0.840416 0.844710

0.842681 0.832131 0.821627 0.805682 0.745441 0.807730 0.878997

0.872817 0.890568 1.042194 0.730195 0.709474 0.714058 0.728281

0.731083 0.730052 0.682758 0.685654 0.639622 0.688174 0.837249

0.622226 0.736909 1.207095 0.901569 0.809643 0.828653 0.854677

0.889452 0.917399 0.906467 0.940691 0.924383 0.770982 0.770588

0.941553 0.996289 1.221782 1.087831 1.149961 0.950094 1.464839

1.464839

Tak1 1.491238 1.441111 1.260855 1.161656 1.214682 1.278458

1.280179 1.630143 1.724943 1.889796 1.770839 2.046128 1.656067

1.699869 0.190770 0.191221 0.187837 0.188421 0.187406 0.184432

0.206791 0.194752 0.204559 0.197101 0.205652 0.185311 0.200938

0.201861 0.230822 0.111005 0.056802 0.070572 0.072988 0.042138

0.042173 0.000000 0.052325 0.213606 0.372767 0.419342 0.588581

0.791283 0.828577 0.819290 0.832247 0.793490 0.799779 0.802981

0.806573 0.800098 0.803775 0.767542 0.719688 0.783451 0.871169

0.860471 0.879858 1.015003 0.717463 0.694653 0.698739 0.709575

0.739896 0.721115 0.673529 0.687527 0.624829 0.676449 0.811833

0.607531 0.748379 1.262789 0.890495 0.818944 0.833213 0.866224

0.884694 0.926122 0.883498 0.937302 0.924563 0.760838 0.734981

0.927380 0.987699 1.208993 1.105936 1.142527 0.936000 1.448704

1.448704

Te1 1.444341 1.355390 1.242742 1.217640 1.311969 1.270411

1.295395 1.567447 1.652658 1.699037 1.691268 1.942650 1.647865

1.714587 0.226392 0.222906 0.222804 0.222650 0.221497 0.221497

0.249688 0.231489 0.234034 0.238816 0.239649 0.179134 0.200938

0.238764 0.221905 0.138780 0.105218 0.115922 0.115922 0.075818

0.081608 0.052325 0.000000 0.238652 0.406436 0.467094 0.678793

0.834251 0.855827 0.855036 0.862566 0.842144 0.841506 0.811921

0.863155 0.815292 0.820645 0.805252 0.773500 0.794670 0.949008

0.932930 0.937954 1.010749 0.792780 0.758824 0.704229 0.795649

0.846608 0.802860 0.759041 0.756861 0.691430 0.725880 0.888142

0.703086 0.745412 1.839839 0.968676 0.962575 0.955857 0.929039

0.977926 0.984439 0.891677 0.934541 0.914416 0.755972 0.740165

0.954089 1.032741 1.264416 1.206696 1.209930 0.993131 1.445306

1.445306

X.tr.BACE1 1.518395 1.503659 1.218575 1.198063 1.195947 1.232336

1.230190 1.619050 1.799487 1.910457 1.787547 2.148762 1.656717

1.826625 0.195070 0.197257 0.197576 0.194774 0.196518 0.198966

0.197798 0.206072 0.156103 0.212062 0.142528 0.134320 0.159902

0.159770 0.184875 0.304650 0.205207 0.230363 0.261520 0.277634

0.249970 0.213606 0.238652 0.000000 0.413030 0.393935 0.563094

0.768451 0.823618 0.805663 0.820895 0.780731 0.786175 0.793739

0.785157 0.778559 0.750682 0.755612 0.705060 0.787008 0.902804

0.881163 0.905722 1.029121 0.707130 0.736496 0.727969 0.750837

0.743016 0.728995 0.698567 0.720715 0.665607 0.661979 0.843956

0.640257 0.803108 1.223082 0.852533 0.842726 0.817910 0.873208

0.827190 0.858773 0.840094 0.974762 0.889912 0.770304 0.771969

0.889233 1.005841 1.150940 1.078107 1.117138 0.964350 1.354668

1.354668

Pel1 1.663238 1.841050 1.390428 1.437837 1.325964 1.282632

1.355331 1.784582 1.944405 2.074020 1.891263 2.386838 1.801860

1.959759 0.284346 0.275059 0.287933 0.284183 0.283388 0.287652

0.270094 0.299202 0.248668 0.366500 0.209899 0.359937 0.406001

0.292314 0.287964 0.489957 0.384117 0.381701 0.450267 0.453456

0.422202 0.372767 0.406436 0.413030 0.000000 0.479081 0.652659

0.916121 0.973922 0.949519 0.988333 0.923358 0.928470 0.914779

0.909496 0.983082 0.933635 0.962752 0.879899 0.986177 0.969423

0.990606 1.012947 0.972113 0.811991 0.868958 0.860852 0.853765

0.823240 0.834210 0.817941 0.825386 0.828545 0.996493 0.869594

0.832060 0.938593 1.188069 0.851059 0.922676 0.941218 0.950301

0.953109 1.037130 0.962881 0.998515 0.885917 0.840912 0.968361

0.922368 0.973100 1.224723 1.214778 1.168892 1.103922 1.450811

1.450811

Pet.mari 1.532994 1.627397 1.438994 1.267520 1.190513 1.182745

1.320719 1.628463 1.743270 1.692733 1.702571 2.019057 1.605009

1.697062 0.367261 0.359561 0.370409 0.366785 0.362119 0.358001

0.358152 0.355984 0.373088 0.365778 0.367963 0.342130 0.336469

0.389277 0.425592 0.444246 0.324660 0.440877 0.440608 0.402740

0.412574 0.419342 0.467094 0.393935 0.479081 0.000000 0.586526

0.823478 0.871773 0.850563 0.864639 0.849641 0.853833 0.814783

0.847642 0.899925 0.872691 0.846545 0.800388 0.842088 0.960244

0.951386 0.967918 1.130175 0.793147 0.774917 0.847789 0.784020

0.804093 0.778745 0.766931 0.768475 0.717394 0.746659 0.869768

0.704051 0.809563 1.318957 0.934907 0.855184 0.818442 0.882574

0.831121 0.860542 0.952625 1.046925 1.060694 0.841605 0.893042

1.028064 1.165886 1.227508 1.211726 1.189248 1.000163 1.421487

1.421487

Br.flor 1.372532 1.417287 1.195566 1.235412 1.257717 1.255005

1.227154 1.681671 1.663810 1.698876 1.854728 2.254700 1.776325

1.750426 0.579381 0.577330 0.580151 0.579259 0.579184 0.574238

0.585667 0.588554 0.571177 0.550568 0.568422 0.683275 0.711364

0.577633 0.708613 0.602216 0.540273 0.620621 0.620253 0.603497

0.606081 0.588581 0.678793 0.563094 0.652659 0.586526 0.000000

0.985055 0.998582 0.984847 0.991412 0.964120 0.972134 0.949319

0.939739 1.005059 0.963372 0.983225 0.941094 0.940999 0.996809

0.976677 0.972183 1.105279 0.863277 0.865334 0.926819 0.881506

0.860928 0.857807 0.838113 0.870858 0.817729 0.890150 0.842190

0.673874 0.785427 1.296552 0.795218 0.734414 0.731647 0.738212

0.723209 0.795559 0.694809 0.857035 0.793969 0.619124 0.759807

0.822916 1.080327 1.160648 1.139600 1.121601 1.040486 1.401252

1.401252

Hu.BACE2 1.525483 1.497123 1.432787 1.374785 1.189248 1.264341

1.257303 1.611061 1.700559 1.618782 1.728026 2.107503 1.641644

1.708791 0.745817 0.746910 0.745442 0.745126 0.746007 0.746793

0.791117 0.752291 0.767727 0.754375 0.774080 0.688568 0.676204

0.743019 0.817278 0.831087 0.721550 0.835356 0.834819 0.797398

0.817259 0.791283 0.834251 0.768451 0.916121 0.823478 0.985055

0.000000 0.079656 0.065549 0.082070 0.114685 0.112383 0.180480

0.364496 0.262664 0.355555 0.252163 0.205805 0.338663 0.478214

0.492649 0.458157 0.377466 0.579163 0.491924 0.513251 0.511430

0.499214 0.544519 0.538125 0.516908 0.569950 0.546345 0.941351

0.826158 0.938494 1.308552 1.081371 0.989945 0.979676 1.053178

1.047817 1.075229 1.065251 1.239957 1.109385 1.033935 0.878668

1.161400 1.159019 1.218046 1.299319 1.196866 1.166159 1.474850

1.474850

Do.BACE2 1.641839 1.559005 1.418659 1.467291 1.158249 1.285475

1.242227 1.652851 1.761645 1.653468 1.796572 2.210395 1.676976

1.743588 0.785800 0.785428 0.786314 0.785936 0.786594 0.787440

0.837922 0.796354 0.793449 0.795075 0.807961 0.667372 0.673790

0.789783 0.809125 0.853052 0.745800 0.872294 0.871700 0.829197

0.855481 0.828577 0.855827 0.823618 0.973922 0.871773 0.998582

0.079656 0.000000 0.046959 0.059493 0.098246 0.100730 0.165990

0.299236 0.266007 0.265956 0.242206 0.228887 0.338245 0.448402

0.444316 0.432776 0.386991 0.543165 0.489371 0.507521 0.529817

0.529562 0.510489 0.509886 0.502095 0.511328 0.484413 1.011635

0.917380 0.975414 1.593943 1.138626 1.055229 1.020894 1.099673

1.081069 1.120994 1.129288 1.271414 1.176670 1.005423 0.936894

1.250882 1.235977 1.268005 1.343120 1.259866 1.193274 1.526309

1.526309

Ail.me.BAC 1.565797 1.531998 1.432182 1.375840 1.155434 1.258389

1.206159 1.610502 1.716866 1.596113 1.740201 2.180519 1.657116

1.716192 0.778567 0.780646 0.779063 0.778698 0.779368 0.780196

0.828631 0.788849 0.782738 0.781906 0.799719 0.692457 0.692172

0.777079 0.824411 0.843399 0.741989 0.861913 0.861337 0.819841

0.845497 0.819290 0.855036 0.805663 0.949519 0.850563 0.984847

0.065549 0.046959 0.000000 0.071889 0.086104 0.085923 0.173117

0.289323 0.269011 0.266048 0.245202 0.213385 0.332859 0.412905

0.427373 0.399142 0.397548 0.535893 0.493601 0.507804 0.515846

0.516894 0.499704 0.496806 0.484744 0.511432 0.450766 0.989161

0.860656 0.915912 1.373269 1.112815 1.031591 1.002904 1.099091

1.076571 1.094441 1.072332 1.254181 1.155206 0.982201 0.902754

1.190043 1.213040 1.247255 1.316181 1.210096 1.152296 1.475604

1.475604

Co.BACE2 1.664728 1.549466 1.405877 1.471150 1.160015 1.319597

1.280704 1.646864 1.778072 1.642879 1.813091 2.237333 1.683609

1.763412 0.800701 0.794408 0.795299 0.794927 0.791543 0.792390

0.852143 0.813484 0.805623 0.807215 0.826186 0.707743 0.695384

0.792327 0.826094 0.862154 0.772485 0.886035 0.885431 0.844613

0.866108 0.832247 0.862566 0.820895 0.988333 0.864639 0.991412

0.082070 0.059493 0.071889 0.000000 0.095860 0.098352 0.162444

0.290263 0.256704 0.259713 0.241442 0.234326 0.323353 0.444807

0.441211 0.413959 0.393047 0.543576 0.496882 0.523419 0.524077

0.527587 0.509057 0.511805 0.484890 0.534266 0.485925 1.019390

0.918051 0.977483 1.564911 1.149407 1.048848 1.019639 1.098561

1.088542 1.128681 1.110034 1.286859 1.152039 1.011533 0.930802

1.242913 1.221040 1.289067 1.329649 1.290745 1.195257 1.513466

1.513466

Mou.BACE2 1.545932 1.573388 1.438443 1.398114 1.207899 1.319818

1.269769 1.595421 1.730609 1.695377 1.780130 2.159042 1.655239

1.729975 0.767760 0.770601 0.769083 0.768748 0.765641 0.766438

0.811591 0.775271 0.786131 0.770327 0.787598 0.683577 0.684840

0.760230 0.839664 0.834103 0.728476 0.834249 0.833713 0.810587

0.833765 0.793490 0.842144 0.780731 0.923358 0.849641 0.964120

0.114685 0.098246 0.086104 0.095860 0.000000 0.023795 0.189716

0.375615 0.273380 0.369148 0.257831 0.223910 0.343676 0.484513

0.482484 0.463142 0.388980 0.597330 0.495443 0.502171 0.491709

0.492930 0.541288 0.540553 0.540063 0.566460 0.538941 0.979940

0.832964 0.950765 1.396375 1.086735 1.031520 0.995731 1.085339

1.081155 1.097012 1.072413 1.235049 1.159829 1.037939 0.891311

1.166716 1.163106 1.243910 1.302408 1.196923 1.193162 1.484520

1.484520

Ra.BACE2 1.552856 1.567752 1.410189 1.394086 1.200769 1.306218

1.250418 1.592964 1.720713 1.685809 1.770298 2.183904 1.645395

1.738429 0.773086 0.775954 0.774421 0.774081 0.770942 0.772500

0.817749 0.780669 0.791837 0.775652 0.793096 0.691640 0.689614

0.765644 0.846854 0.839888 0.733272 0.840890 0.840348 0.817053

0.840416 0.799779 0.841506 0.786175 0.928470 0.853833 0.972134

0.112383 0.100730 0.085923 0.098352 0.023795 0.000000 0.195052

0.373516 0.274658 0.364947 0.261217 0.227012 0.354490 0.482523

0.488823 0.466689 0.375206 0.595528 0.499875 0.509181 0.490397

0.495270 0.546127 0.547707 0.538943 0.570238 0.539081 0.977436

0.829070 0.945796 1.386663 1.074541 1.024251 0.988788 1.075996

1.073573 1.089657 1.063167 1.230948 1.148653 1.033146 0.893458

1.155013 1.158194 1.239162 1.296413 1.194785 1.181860 1.463579

1.463579

Orn.an.BAC 1.657845 1.595276 1.412098 1.417040 1.110607 1.224691

1.285689 1.670587 1.745246 1.645685 1.903774 2.284561 1.734617

1.778627 0.753068 0.758362 0.753677 0.753325 0.738657 0.739485

0.805109 0.768171 0.733154 0.727709 0.764551 0.663894 0.634050

0.751510 0.771626 0.843047 0.739672 0.865196 0.864604 0.823452

0.844710 0.802981 0.811921 0.793739 0.914779 0.814783 0.949319

0.180480 0.165990 0.173117 0.162444 0.189716 0.195052 0.000000

0.329754 0.251691 0.248425 0.225336 0.229866 0.293621 0.420791

0.416220 0.390933 0.365119 0.518659 0.486749 0.514991 0.511753

0.541829 0.500113 0.502240 0.495963 0.517766 0.477199 0.958242

0.901247 0.921655 1.468968 1.113649 1.052680 1.045380 1.120001

1.104884 1.143355 1.098992 1.240637 1.106102 1.007393 0.917692

1.199729 1.161145 1.204364 1.306120 1.243665 1.171587 1.469119

1.469119

Mond2 1.575275 1.505204 1.515219 1.407925 1.273565 1.345769

1.371414 1.698887 1.687919 1.759772 1.817074 2.146297 1.673743

1.723020 0.793997 0.795330 0.793579 0.793213 0.779702 0.776906

0.824263 0.786006 0.787435 0.770897 0.802380 0.735214 0.698872

0.774122 0.845453 0.864534 0.760077 0.857617 0.857067 0.828967

0.842681 0.806573 0.863155 0.785157 0.909496 0.847642 0.939739

0.364496 0.299236 0.289323 0.290263 0.375615 0.373516 0.329754

0.000000 0.403251 0.520423 0.387425 0.361425 0.460568 0.575803

0.575153 0.544982 0.527236 0.667957 0.558414 0.592555 0.569492

0.554066 0.579755 0.590676 0.609400 0.609948 0.624342 1.030895

0.895366 0.966079 1.467906 1.110581 1.063485 1.027637 1.136052

1.131843 1.115466 1.190913 1.374302 1.206695 1.124622 0.957415

1.214740 1.223460 1.320726 1.340830 1.185651 1.181285 1.479240

1.479240

Gl.ga.BACE 1.687648 1.618952 1.473497 1.425165 1.100456 1.279564

1.308879 1.603461 1.687588 1.635090 1.760684 2.262046 1.743064

1.837810 0.779380 0.777332 0.777503 0.775726 0.774261 0.775923

0.812518 0.782286 0.764142 0.771388 0.793522 0.729808 0.707956

0.759400 0.785796 0.839143 0.768353 0.852964 0.852376 0.812355

0.832131 0.800098 0.815292 0.778559 0.983082 0.899925 1.005059

0.262664 0.266007 0.269011 0.256704 0.273380 0.274658 0.251691

0.403251 0.000000 0.064801 0.107543 0.126801 0.240994 0.391162

0.378362 0.381536 0.359797 0.465418 0.406253 0.414889 0.447249

0.471494 0.433684 0.434966 0.463906 0.465224 0.432360 1.020876

0.868312 0.979833 1.430344 1.137025 1.073325 1.061666 1.159571

1.128994 1.135282 1.112817 1.217453 1.150183 0.995054 0.909122

1.111902 1.159787 1.267104 1.361671 1.206193 1.172361 1.444613

1.444613

Tae2 1.533079 1.438069 1.357040 1.318408 1.064499 1.235155

1.256108 1.625618 1.636589 1.676114 1.708309 2.236655 1.627767

1.701040 0.765373 0.768833 0.763271 0.762871 0.760780 0.762640

0.812903 0.758920 0.741587 0.749781 0.784021 0.668554 0.642219

0.739557 0.773919 0.830422 0.744603 0.845666 0.844999 0.807992

0.821627 0.803775 0.820645 0.750682 0.933635 0.872691 0.963372

0.355555 0.265956 0.266048 0.259713 0.369148 0.364947 0.248425

0.520423 0.064801 0.000000 0.127350 0.143758 0.229687 0.488550

0.457577 0.451931 0.347961 0.566424 0.422222 0.396377 0.474403

0.480257 0.552636 0.553779 0.583924 0.607924 0.547903 0.943878

0.913468 0.855872 1.314635 1.042436 1.028235 1.022161 1.114461

1.054512 1.096675 1.025842 1.109734 1.075619 0.894968 0.910667

1.082671 1.076006 1.219406 1.283101 1.090151 1.080710 1.371884

1.371884

Pel.si.BAC 1.669804 1.589136 1.440801 1.425904 1.114790 1.223529

1.237643 1.543639 1.686258 1.550969 1.697751 2.143862 1.655321

1.751928 0.758906 0.756425 0.757068 0.754857 0.749228 0.750844

0.789194 0.759970 0.729214 0.736371 0.765802 0.695436 0.687895

0.742134 0.790398 0.800450 0.744731 0.820044 0.819481 0.789665

0.805682 0.767542 0.805252 0.755612 0.962752 0.846545 0.983225

0.252163 0.242206 0.245202 0.241442 0.257831 0.261217 0.225336

0.387425 0.107543 0.127350 0.000000 0.064717 0.223845 0.380734

0.368578 0.351596 0.357710 0.459710 0.407408 0.408177 0.450773

0.457399 0.418337 0.427066 0.438835 0.422247 0.401852 0.974191

0.803691 0.923299 1.304467 1.095357 0.980100 0.967052 1.062162

1.052444 1.057070 1.087297 1.199851 1.133027 0.956880 0.906173

1.148337 1.141336 1.238689 1.351303 1.213675 1.138261 1.427267

1.427267

Ch.pi.BACE 1.528366 1.515671 1.399690 1.315965 1.118908 1.190055

1.240458 1.543105 1.628924 1.580353 1.661555 2.039395 1.637711

1.718684 0.700365 0.699734 0.698729 0.696810 0.695613 0.696383

0.729818 0.701485 0.693941 0.691994 0.717941 0.661288 0.703211

0.685698 0.744481 0.761994 0.708613 0.762208 0.761718 0.729612

0.745441 0.719688 0.773500 0.705060 0.879899 0.800388 0.941094

0.205805 0.228887 0.213385 0.234326 0.223910 0.227012 0.229866

0.361425 0.126801 0.143758 0.064717 0.000000 0.220704 0.348298

0.347862 0.326842 0.342257 0.446154 0.393374 0.414384 0.441753

0.440076 0.405327 0.405591 0.410569 0.415587 0.400528 0.954832

0.787666 0.885039 1.181458 1.019643 0.982906 0.962612 1.074311

1.035120 1.021567 1.041497 1.142952 1.073916 0.960359 0.890985

1.080151 1.091948 1.211781 1.285834 1.148543 1.096118 1.363350

1.363350

An.cal.BAC 1.732018 1.647977 1.433326 1.342536 1.165948 1.260040

1.279716 1.577295 1.791252 1.605304 1.755088 2.274916 1.692428

1.784736 0.777382 0.774804 0.775485 0.773185 0.756661 0.757518

0.815644 0.753937 0.758366 0.763835 0.793394 0.694031 0.662189

0.766149 0.791584 0.818270 0.731348 0.831955 0.831372 0.806630

0.807730 0.783451 0.794670 0.787008 0.986177 0.842088 0.940999

0.338663 0.338245 0.332859 0.323353 0.343676 0.354490 0.293621

0.460568 0.240994 0.229687 0.223845 0.220704 0.000000 0.398461

0.392168 0.368123 0.357746 0.474531 0.460271 0.458648 0.497163

0.503334 0.489699 0.469827 0.470899 0.495138 0.444053 1.013966

0.851307 0.823973 1.282867 1.067815 1.036882 1.020109 1.096689

1.070722 1.094706 1.104692 1.141938 1.081736 1.047999 0.896546

1.141806 1.128230 1.268704 1.352538 1.199999 1.154151 1.528562

1.528562

Xo.le.BACE 1.589978 1.541594 1.489891 1.344096 1.223796 1.175702

1.319607 1.664708 1.588505 1.657126 1.640568 2.096065 1.667654

1.718918 0.845614 0.845648 0.848074 0.846387 0.841873 0.843566

0.892202 0.854111 0.864030 0.848691 0.875369 0.818034 0.826745

0.839346 0.938926 0.879404 0.806432 0.898121 0.897525 0.890940

0.878997 0.871169 0.949008 0.902804 0.969423 0.960244 0.996809

0.478214 0.448402 0.412905 0.444807 0.484513 0.482523 0.420791

0.575803 0.391162 0.488550 0.380734 0.348298 0.398461 0.000000

0.087133 0.090456 0.353412 0.602164 0.503758 0.493896 0.493016

0.497534 0.561582 0.577495 0.549039 0.593785 0.651113 1.015905

0.874716 0.999803 1.324744 1.104190 1.024779 1.053242 1.111115

1.127380 1.112550 1.085840 1.226869 1.186932 1.137892 0.877987

1.141393 1.051102 1.314854 1.284490 1.195427 1.069648 1.445308

1.445308

XolB 1.593332 1.574087 1.468726 1.351480 1.198687 1.220043

1.326215 1.666147 1.592159 1.618183 1.649721 2.079128 1.681155

1.730997 0.841803 0.841836 0.844247 0.842579 0.834053 0.835730

0.887678 0.846626 0.853632 0.839426 0.878788 0.828527 0.836645

0.832390 0.944666 0.884886 0.799764 0.889236 0.888652 0.884605

0.872817 0.860471 0.932930 0.881163 0.990606 0.951386 0.976677

0.492649 0.444316 0.427373 0.441211 0.482484 0.488823 0.416220

0.575153 0.378362 0.457577 0.368578 0.347862 0.392168 0.087133

0.000000 0.096235 0.341132 0.599575 0.526129 0.518634 0.486737

0.490064 0.561155 0.565034 0.559153 0.593166 0.633089 1.005760

0.874946 0.977651 1.351670 1.102917 1.010055 1.027291 1.093956

1.122047 1.089330 1.102954 1.206237 1.163065 1.132552 0.888858

1.128166 1.021896 1.307513 1.256954 1.189545 1.096421 1.419167

1.419167

Xo.tr.BACE 1.613040 1.582216 1.476458 1.357746 1.242398 1.202965

1.329205 1.638304 1.594669 1.653044 1.680689 2.080535 1.658193

1.702337 0.865411 0.865089 0.867899 0.866149 0.853377 0.852044

0.912917 0.867462 0.870327 0.860135 0.904720 0.837012 0.835360

0.851960 0.941352 0.915822 0.818829 0.920149 0.918842 0.902674

0.890568 0.879858 0.937954 0.905722 1.012947 0.967918 0.972183

0.458157 0.432776 0.399142 0.413959 0.463142 0.466689 0.390933

0.544982 0.381536 0.451931 0.351596 0.326842 0.368123 0.090456

0.096235 0.000000 0.332490 0.594186 0.526166 0.547418 0.484395

0.499332 0.556142 0.568898 0.549937 0.604603 0.630504 1.018350

0.845654 0.965393 1.324953 1.108759 1.011458 1.038734 1.107210

1.125850 1.083956 1.060199 1.207261 1.136035 1.128920 0.890669

1.138997 1.045062 1.306427 1.290786 1.209740 1.076445 1.422035

1.422035

Am.ti.BACE 1.838675 1.815530 1.460497 1.445851 1.303566 1.377922

1.288158 1.727274 1.763606 1.645568 1.835564 1.873492 1.553255

1.622929 1.013638 1.006071 1.008615 1.008482 1.012303 1.012303

1.161863 1.026017 1.042600 1.032633 1.061163 0.892738 0.991439

1.029133 1.052710 1.032145 0.891740 1.055818 1.055818 1.016262

1.042194 1.015003 1.010749 1.029121 0.972113 1.130175 1.105279

0.377466 0.386991 0.397548 0.393047 0.388980 0.375206 0.365119

0.527236 0.359797 0.347961 0.357710 0.342257 0.357746 0.353412

0.341132 0.332490 0.000000 0.552457 0.421473 0.323970 0.499748

0.497148 0.491562 0.508942 0.446229 0.475062 0.581226 1.185869

0.759669 1.049762 2.416162 1.347660 1.238078 1.230818 1.207412

1.274826 1.224401 1.441000 1.447708 1.217007 1.120914 0.377863

1.492527 1.321744 1.234964 1.479913 1.344644 1.421884 1.534692

1.534692

Da.re.BACE 1.602837 1.669884 1.429652 1.362494 1.236866 1.209554

1.281756 1.637889 1.695790 1.648237 1.747098 2.200490 1.652800

1.749646 0.716318 0.716676 0.715734 0.715440 0.706404 0.705400

0.740328 0.701610 0.700123 0.700644 0.704207 0.714222 0.709786

0.712359 0.776229 0.744503 0.658334 0.733622 0.738375 0.724445

0.730195 0.717463 0.792780 0.707130 0.811991 0.793147 0.863277

0.579163 0.543165 0.535893 0.543576 0.597330 0.595528 0.518659

0.667957 0.465418 0.566424 0.459710 0.446154 0.474531 0.602164

0.599575 0.594186 0.552457 0.000000 0.238122 0.227172 0.280480

0.279281 0.351410 0.299776 0.309697 0.340862 0.559796 0.930124

0.813739 0.959849 1.368093 0.993326 0.996635 0.996489 1.097928

1.097213 1.107736 0.987641 1.163899 1.041253 1.089559 0.871481

1.129757 1.061857 1.200464 1.259879 1.157356 1.094581 1.375549

1.375549

On.my.BACE 1.683221 1.642846 1.488126 1.379254 1.168919 1.276591

1.271535 1.660652 1.676658 1.580027 1.742755 2.181090 1.635259

1.681107 0.734931 0.735647 0.734892 0.732675 0.718382 0.722973

0.767398 0.720607 0.708940 0.712506 0.730114 0.723314 0.710784

0.742688 0.738541 0.711019 0.661794 0.704659 0.709753 0.706256

0.709474 0.694653 0.758824 0.736496 0.868958 0.774917 0.865334

0.491924 0.489371 0.493601 0.496882 0.495443 0.499875 0.486749

0.558414 0.406253 0.422222 0.407408 0.393374 0.460271 0.503758

0.526129 0.526166 0.421473 0.238122 0.000000 0.045264 0.265357

0.273778 0.230007 0.219052 0.256587 0.263922 0.489244 0.925474

0.791649 0.924671 1.382524 1.000424 1.050808 1.002343 1.090984

1.052231 1.088773 1.017517 1.114555 1.044476 0.921529 0.852689

1.152384 1.089164 1.262708 1.280794 1.197487 1.086180 1.431889

1.431889

Sal.sala.B 1.993668 1.879921 1.599311 1.632696 1.413023 1.467369

1.408080 1.787569 1.741554 1.668232 1.837081 2.604304 1.883725

1.909160 0.756209 0.761147 0.756209 0.756209 0.735228 0.742842

0.816009 0.736435 0.703617 0.709936 0.727797 0.814748 0.715754

0.764832 0.704776 0.715524 0.701753 0.691354 0.691354 0.708548

0.714058 0.698739 0.704229 0.727969 0.860852 0.847789 0.926819

0.513251 0.507521 0.507804 0.523419 0.502171 0.509181 0.514991

0.592555 0.414889 0.396377 0.408177 0.414384 0.458648 0.493896

0.518634 0.547418 0.323970 0.227172 0.045264 0.000000 0.257459

0.246882 0.189256 0.183447 0.237185 0.241030 0.497666 0.885823

0.756605 1.065673 1.459025 1.033861 1.110400 1.029764 1.065651

1.139621 1.108759 0.970424 1.027178 0.958101 0.955690 0.943619

1.158225 1.097533 1.298374 1.325655 1.073764 1.160902 1.589421

1.589421

Tak.ru.BAC 1.581537 1.589072 1.404069 1.304017 1.192865 1.265015

1.211602 1.626017 1.610342 1.574258 1.755818 2.148171 1.708496

1.744668 0.734862 0.739504 0.734826 0.732746 0.707034 0.711332

0.767338 0.730581 0.729114 0.715361 0.764615 0.783191 0.739400

0.715582 0.818703 0.733471 0.653694 0.728458 0.727982 0.719063

0.728281 0.709575 0.795649 0.750837 0.853765 0.784020 0.881506

0.511430 0.529817 0.515846 0.524077 0.491709 0.490397 0.511753

0.569492 0.447249 0.474403 0.450773 0.441753 0.497163 0.493016

0.486737 0.484395 0.499748 0.280480 0.265357 0.257459 0.000000

0.139201 0.173310 0.184842 0.258789 0.240427 0.496288 0.874146

0.747715 0.921410 1.378863 1.000472 0.965236 0.984292 1.066873

1.026231 1.041293 1.057790 1.131778 1.101321 0.953602 0.801075

1.138689 1.030480 1.190552 1.286079 1.149318 1.040783 1.366549

1.366549

Te.ng.BACE 1.637437 1.626157 1.383224 1.368154 1.211134 1.208969

1.174972 1.661665 1.700076 1.630772 1.754933 2.213390 1.694271

1.768197 0.759773 0.754372 0.759746 0.757595 0.736040 0.732912

0.777954 0.736352 0.760886 0.732578 0.772385 0.809537 0.798327

0.730778 0.841960 0.744925 0.658772 0.736715 0.736228 0.731794

0.731083 0.739896 0.846608 0.743016 0.823240 0.804093 0.860928

0.499214 0.529562 0.516894 0.527587 0.492930 0.495270 0.541829

0.554066 0.471494 0.480257 0.457399 0.440076 0.503334 0.497534

0.490064 0.499332 0.497148 0.279281 0.273778 0.246882 0.139201

0.000000 0.189197 0.201315 0.259755 0.241164 0.505583 0.922274

0.775403 0.931910 1.310714 0.985420 0.950934 0.940171 1.021508

1.033110 1.027720 1.044796 1.204575 1.080092 1.043612 0.854594

1.169168 1.064793 1.261166 1.263423 1.161920 1.085087 1.370479

1.370479

Gs2 1.628853 1.659017 1.520188 1.372170 1.243600 1.271458

1.240186 1.593823 1.591453 1.609512 1.704784 2.180346 1.681248

1.718503 0.726342 0.724990 0.726302 0.724250 0.703022 0.699586

0.755523 0.717387 0.705979 0.706114 0.743567 0.779629 0.750624

0.709746 0.813292 0.733977 0.662302 0.732941 0.732461 0.724465

0.730052 0.721115 0.802860 0.728995 0.834210 0.778745 0.857807

0.544519 0.510489 0.499704 0.509057 0.541288 0.546127 0.500113

0.579755 0.433684 0.552636 0.418337 0.405327 0.489699 0.561582

0.561155 0.556142 0.491562 0.351410 0.230007 0.189256 0.173310

0.189197 0.000000 0.185003 0.261588 0.250680 0.549115 0.944817

0.793819 0.965999 1.288102 1.004624 1.014219 0.979078 1.079324

1.049379 1.036246 1.024786 1.201271 1.082270 0.967516 0.875779

1.109799 1.075952 1.266747 1.232390 1.139890 1.074919 1.386987

1.386987

Ore.nl.BAC 1.578803 1.602337 1.426461 1.293920 1.224042 1.233152

1.247200 1.647189 1.673878 1.631242 1.715994 2.082841 1.639799

1.682391 0.703618 0.702628 0.703570 0.701596 0.676594 0.673289

0.726534 0.695687 0.688196 0.682309 0.710222 0.742885 0.713907

0.651903 0.776717 0.676858 0.627133 0.685008 0.689683 0.673627

0.682758 0.673529 0.759041 0.698567 0.817941 0.766931 0.838113

0.538125 0.509886 0.496806 0.511805 0.540553 0.547707 0.502240

0.590676 0.434966 0.553779 0.427066 0.405591 0.469827 0.577495

0.565034 0.568898 0.508942 0.299776 0.219052 0.183447 0.184842

0.201315 0.185003 0.000000 0.246053 0.206386 0.549671 0.923768

0.758022 0.939599 1.279256 1.015379 1.034380 1.004084 1.094036

1.080499 1.083746 0.974881 1.170830 1.130058 0.999228 0.872923

1.127742 1.057095 1.249981 1.267917 1.153700 1.057755 1.388271

1.388271

Gd.mo.BACE 1.645475 1.669200 1.416617 1.354793 1.218158 1.265477

1.276907 1.586842 1.586661 1.592745 1.606928 2.050414 1.610257

1.606497 0.727928 0.727607 0.727890 0.726543 0.711209 0.707781

0.756201 0.712288 0.708695 0.700062 0.723637 0.724001 0.714921

0.690383 0.774238 0.692851 0.630101 0.699827 0.699369 0.694279

0.685654 0.687527 0.756861 0.720715 0.825386 0.768475 0.870858

0.516908 0.502095 0.484744 0.484890 0.540063 0.538943 0.495963

0.609400 0.463906 0.583924 0.438835 0.410569 0.470899 0.549039

0.559153 0.549937 0.446229 0.309697 0.256587 0.237185 0.258789

0.259755 0.261588 0.246053 0.000000 0.271401 0.574112 0.893363

0.788593 0.907020 1.226326 1.005315 1.029893 0.998916 1.075772

1.068403 1.061067 1.001589 1.157849 1.095314 0.968377 0.903685

1.101987 1.061973 1.197494 1.259694 1.180353 1.058906 1.408037

1.408037

Ory.lt.BAC 1.638852 1.607946 1.395233 1.385947 1.185803 1.264193

1.232339 1.633786 1.660704 1.678955 1.696146 2.131531 1.659853

1.711966 0.654277 0.659739 0.654277 0.654277 0.643081 0.639799

0.679397 0.651767 0.641200 0.633337 0.675874 0.693673 0.700032

0.630968 0.674947 0.630152 0.623732 0.631374 0.630935 0.633243

0.639622 0.624829 0.691430 0.665607 0.828545 0.717394 0.817729

0.569950 0.511328 0.511432 0.534266 0.566460 0.570238 0.517766

0.609948 0.465224 0.607924 0.422247 0.415587 0.495138 0.593785

0.593166 0.604603 0.475062 0.340862 0.263922 0.241030 0.240427

0.241164 0.250680 0.206386 0.271401 0.000000 0.556025 0.880504

0.797081 0.898776 1.268614 0.922533 0.952701 0.930529 0.999471

0.987677 0.991291 0.932944 1.033182 0.993459 0.908125 0.912405

1.034045 1.044226 1.256201 1.232343 1.085205 1.072173 1.381393

1.381393

La.chu.BAC 1.671997 1.725090 1.556075 1.269110 1.238466 1.263386

1.318092 1.563905 1.650518 1.752318 1.750957 2.161561 1.579199

1.698643 0.652961 0.653049 0.652961 0.652961 0.644809 0.637472

0.660435 0.644006 0.624783 0.633581 0.673810 0.663095 0.635236

0.650138 0.666740 0.693341 0.651038 0.689577 0.689002 0.668014

0.688174 0.676449 0.725880 0.661979 0.996493 0.746659 0.890150

0.546345 0.484413 0.450766 0.485925 0.538941 0.539081 0.477199

0.624342 0.432360 0.547903 0.401852 0.400528 0.444053 0.651113

0.633089 0.630504 0.581226 0.559796 0.489244 0.497666 0.496288

0.505583 0.549115 0.549671 0.574112 0.556025 0.000000 0.976774

0.852813 0.878768 1.168146 0.916054 1.014731 0.988854 1.050738

1.074966 1.035067 1.025586 1.095607 1.173179 1.163708 0.875190

1.109196 1.001614 1.215967 1.215927 1.259018 1.076670 1.536887

1.536887

Stn.purp 1.362062 1.267995 1.170904 1.383329 1.258066 1.311442

1.257665 1.700440 1.647190 1.765052 1.744823 2.114041 1.717041

1.761424 0.864379 0.864401 0.862186 0.861780 0.848469 0.845427

0.908373 0.834875 0.848542 0.838658 0.863534 0.808667 0.775009

0.822904 0.967701 0.824680 0.690106 0.828217 0.822806 0.808957

0.837249 0.811833 0.888142 0.843956 0.869594 0.869768 0.842190

0.941351 1.011635 0.989161 1.019390 0.979940 0.977436 0.958242

1.030895 1.020876 0.943878 0.974191 0.954832 1.013966 1.015905

1.005760 1.018350 1.185869 0.930124 0.925474 0.885823 0.874146

0.922274 0.944817 0.923768 0.893363 0.880504 0.976774 0.000000

0.213639 0.893089 1.092224 0.972550 0.937160 0.981300 0.979265

1.003242 1.087071 0.968095 1.010886 0.982564 0.925766 0.864274

0.953081 1.080687 1.233694 1.221938 1.144210 1.134439 1.477040

1.477040

Pa.livi 1.549205 1.353647 1.170967 1.386471 1.373851 1.320379

1.347914 1.763883 1.779606 1.763414 1.873697 2.547891 1.929020

1.938440 0.660573 0.650528 0.660573 0.660573 0.634239 0.631870

0.678418 0.620184 0.641270 0.616057 0.650553 0.824590 0.694432

0.616131 0.720600 0.644116 0.632459 0.614483 0.611880 0.609247

0.622226 0.607531 0.703086 0.640257 0.832060 0.704051 0.673874

0.826158 0.917380 0.860656 0.918051 0.832964 0.829070 0.901247

0.895366 0.868312 0.913468 0.803691 0.787666 0.851307 0.874716

0.874946 0.845654 0.759669 0.813739 0.791649 0.756605 0.747715

0.775403 0.793819 0.758022 0.788593 0.797081 0.852813 0.213639

0.000000 0.957451 0.998433 0.932089 0.882933 0.879526 0.912738

0.906444 1.070479 0.906447 0.689078 0.732243 0.702134 0.959568

0.853354 1.087392 1.239627 1.153151 1.129976 1.084427 1.540622

1.540622

Me.mere 1.385979 1.244088 1.190832 1.343478 1.228934 1.353964

1.435917 1.821251 1.882471 1.894535 1.818540 2.148348 1.940041

1.908567 0.800388 0.811198 0.800388 0.800388 0.806287 0.797327

0.846401 0.818686 0.797733 0.780196 0.806812 0.720843 0.753530

0.779726 0.762100 0.754143 0.757213 0.745666 0.745002 0.737337

0.736909 0.748379 0.745412 0.803108 0.938593 0.809563 0.785427

0.938494 0.975414 0.915912 0.977483 0.950765 0.945796 0.921655

0.966079 0.979833 0.855872 0.923299 0.885039 0.823973 0.999803

0.977651 0.965393 1.049762 0.959849 0.924671 1.065673 0.921410

0.931910 0.965999 0.939599 0.907020 0.898776 0.878768 0.893089

0.957451 0.000000 0.616756 0.851199 1.007953 1.007988 0.906360

0.902019 1.010143 1.025165 0.617854 0.733509 0.800180 0.785709

0.900137 1.035399 1.232543 1.316034 1.179733 1.159708 1.663777

1.663777

Ru.phil 1.643834 1.905711 2.267019 1.567862 1.511309 1.496852

1.637506 1.631547 1.921877 1.888651 1.792482 2.228688 1.775148

1.702450 1.235625 1.243413 1.235625 1.235625 1.235625 1.214875

1.246729 1.303395 1.284583 1.217152 1.209208 2.277533 2.495439

1.164368 2.354950 1.216120 1.214675 1.242272 1.240298 1.250081

1.207095 1.262789 1.839839 1.223082 1.188069 1.318957 1.296552

1.308552 1.593943 1.373269 1.564911 1.396375 1.386663 1.468968

1.467906 1.430344 1.314635 1.304467 1.181458 1.282867 1.324744

1.351670 1.324953 2.416162 1.368093 1.382524 1.459025 1.378863

1.310714 1.288102 1.279256 1.226326 1.268614 1.168146 1.092224

0.998433 0.616756 0.000000 0.956446 1.119003 1.185030 1.394994

1.242547 1.137549 1.141751 1.982128 1.945084 2.151500 1.111465

1.171594 1.254742 1.658255 1.583980 1.526552 1.382151 1.961767

1.961767

Cr.giga 1.430833 1.494717 1.347475 1.390667 1.305590 1.320599

1.403216 1.743919 1.900050 1.914183 2.034847 2.289704 1.919427

1.940611 0.857405 0.865827 0.857906 0.856277 0.857657 0.851159

0.895440 0.847122 0.847419 0.824016 0.836507 0.877075 0.965571

0.863388 1.021705 0.898949 0.788740 0.868401 0.867859 0.894336

0.901569 0.890495 0.968676 0.852533 0.851059 0.934907 0.795218

1.081371 1.138626 1.112815 1.149407 1.086735 1.074541 1.113649

1.110581 1.137025 1.042436 1.095357 1.019643 1.067815 1.104190

1.102917 1.108759 1.347660 0.993326 1.000424 1.033861 1.000472

0.985420 1.004624 1.015379 1.005315 0.922533 0.916054 0.972550

0.932089 0.851199 0.956446 0.000000 1.016399 1.000127 1.022232

0.956057 1.034799 0.958763 0.866383 0.993691 0.945394 0.737808

0.931858 0.977671 1.295474 1.340733 1.221052 1.145030 1.465546

1.465546

N.vect 1.587157 1.492499 1.363472 1.448968 1.261056 1.275626

1.293781 1.727516 1.969919 1.771864 1.967418 2.445910 1.844547

1.836027 0.830244 0.821828 0.829855 0.829855 0.841774 0.838276

0.840092 0.815295 0.847079 0.828291 0.836609 0.865669 1.049441

0.820108 0.993813 0.821252 0.766481 0.818057 0.823149 0.832409

0.809643 0.818944 0.962575 0.842726 0.922676 0.855184 0.734414

0.989945 1.055229 1.031591 1.048848 1.031520 1.024251 1.052680

1.063485 1.073325 1.028235 0.980100 0.982906 1.036882 1.024779

1.010055 1.011458 1.238078 0.996635 1.050808 1.110400 0.965236

0.950934 1.014219 1.034380 1.029893 0.952701 1.014731 0.937160

0.882933 1.007953 1.119003 1.016399 0.000000 0.178333 0.161416

0.241371 0.307004 0.877135 0.889235 0.930104 0.821206 0.850124

0.983802 1.115222 1.224244 1.214893 1.250829 0.994572 1.485730

1.485730

Aip.pall 1.629472 1.463865 1.369731 1.465599 1.295415 1.390582

1.369589 1.782040 1.998080 1.803676 2.008548 2.577355 1.856447

1.864450 0.841640 0.835974 0.843909 0.843909 0.855578 0.852107

0.856908 0.831521 0.854856 0.838300 0.849293 0.864298 0.984835

0.842545 0.999516 0.840766 0.752529 0.831788 0.831615 0.844342

0.828653 0.833213 0.955857 0.817910 0.941218 0.818442 0.731647

0.979676 1.020894 1.002904 1.019639 0.995731 0.988788 1.045380

1.027637 1.061666 1.022161 0.967052 0.962612 1.020109 1.053242

1.027291 1.038734 1.230818 0.996489 1.002343 1.029764 0.984292

0.940171 0.979078 1.004084 0.998916 0.930529 0.988854 0.981300

0.879526 1.007988 1.185030 1.000127 0.178333 0.000000 0.121979

0.233498 0.300031 0.859860 0.892293 0.956222 0.805815 0.907515

1.005931 1.156095 1.246366 1.257087 1.286748 1.001651 1.459197

1.459197

Ac.mill 1.886854 1.615868 1.384795 1.590934 1.386608 1.458682

1.391965 1.850140 2.039728 1.820351 2.091880 2.711795 1.807197

1.835521 0.891524 0.888358 0.891552 0.891552 0.905348 0.901053

0.918481 0.864746 0.886984 0.883557 0.898356 0.831099 0.978763

0.868881 0.987294 0.876066 0.797769 0.874448 0.874448 0.867545

0.854677 0.866224 0.929039 0.873208 0.950301 0.882574 0.738212

1.053178 1.099673 1.099091 1.098561 1.085339 1.075996 1.120001

1.136052 1.159571 1.114461 1.062162 1.074311 1.096689 1.111115

1.093956 1.107210 1.207412 1.097928 1.090984 1.065651 1.066873

1.021508 1.079324 1.094036 1.075772 0.999471 1.050738 0.979265

0.912738 0.906360 1.394994 1.022232 0.161416 0.121979 0.000000

0.227175 0.232972 0.825541 0.915651 0.947702 0.824754 0.904672

0.973115 1.157463 1.325657 1.268050 1.387937 1.050124 1.510913

1.510913

Hy.mag 1.666573 1.487520 1.389978 1.377137 1.250880 1.281741

1.281247 1.714951 1.927043 1.845866 1.987710 2.545071 1.776147

1.862428 0.877743 0.871580 0.872572 0.869992 0.875784 0.874387

0.885170 0.860799 0.878789 0.875448 0.880423 0.919993 1.074047

0.871740 1.055080 0.888645 0.836681 0.892276 0.891663 0.900747

0.889452 0.884694 0.977926 0.827190 0.953109 0.831121 0.723209

1.047817 1.081069 1.076571 1.088542 1.081155 1.073573 1.104884

1.131843 1.128994 1.054512 1.052444 1.035120 1.070722 1.127380

1.122047 1.125850 1.274826 1.097213 1.052231 1.139621 1.026231

1.033110 1.049379 1.080499 1.068403 0.987677 1.074966 1.003242

0.906444 0.902019 1.242547 0.956057 0.241371 0.233498 0.227175

0.000000 0.201612 0.862206 0.977907 1.038583 0.892210 0.836588

1.001893 1.138307 1.179063 1.212591 1.249894 0.967962 1.444826

1.444826

Cly.hem 1.540809 1.446022 1.343951 1.443902 1.317640 1.324165

1.275001 1.670158 1.926207 1.802540 1.917276 2.381806 1.841210

1.868235 0.913450 0.908752 0.913972 0.911397 0.914159 0.910421

0.951864 0.908690 0.927601 0.910357 0.925143 0.890175 1.042270

0.894399 1.027895 0.909058 0.876735 0.923502 0.922904 0.935201

0.917399 0.926122 0.984439 0.858773 1.037130 0.860542 0.795559

1.075229 1.120994 1.094441 1.128681 1.097012 1.089657 1.143355

1.115466 1.135282 1.096675 1.057070 1.021567 1.094706 1.112550

1.089330 1.083956 1.224401 1.107736 1.088773 1.108759 1.041293

1.027720 1.036246 1.083746 1.061067 0.991291 1.035067 1.087071

1.070479 1.010143 1.137549 1.034799 0.307004 0.300031 0.232972

0.201612 0.000000 0.901015 0.935047 0.975851 0.887611 0.919366

1.053756 1.188425 1.297401 1.290231 1.285559 1.080463 1.552370

1.552370

Ca.tela 1.454586 1.327809 1.172273 1.442767 1.284177 1.279877

1.364424 1.768409 1.786073 1.697787 1.857556 2.238450 1.815940

1.796884 0.861988 0.863854 0.856695 0.856695 0.866432 0.865132

0.909014 0.893894 0.849070 0.856439 0.867802 0.861331 0.940370

0.872290 0.965090 0.890892 0.851007 0.911464 0.911979 0.896959

0.906467 0.883498 0.891677 0.840094 0.962881 0.952625 0.694809

1.065251 1.129288 1.072332 1.110034 1.072413 1.063167 1.098992

1.190913 1.112817 1.025842 1.087297 1.041497 1.104692 1.085840

1.102954 1.060199 1.441000 0.987641 1.017517 0.970424 1.057790

1.044796 1.024786 0.974881 1.001589 0.932944 1.025586 0.968095

0.906447 1.025165 1.141751 0.958763 0.877135 0.859860 0.825541

0.862206 0.901015 0.000000 0.871846 0.897502 0.860394 0.832817

0.966585 1.075361 1.296532 1.265396 1.158110 1.128093 1.572988

1.572988

V.lien 1.329414 1.157809 1.152824 1.458773 1.118986 1.363411

1.375660 1.760257 1.818214 1.768015 2.053069 2.088164 2.045762

1.944322 0.919016 0.920179 0.921542 0.918328 0.923279 0.923279

0.976971 0.953586 0.911238 0.909456 0.915189 0.826529 0.845927

0.942147 0.924023 0.918647 0.785370 0.914079 0.914079 0.943891

0.940691 0.937302 0.934541 0.974762 0.998515 1.046925 0.857035

1.239957 1.271414 1.254181 1.286859 1.235049 1.230948 1.240637

1.374302 1.217453 1.109734 1.199851 1.142952 1.141938 1.226869

1.206237 1.207261 1.447708 1.163899 1.114555 1.027178 1.131778

1.204575 1.201271 1.170830 1.157849 1.033182 1.095607 1.010886

0.689078 0.617854 1.982128 0.866383 0.889235 0.892293 0.915651

0.977907 0.935047 0.871846 0.000000 0.822616 0.753086 0.660668

0.917797 0.982888 1.314125 1.318003 1.185514 0.989467 1.409395

1.409395

E.scolo 1.336382 1.140828 1.218049 1.429669 1.279919 1.284069

1.349873 1.682576 1.774506 1.862480 1.935135 1.966682 1.937597

1.818929 0.905956 0.909582 0.911591 0.911613 0.915834 0.915834

1.000223 0.890003 0.889988 0.891275 0.882803 0.769380 0.852917

0.927240 0.892477 0.918933 0.864668 0.912278 0.912278 0.922802

0.924383 0.924563 0.914416 0.889912 0.885917 1.060694 0.793969

1.109385 1.176670 1.155206 1.152039 1.159829 1.148653 1.106102

1.206695 1.150183 1.075619 1.133027 1.073916 1.081736 1.186932

1.163065 1.136035 1.217007 1.041253 1.044476 0.958101 1.101321

1.080092 1.082270 1.130058 1.095314 0.993459 1.173179 0.982564

0.732243 0.733509 1.945084 0.993691 0.930104 0.956222 0.947702

1.038583 0.975851 0.897502 0.822616 0.000000 0.778321 0.602174

0.929640 0.938149 1.314789 1.370037 1.265759 1.116298 1.355619

1.355619

Sac.kowa 1.453909 1.218909 1.216350 1.448341 1.140927 1.379295

1.296607 1.916012 1.620712 1.773494 1.958087 2.059615 1.902647

1.868874 0.747777 0.760621 0.751162 0.751162 0.758096 0.758096

0.802117 0.769289 0.756684 0.758606 0.755623 0.713682 0.794432

0.763323 0.767862 0.775233 0.723101 0.779786 0.779786 0.771546

0.770982 0.760838 0.755972 0.770304 0.840912 0.841605 0.619124

1.033935 1.005423 0.982201 1.011533 1.037939 1.033146 1.007393

1.124622 0.995054 0.894968 0.956880 0.960359 1.047999 1.137892

1.132552 1.128920 1.120914 1.089559 0.921529 0.955690 0.953602

1.043612 0.967516 0.999228 0.968377 0.908125 1.163708 0.925766

0.702134 0.800180 2.151500 0.945394 0.821206 0.805815 0.824754

0.892210 0.887611 0.860394 0.753086 0.778321 0.000000 0.518241

0.834385 1.065154 1.187863 1.300595 1.230509 0.951187 1.320222

1.320222

I.obso 1.446769 1.346488 1.254215 1.219900 1.222259 1.122629

1.223670 1.752510 1.821756 1.877079 1.935348 2.345588 1.834556

1.805278 0.787292 0.802896 0.787292 0.787292 0.787374 0.781212

0.857566 0.757587 0.718418 0.755010 0.777008 0.775299 0.782055

0.773147 0.872237 0.727549 0.771477 0.776294 0.775468 0.772303

0.770588 0.734981 0.740165 0.771969 0.968361 0.893042 0.759807

0.878668 0.936894 0.902754 0.930802 0.891311 0.893458 0.917692

0.957415 0.909122 0.910667 0.906173 0.890985 0.896546 0.877987

0.888858 0.890669 0.377863 0.871481 0.852689 0.943619 0.801075

0.854594 0.875779 0.872923 0.903685 0.912405 0.875190 0.864274

0.959568 0.785709 1.111465 0.737808 0.850124 0.907515 0.904672

0.836588 0.919366 0.832817 0.660668 0.602174 0.518241 0.000000

0.769687 0.744725 1.135210 1.162097 1.138400 1.068372 1.529387

1.529387

Lo.giga 1.456271 1.311145 1.280926 1.438448 1.376643 1.407408

1.471632 1.743396 1.720346 1.694608 1.838496 2.201145 1.840917

1.766773 0.919971 0.921500 0.917704 0.915798 0.917874 0.919626

0.932962 0.914313 0.894584 0.891685 0.891483 0.947183 0.968831

0.901452 0.982398 0.936952 0.911853 0.944919 0.944320 0.941102

0.941553 0.927380 0.954089 0.889233 0.922368 1.028064 0.822916

1.161400 1.250882 1.190043 1.242913 1.166716 1.155013 1.199729

1.214740 1.111902 1.082671 1.148337 1.080151 1.141806 1.141393

1.128166 1.138997 1.492527 1.129757 1.152384 1.158225 1.138689

1.169168 1.109799 1.127742 1.101987 1.034045 1.109196 0.953081

0.853354 0.900137 1.171594 0.931858 0.983802 1.005931 0.973115

1.001893 1.053756 0.966585 0.917797 0.929640 0.834385 0.769687

0.000000 1.003011 1.325633 1.357303 1.238866 1.164859 1.375265

1.375265

Ly.stagn 1.574071 1.379494 1.232505 1.400030 1.230499 1.461408

1.358513 1.635906 1.759020 1.871451 1.847921 2.209419 1.772181

1.907500 0.983301 0.982612 0.984831 0.984816 0.972086 0.973931

1.040551 1.001070 0.983358 0.977070 0.978569 0.870587 0.971030

0.973071 1.052412 0.949586 0.887745 0.991268 0.987991 0.987173

0.996289 0.987699 1.032741 1.005841 0.973100 1.165886 1.080327

1.159019 1.235977 1.213040 1.221040 1.163106 1.158194 1.161145

1.223460 1.159787 1.076006 1.141336 1.091948 1.128230 1.051102

1.021896 1.045062 1.321744 1.061857 1.089164 1.097533 1.030480

1.064793 1.075952 1.057095 1.061973 1.044226 1.001614 1.080687

1.087392 1.035399 1.254742 0.977671 1.115222 1.156095 1.157463

1.138307 1.188425 1.075361 0.982888 0.938149 1.065154 0.744725

1.003011 0.000000 1.176824 1.290139 1.171056 1.211437 1.570786

1.570786

Ci.inte 1.758595 1.613297 1.514081 1.428297 1.402399 1.362687

1.461246 1.806717 1.777293 1.802603 1.891125 2.320104 1.787279

1.979341 1.182554 1.181058 1.177514 1.177514 1.176561 1.170791

1.253308 1.186898 1.180005 1.172772 1.175605 1.075586 1.265517

1.184944 1.370495 1.230980 1.140508 1.215337 1.214628 1.223603

1.221782 1.208993 1.264416 1.150940 1.224723 1.227508 1.160648

1.218046 1.268005 1.247255 1.289067 1.243910 1.239162 1.204364

1.320726 1.267104 1.219406 1.238689 1.211781 1.268704 1.314854

1.307513 1.306427 1.234964 1.200464 1.262708 1.298374 1.190552

1.261166 1.266747 1.249981 1.197494 1.256201 1.215967 1.233694

1.239627 1.232543 1.658255 1.295474 1.224244 1.246366 1.325657

1.179063 1.297401 1.296532 1.314125 1.314789 1.187863 1.135210

1.325633 1.176824 0.000000 0.570026 0.936307 1.230634 1.410909

1.410909

Ci.savi 1.749677 1.649315 1.589418 1.465514 1.544230 1.402366

1.460015 1.996165 1.931694 1.729734 2.073870 2.504390 1.873965

1.966915 1.060704 1.055884 1.055749 1.055749 1.067017 1.058574

1.105402 1.056676 1.098261 1.086580 1.074323 1.043888 1.264096

1.127299 1.293571 1.126227 1.016309 1.078213 1.077456 1.091379

1.087831 1.105936 1.206696 1.078107 1.214778 1.211726 1.139600

1.299319 1.343120 1.316181 1.329649 1.302408 1.296413 1.306120

1.340830 1.361671 1.283101 1.351303 1.285834 1.352538 1.284490

1.256954 1.290786 1.479913 1.259879 1.280794 1.325655 1.286079

1.263423 1.232390 1.267917 1.259694 1.232343 1.215927 1.221938

1.153151 1.316034 1.583980 1.340733 1.214893 1.257087 1.268050

1.212591 1.290231 1.265396 1.318003 1.370037 1.300595 1.162097

1.357303 1.290139 0.570026 0.000000 1.071352 1.245286 1.535036

1.535036

Ha.rore 1.601640 1.534614 1.338761 1.519590 1.379022 1.376140

1.429797 1.945225 1.744977 1.972724 1.919048 2.350305 2.012422

2.089627 1.126508 1.124240 1.121388 1.121388 1.136336 1.137316

1.213687 1.130640 1.110168 1.121152 1.094048 1.206328 1.291022

1.138111 1.340801 1.179762 1.094942 1.165725 1.160714 1.169707

1.149961 1.142527 1.209930 1.117138 1.168892 1.189248 1.121601

1.196866 1.259866 1.210096 1.290745 1.196923 1.194785 1.243665

1.185651 1.206193 1.090151 1.213675 1.148543 1.199999 1.195427

1.189545 1.209740 1.344644 1.157356 1.197487 1.073764 1.149318

1.161920 1.139890 1.153700 1.180353 1.085205 1.259018 1.144210

1.129976 1.179733 1.526552 1.221052 1.250829 1.286748 1.387937

1.249894 1.285559 1.158110 1.185514 1.265759 1.230509 1.138400

1.238866 1.171056 0.936307 1.071352 0.000000 1.264985 1.540678

1.540678

TrA 1.528601 1.487652 1.416550 1.377130 1.031994 1.348810

1.297496 1.549109 1.651417 1.806161 1.827079 2.329973 1.824381

1.847608 0.935221 0.942676 0.935221 0.935221 0.924959 0.919867

0.968493 0.966970 0.977192 0.978723 0.963168 1.032253 1.172536

0.989571 1.131110 0.980011 1.000250 0.959391 0.958649 0.955825

0.950094 0.936000 0.993131 0.964350 1.103922 1.000163 1.040486

1.166159 1.193274 1.152296 1.195257 1.193162 1.181860 1.171587

1.181285 1.172361 1.080710 1.138261 1.096118 1.154151 1.069648

1.096421 1.076445 1.421884 1.094581 1.086180 1.160902 1.040783

1.085087 1.074919 1.057755 1.058906 1.072173 1.076670 1.134439

1.084427 1.159708 1.382151 1.145030 0.994572 1.001651 1.050124

0.967962 1.080463 1.128093 0.989467 1.116298 0.951187 1.068372

1.164859 1.211437 1.230634 1.245286 1.264985 0.000000 1.016811

1.016811

Tr.ad.cath 1.754986 1.728221 1.640797 1.629985 1.269503 1.574753

1.546710 1.794823 1.738978 1.671900 1.990165 2.406692 1.990751

1.956686 1.423375 1.428856 1.423375 1.423375 1.422968 1.423972

1.416023 1.438032 1.393100 1.398805 1.397962 1.450973 1.691218

1.428428 1.507761 1.489034 1.529039 1.480710 1.479522 1.475101

1.464839 1.448704 1.445306 1.354668 1.450811 1.421487 1.401252

1.474850 1.526309 1.475604 1.513466 1.484520 1.463579 1.469119

1.479240 1.444613 1.371884 1.427267 1.363350 1.528562 1.445308

1.419167 1.422035 1.534692 1.375549 1.431889 1.589421 1.366549

1.370479 1.386987 1.388271 1.408037 1.381393 1.536887 1.477040

1.540622 1.663777 1.961767 1.465546 1.485730 1.459197 1.510913

1.444826 1.552370 1.572988 1.409395 1.355619 1.320222 1.529387

1.375265 1.570786 1.410909 1.535036 1.540678 1.016811 0.000000

0.000010

TrC 1.754986 1.728221 1.640797 1.629985 1.269503 1.574753

1.546710 1.794823 1.738978 1.671900 1.990165 2.406692 1.990751

1.956686 1.423375 1.428856 1.423375 1.423375 1.422968 1.423972

1.416023 1.438032 1.393100 1.398805 1.397962 1.450973 1.691218

1.428428 1.507761 1.489034 1.529039 1.480710 1.479522 1.475101

1.464839 1.448704 1.445306 1.354668 1.450811 1.421487 1.401252

1.474850 1.526309 1.475604 1.513466 1.484520 1.463579 1.469119

1.479240 1.444613 1.371884 1.427267 1.363350 1.528562 1.445308

1.419167 1.422035 1.534692 1.375549 1.431889 1.589421 1.366549

1.370479 1.386987 1.388271 1.408037 1.381393 1.536887 1.477040

1.540622 1.663777 1.961767 1.465546 1.485730 1.459197 1.510913

1.444826 1.552370 1.572988 1.409395 1.355619 1.320222 1.529387

1.375265 1.570786 1.410909 1.535036 1.540678 1.016811 0.000010

0.000000
